# Identification of Syndrome Types in Patients With Pancreatic Cancer From Free Text in Electronic Medical Records: Model Development and Validation

**DOI:** 10.2196/70602

**Published:** 2025-10-03

**Authors:** He Ba, Haojie Du, Chienshan Cheng, Yuan Zhang, Linjie Ruan, Zhen Chen

**Affiliations:** 1 Qingdao Institute Department of Integrative Oncology Fudan University Shanghai Cancer Center Qingdao China; 2 Department of Oncology Shanghai Medical College Fudan University Shanghai China; 3 Key Laboratory of Surface & Interface Science of Polymer Materials of Zhejiang Province School of Chemistry and Chemical Engineering Zhejiang Sci-Tech University Hangzhou China; 4 Shengzhou Innovation Research Institute of Zhejiang Sci-Tech University Shengzhou China; 5 Department of Integrative Oncology Fudan University Shanghai Cancer Center Shanghai China

**Keywords:** pancreatic cancer, traditional Chinese medicine, syndrome differentiation, BERT, natural language processing, bidirectional encoder representations from transformers

## Abstract

**Background:**

Syndrome differentiation is crucial in traditional Chinese medicine (TCM) diagnosis and treatment, but it heavily relies on expert experience, limiting systematic standardization.

**Objective:**

This study developed and validated a BERT (bidirectional encoder representations from transformers)–based model, the traditional Chinese medicine pancreatic cancer syndrome differentiation bidirectional encoder representations from transformers (TCMPCSD-BERT), using in-house pancreatic cancer medical records, to digitalize expert knowledge and support standardized syndrome differentiation in TCM.

**Methods:**

A retrospective dataset of pancreatic cancer cases (2011-2024) from Fudan University Shanghai Cancer Center was annotated into 4 TCM syndrome types by 2 experts (Cohen κ=0.913). The proposed TCMPCSD-BERT model was compared with conventional models (long short-term memory and text convolutional neural network) embedded in TCM diagnostic tools and with large language models (LLMs; ChatGPT-4o, Kimi, Ernie Bot 4.0 Turbo, and Zhipu Qingyan) under a prompt engineering framework. Performance evaluation on in-house data was supplemented with attention visualizations and integrated gradients analyses for interpretability. The McNemar test assessed classification accuracy differences, while bootstrap 95% CIs quantified statistical uncertainty and stability. The Welch *t* test (2-tailed) was used to evaluate mean differences between TCMPCSD-BERT and the comparator models.

**Results:**

Among 6830 records, case counts were damp-heat syndrome (n=1694), spleen-deficiency syndrome (n=1185), damp-heat with spleen-deficiency syndrome (n=1178), and others (n=2773). On the test set, McNemar test showed significantly higher accuracy for TCMPCSD-BERT than the 3 baseline models and generally better performance than LLMs. In all comparisons, TCMPCSD-BERT achieved higher mean macroprecision, macrorecall, macro–*F*_1_-score, and accuracy, with nonoverlapping 95% bootstrap CIs and significant Welch *t* test results (*P*<.01). The model achieved a macroprecision of 0.935 (95% CI 0.918-0.951), macrorecall of 0.921 (95% CI 0.900-0.942), macro–*F*_1_-score of 0.927 (95% CI 0.908-0.945), and accuracy of 0.919 (95% CI 0.899-0.939). Attention visualizations suggested the model could capture less common TCM term associations, while integrated gradients highlighted high-attribution diagnostic features (eg, “gray-white stool” 0.933 in damp-heat syndrome; “indigestion” 1.204 in spleen-deficiency syndrome). Misclassification analyses indicated challenges in handling overlapping or atypical symptom presentations. Compared with LLMs, web-based platforms, and diagnostic instruments, TCMPCSD-BERT appeared to provide relatively higher accuracy, interpretability, and efficiency in processing long unstructured texts for syndrome differentiation.

**Conclusions:**

The TCMPCSD-BERT model shows potential for automated syndrome differentiation from unstructured clinical texts and broader application in TCM. Based on this study, it appears to improve operability over 4-diagnostic instruments and web-based platforms, and offers greater stability and accuracy than LLMs in specific tasks. However, these findings should be interpreted cautiously, given the subjectivity of syndrome definitions, data imbalance, and reliance on preprocessed, expert-annotated data. Further studies involving larger and more diverse populations are needed to validate its generalizability and support its broader application in real-world settings.

## Introduction

### Background

In recent years, traditional Chinese medicine (TCM) has been explored as a potential adjunctive approach in the integrative management of pancreatic cancer, particularly within clinical settings in China and other East Asian countries [1,2]. Some small-scale clinical studies have suggested that TCM interventions may help relieve symptoms and improve quality of life for patients with pancreatic cancer under specific conditions [3-5]. A core principle of TCM is “treatment based on syndrome differentiation,” which emphasizes tailoring therapies to the patient’s individualized symptom profile. In clinical practice, this process involves evaluating a patient’s tongue appearance, pulse characteristics, mental state, and other observable signs to classify them into specific syndrome types, forming the basis for treatment selection [6]. However, the process of syndrome differentiation in TCM is still largely grounded in traditional Chinese philosophical theories, which are abstract and holistic in nature. It remains heavily dependent on the clinician’s subjective interpretation, thereby introducing considerable variability into diagnostic outcomes. While TCM continues to play a role in culturally embedded health practices, reducing the subjectivity inherent in syndrome differentiation and improving its standardization and reproducibility remain key challenges in the ongoing development of TCM diagnostic methodology.

In recent years, the application of deep learning technologies within the field of TCM has grown significantly, opening up new possibilities for the digital transformation of TCM diagnostics and treatment. In the field of natural language processing (NLP), the BERT (bidirectional encoder representations from transformers) model has emerged as a highly effective tool for semantic understanding due to its bidirectional encoding and self-attention mechanisms [7,8]. As a third-generation NLP model, BERT marks a considerable advancement over earlier approaches. Traditional first-generation models, such as multilayer perceptrons [9], support vector machines [10], and XGBoost (extreme gradient boosting) [11], perform well with structured data, particularly when dealing with explicit textbook features. However, they struggle to handle the contextual semantics present in unstructured clinical records. Second-generation models, such as long short-term memory (LSTM) [12] and text convolutional neural networks (text-CNN) [13], introduced sequence-processing capabilities. While LSTM preserves long-term dependencies through memory cells, and text-CNN focuses on local features for tasks such as phrase extraction, both models fall short in providing a global understanding of semantics. In contrast, BERT’s self-attention mechanism constructs semantic associations between words, capturing long-range dependencies and achieving a comprehensive understanding of context, critical for processing unstructured TCM case texts. This allows BERT to significantly enhance the accuracy and precision of differentiation tasks, treating words as though “distance does not matter.”

In recent years, several general-purpose large language models (LLMs), such as ChatGPT [14], Ernie Bot [15], and Kim [16], as well as TCM-specific LLMs, including Zhipu Qingyan [17], have been developed and widely adopted. These models, leveraging powerful computational capabilities and extensive knowledge bases, have demonstrated significant potential in advancing the digital transformation of clinical diagnostics and treatment. However, challenges such as the “hallucination problem” [18] and “long-text forgetting” [19] remain common issues faced by large models across various application domains, significantly affecting their accuracy and stability in syndrome differentiation tasks. Compared to modern medicine, the availability of public clinical case corpora in the field of TCM is relatively scarce, and most TCM case records have not been systematically annotated. For LLMs, training requires a large amount of high-quality annotated data to enhance their performance in specific domains, but the limited availability of TCM-specific data severely restricts the application and accuracy of models in TCM-related tasks. Additionally, the complexity of TCM terminology and its unique linguistic style pose additional challenges for the debugging and application of LLMs. Many TCM terms are rarely encountered in modern medical literature, and some terms appear in a semiclassical Chinese format, making it difficult for models to accurately understand and process these terms. As a result, LLMs often struggle to correctly recognize and comprehend TCM terms within clinical cases, leading to potential inaccuracies in syndrome differentiation results, which in turn affects the reliability of clinical decision-making.

Furthermore, despite the release of several syndrome differentiation platforms [20] and 4-diagnostic instruments [21-23] in the field of TCM, these web-based platforms and offline hardware still rely primarily on structured input due to limitations in computational power and memory. This structured input method often requires manual intervention and preprocessing, which results in limited operability and flexibility. In TCM clinical practice, different diseases possess unique diagnostic characteristics, meaning that the same symptoms may have significantly different implications for syndrome differentiation depending on the disease. For example, when inputting syndrome differentiation elements for pancreatic cancer “damp-heat syndrome (DH),” the platform might mistakenly identify it as a different disease, such as a hepatitis-related syndrome, failing to accurately assess the pancreatic cancer syndrome content, leading to a lack of diagnostic specificity in the output. In contrast, the BERT-based end-to-end approach holds the promise of achieving more accurate, efficient, and specific performance for the task of syndrome differentiation in pancreatic cancer.

### Objective

This study aims to explore the feasibility of a BERT-based model, traditional Chinese medicine pancreatic cancer syndrome differentiation bidirectional encoder representations from transformers (TCMPCSD-BERT), trained on 6830 clinical records, in supporting syndrome classification in pancreatic cancer. The model was developed to explore whether deep learning techniques may help mitigate subjectivity and enhance the operability of syndrome differentiation from unstructured TCM narratives. Its performance was evaluated against conventional neural network models commonly embedded in existing TCM diagnostic tools [21-23], as well as several representative LLMs under a prompt-based framework.

## Methods

### TCMPCSD-BERT Model Training Pipeline

**Figure 1:**
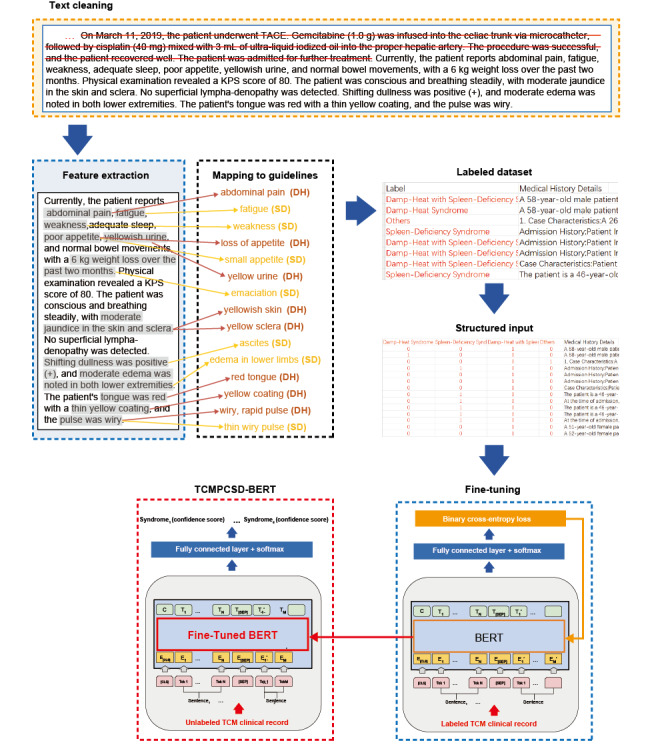
Training pipeline of TCMPCSD-BERT for syndrome differentiation in pancreatic cancer. TCMPCSD-BERT: traditional Chinese medicine pancreatic cancer syndrome differentiation bidirectional encoder representations from transformers.

The training pipeline of the TCMPCSD-BERT model is illustrated in Figure 1, which outlines the key procedures involved in the model’s development. Each module shown in the figure corresponds to a specific methodological component, which is described in further detail in the subsequent subsections of the Methods section. The process began with text cleaning, during which clinical narratives were manually reviewed to remove historical descriptions and resolved symptoms considered irrelevant to the current syndrome classification task. As indicated by the red strikethroughs in Figure 1, only current symptoms and select historical details with potential diagnostic relevance were retained to facilitate the model’s learning of syndrome-related patterns (see “Data Source and Patient Population” and Multimedia Appendix 1 for details). Subsequently, relevant diagnostic features were extracted from the cleaned unstructured texts and mapped to standardized syndrome elements based on established TCM diagnostic guidelines. These steps are illustrated in Figure 1 through shaded text segments and color-coded arrows (see “Data Source and Patient Population” and “Multimedia Appendix 1” for details). Based on these extracted and mapped features, all records were labeled according to corresponding syndrome categories. The annotated records were then transformed into 1-hot encoded vectors to meet the model’s input requirements. During the training phase, the model was fine-tuned using a fully connected output layer followed by a binary cross-entropy loss function, which optimized the model parameters for a multilabel classification task. As shown in Figure 1, the loss function computed discrepancies between predicted and actual labels and updated the model weights via backpropagation (highlighted in orange), gradually improving classification performance over successive iterations (see “TCMPCSD-BERT Model Architecture and Training Strategy” and Multimedia Appendix 2 for details). Following training, the optimized model was preliminarily applied to infer syndrome types from previously unseen TCM clinical texts. When presented with previously unseen, unlabeled clinical narratives, the model inferred syndrome labels along with associated confidence scores, using the optimized parameters fixed during training (highlighted in red in Figure 1).

As all clinical narratives used in training were originally written in Chinese, the examples in Figure 1 have been translated into English for illustrative clarity. A detailed Chinese-English mapping of figure components is available in Table S1 (Multimedia Appendix 3).

### Data Source and Patient Population

This study retrospectively collected clinical records of patients with pancreatic cancer hospitalized between January 2011 and September 2024 from the electronic health record system of the Department of Integrative Oncology at Fudan University Shanghai Cancer Center. To ensure patient privacy, all records were rigorously deidentified and anonymized, removing personally identifiable information such as names, ID numbers, contact details, and medical record numbers. The data were used exclusively for research purposes, with no commercial use or public release.

This study included only patients with pathologically confirmed pancreatic cancer. Individuals diagnosed with pancreatic neuroendocrine tumors or nonpancreatic primary malignancies were excluded. In constructing the TCM syndrome classification model, we selected only those syndrome types that were represented by at least 500 cases (for example, DH was included only if it had 500 or more records). Cases that did not clearly exhibit a recognizable syndrome pattern according to established diagnostic criteria were grouped under the category “others.” Likewise, syndrome types with insufficient sample sizes—such as blood-stasis syndrome, which was represented by fewer than 500 records—were also collectively categorized as “others.” This inclusion threshold was primarily based on the potential modeling limitations that deep learning architectures may encounter under conditions of class imbalance.

Previous studies [24-26] have suggested that in BERT-based text classification tasks, the semantic features of a minority class may not be adequately learned when the number of training examples for that class is substantially lower than that of the others. Specifically, because the BERT model is typically trained by minimizing an overall loss function such as cross-entropy, classes with fewer samples contribute relatively little to the gradient updates. This may lead the model to focus disproportionately on majority classes during training, while underrepresenting or even neglecting minority classes during inference, thereby resulting in notably reduced recall for those categories. Moreover, underrepresented classes often lack sufficient contextual diversity to support robust representation learning, which may compromise the model’s ability to capture distinctive linguistic patterns associated with these categories and, in turn, affect its overall classification stability and generalizability. In light of these considerations, and to ensure sufficient training signal for learning reliable representations, this study prioritized the inclusion of relatively well-represented syndrome types as classification targets.

This study followed the syndrome differentiation criteria for pancreatic cancer outlined in “Integrated Chinese and Western Medicine in Oncology” [27] and “Principles of Syndrome Differentiation and Treatment in TCM” [28]. Two TCM physicians, each with over 10 years of clinical experience, independently reviewed each case and performed feature annotation. To evaluate the consistency between the 2 experts’ annotations, this study, following relevant research [29], used Cohen κ coefficient as a quantitative measure. In instances where discrepancies or ambiguities arose between the 2 experts’ annotations, a third TCM physician—also with more than 10 years of clinical experience—was invited to participate in the review. The 3 physicians jointly re-examined the case and engaged in thorough discussion until a consensus was reached, which was then adopted as the final annotation for that case. Detailed information on feature engineering and data preprocessing can be found in Multimedia Appendix 1.

### TCMPCSD-BERT Model Architecture and Training Strategy

The TCMPCSD-BERT model is based on the BERT architecture and effectively captures semantic relationships and contextual information of vocabulary through input embeddings, positional encoding, and self-attention mechanisms. In the masked language model task, the model randomly masks certain tokens, forcing it to predict the missing words using contextual information, thereby enhancing its understanding of the text’s context. Through iterative optimization of parameters, the model progressively improves its accuracy in identifying TCM syndrome features. Ultimately, by incorporating fine-tuning and a multiclass classification output layer, TCMPCSD-BERT achieves the automatic identification and classification of syndrome features in the clinical texts of patients with pancreatic cancer. Detailed information on the TCMPCSD-BERT model architecture and training strategy can be found in Multimedia Appendix 2.

### Experiment Setup

All algorithms in this study were implemented using Python (version 3.12; Python Software Foundation). The experimental hardware configuration included an NVIDIA RTX 4090 GPU (25.2 GB VRAM), a 16-core AMD EPYC 9354 CPU, and 60 GB of memory.

Based on the proportion of case records for each syndrome type, the dataset was stratified and randomly split into a training and validation set (6147 entries) and a test set (683 entries) at a ratio of 9:1, ensuring that the distribution of classes in the training, validation, and test sets was consistent with the original dataset. Given that BERT model training requires a substantial amount of data, the 6147 text entries constitute a relatively limited dataset. To improve the generalization capability and stability of the model, 10-fold cross-validation was used. Specifically, the dataset was randomly divided into 10 subsets, with 9 subsets used for training and 1 subset for validation in each iteration. This process was repeated 10 times, allowing each subset to serve as the validation dataset once. The final model performance was calculated as the average of all validation results.

The training procedure followed the BERT multiclass classification fine-tuning strategy proposed by Sun et al [30] in 2019, incorporating their methods for data preprocessing and hyperparameter adjustment. The Adam optimizer was selected for optimization, as it dynamically adjusts the learning rate based on gradient variations, facilitating faster convergence and improved model performance. To prevent overfitting, we implemented dropout regularization, which enhances model generalizability by randomly deactivating a subset of neurons during training.

For hyperparameter tuning, we used grid search to systematically adjust parameters such as learning rate, batch size, and number of training epochs. Each combination was evaluated on the validation dataset, with the combination yielding the highest accuracy and *F*_1_-score selected to optimize the model’s performance in the syndrome differentiation task.

To assess the model’s performance in syndrome differentiation for this multiclass classification task, the metrics of macroprecision, macrorecall, macro–*F*_1_-score, and accuracy were used, as recommended in previous studies [31].

### Model Baseline

In line with previous studies [21-23], this research uses LSTM and text-CNN as baseline models. To ensure consistency in experimental design, these baseline models were trained using the same 10-fold cross-validation approach as TCMPCSD-BERT, with identical training and validation datasets. Additionally, a BERT model without fine-tuning on clinical data was included as a baseline. We assessed the performance of TCMPCSD-BERT, the non–fine-tuned BERT, LSTM, and text-CNN models on the same test dataset, comparing macroprecision, macrorecall, macro–*F*_1_-score, and accuracy metrics to provide a comprehensive evaluation of each model’s effectiveness in the TCM syndrome differentiation task. The detailed definitions and calculation procedures for these 4 evaluation metrics are provided in Multimedia Appendix 4.

To further examine whether the observed performance differences between models were statistically significant, and in line with prior studies using similar evaluation approaches [32], we applied McNemar test for pairwise comparison of classification results. Given the multiclass nature of the task, a “one-vs-rest” strategy was used to convert each syndrome type into a binary classification problem, allowing for class-wise analysis. For each syndrome label, a 2×2 contingency table was constructed to compare TCMPCSD-BERT with each baseline model. When the number of discordant predictions (b+c) was ≥25, the chi-square approximation without continuity correction was used [33]; otherwise, the exact binomial test was applied for small sample sizes (b+c<25) [34]. Further statistical details are provided in Multimedia Appendix 4.

To more comprehensively assess the stability and statistical uncertainty of each model’s classification performance, we adopted a stratified bootstrap resampling strategy based on prior studies [35,36]. Specifically, for each model, we conducted 5000 iterations of resampling with replacement from the test set predictions. For each bootstrap sample, we computed 4 evaluation metrics: macroprecision, macrorecall, macro–*F*_1_-score, and accuracy. Based on the resulting 5000 estimates per metric, we used the percentile bootstrap method to construct 95% CIs (bootstrap 95% CIs) and calculated the corresponding bootstrap means, thereby quantifying the variability and uncertainty in model performance. Further statistical details are provided in Multimedia Appendix 4.

Furthermore, given that all models were evaluated on the same test set and their prediction results were mutually independent, we conducted independent samples *t* tests to examine whether the observed differences between TCMPCSD-BERT and other models were statistically significant across the evaluation metrics [37]. When the bootstrap estimates approximately satisfied the assumptions of normality and homogeneity of variance, the standard independent samples *t* test was applied. In cases where unequal variances were observed, Welch *t* test was used instead. For metrics whose distributions substantially deviated from normality and could not be normalized through transformation, the nonparametric Mann-Whitney *U* test was used as an alternative. Further statistical details are provided in Multimedia Appendix 4.

### Visualization of the Self-Attention Mechanism in TCMPCSD-BERT for Syndrome Differentiation

In order to gain a deeper insight into how the TCMPCSD-BERT model captures word associations in the task of syndrome differentiation, this study adopts a visualization approach based on the self-attention mechanism, as proposed in previous research [38]. The model’s performance was examined by processing clinical texts associated with 3 distinct syndrome types: DH, “spleen-deficiency syndrome,” and “damp-heat with spleen-deficiency syndrome (DH-SD).” The visualization specifically focuses on the attention distribution within the model’s 0th layer, across all 12 layers, to provide a detailed understanding of how the model attends to different vocabulary elements in these syndrome differentiation tasks.

### Explanation of Syndrome Differentiation Decisions

To further quantify the model’s reliance on specific symptoms and signs in the syndrome differentiation task, we applied the integrated gradients algorithm [39] to assess each word’s contribution to syndrome prediction in the clinical record text. This method generates an attribution score for each word in the case, reflecting its importance in predicting the syndrome type. Words that are more critical to the syndrome differentiation will receive higher attribution scores, while symptoms with lower or negligible impact on the prediction will receive lower attribution scores. A more detailed explanation, setup, and interpretation of the integrated gradients algorithm can be found in Multimedia Appendix 5.

### LLMs Prompt Engineering

In terms of prompt design, this study drew inspiration from the MedPrompt strategy proposed by the Microsoft team in 2023 [40] and recent similar studies [41,42], integrating the characteristics of TCM syndrome differentiation tasks to develop an initial prompt scheme. The key components include “role-play prompting,” “in-context learning,” “chain-of-thought,” and “choice shuffling ensemble.” To address issues related to context forgetting and model hallucination when handling long text tasks, this study reconstructs the dialogue interface with every 15 questions, reloads the task context and rules, and repeats the processes of in-context learning, chain-of-thought, and choice shuffling ensemble until all cases have completed syndrome differentiation [43].

To evaluate the performance of LLMs in the syndrome differentiation task, this study adopted the same stratified test set as used for TCMPCSD-BERT and other specialized models, ensuring that the class distribution in the test set was consistent with the original dataset. The test set split followed the procedure described in the “Experiment Setup” section of the Methods. Detailed settings can be found in Multimedia Appendices 6 and 7.

To assess whether the performance differences between TCMPCSD-BERT and the LLMs were statistically significant, we adopted the same evaluation framework as described for the baseline model comparisons. McNemar test was applied using a “one-vs-rest” strategy to conduct pairwise comparisons for each syndrome category. Depending on the number of discordant cases, either the chi-square approximation or the exact binomial test was used, as appropriate.

Additionally, stratified bootstrap resampling (5000 iterations) was used to quantify the uncertainty of model performance. For each resampled dataset, macroprecision, macrorecall, macro–*F*_1_-score, and accuracy were computed. The percentile method was used to construct bootstrap 95% CIs, and bootstrap means were calculated to summarize the metric distributions. Further statistical details are provided in Multimedia Appendix 4.

Subsequently, independent samples *t* tests were performed on the 5000 bootstrap estimates to examine whether the observed differences between TCMPCSD-BERT and the LLMs were statistically significant. Welch *t* test or the Mann-Whitney *U* test was used when assumptions of normality or homogeneity of variance were not met. Further statistical details are provided in Multimedia Appendix 4.

### Comparison of Unstructured Long-Text Processing and Syndrome Differentiation Specificity for Pancreatic Cancer Between TCMPCSD-BERT and Web-Based Platforms

We selected a recently published online syndrome differentiation platform [20] and input structured syndrome differentiation elements from clinical cases corresponding to DH, “spleen-deficiency syndrome,” and DH-SD into the platform to observe whether it could accurately differentiate pancreatic cancer. Subsequently, we input the unstructured text of each clinical case into the platform to evaluate its performance in syndrome differentiation. This process allowed us to assess the specificity and accuracy of syndrome differentiation for pancreatic cancer by the web-based platform in comparison to TCMPCSD-BERT.

### Ethical Considerations

This study was approved by the Ethics Committee of the Department of Oncology, Fudan University (approval number 2012228-17 and 2509-Exp282). All procedures were conducted in accordance with the ethical standards of the institutional research committee and with the 1964 Helsinki Declaration and its later amendments or comparable ethical standards. Given the retrospective design of this study and the complete anonymization of the data used, the requirement for informed consent was waived according to institutional policy. Furthermore, as no direct patient participation or interventions were involved, the requirement for participant compensation was also waived.

## Results

### Dataset and Model Training Details

This study used a total of 6830 clinical records from 1980 patients with pancreatic cancer for model training and evaluation. The dataset included 1694 records labeled as DH, 1185 records as spleen-deficiency syndrome, and 1178 records as DH-SD. An additional 2773 records were categorized as “others,” which encompassed cases without vivid syndrome differentiation and syndromes with fewer than 500 records, thus not fulfilling the inclusion criteria. The mean age of the patients was 61 (SD 8.4) years in the DH group, 62 (SD 7.7) years in the spleen-deficiency syndrome group, 62 (SD 8.6) years in the DH-SD group, and 61 (SD 8.5) years in the others group. Among all patients, 39.4% (2688/6830) were female, 80.9% (5526/6830) had stage III–IV disease, and 46.7% (3189/6830) received chemotherapy. Detailed baseline characteristics are presented in Table 1. Based on the interrater agreement analysis using Cohen κ coefficient, the value for the syndrome differentiation task in this study was 0.913, indicating a high level of agreement between the 2 experts. The detailed contingency table for the Cohen κ analysis of the experts’ annotations is provided in Table S4 of Multimedia Appendix 8.

Throughout the model training process, extensive hyperparameter tuning was performed for each model, with performance evaluated using the validation dataset. This process allowed for the identification of optimal hyperparameter configurations for each model, achieving the highest accuracy and *F*_1_-score for the syndrome differentiation task. The optimal training parameters for each model are detailed in Table S2 of Multimedia Appendix 8.

**Table 1:** Descriptive statistics of the cancer cohort.

Descriptive statistics			Damp-heat syndrome (1694/6830, 24.8%)		Spleen-deficiency syndrome (1185/6830, 17.3%)	Damp-heat with spleen-deficiency syndrome (1178/6830, 17.2%)		Others (2773/6830, 40.6%)
**Demographics**								
	Sex (female), n/N (%)	678/1694 (40)		484/1185 (40.8)		447/1178 (37.9)	1079/2773 (38.9)	
	Age (years), mean (SD)	61 (8.4)		62 (7.7)		62 (8.6)	61 (8.5)	
**Cancer stage, n/N (%)**								
	Stage I	110/1694 (6.5)		55/1185 (5)		87/1178 (7)	263/2773 (9.5)	
	Stage II	200/1694 (11.8)		122/1185 (10.3)		95/1178 (8)	372/2773 (13.4)	
	Stage III	776/1694 (45.8)		560/1185 (47.3)		531/1178 (45)	918/2773 (33.1)	
	Stage IV	608/1694 (35.9)		448/1185 (37.8)		465/1178 (39.5)	1220/2773 (44)	
**Chemotherapy, n/N (%)**								
	Yes	791/1694 (46.7)		511/1185 (43.1)		487/1178 (41.3)	1400/2773 (50.5)	
	No	903/1694 (53.3)		674/1185 (56.9)		691/1178 (58.7)	1373/2773 (49.5)	
**Diabetes, n/N (%)**								
	Yes	251/1694 (14.8)		145/1185 (12.2)		129/1178 (11)	385/2773 (13.9)	
	No	1443/1694 (85.2)		1040/1185 (87.8)		1049/1178 (89)	2388/2773 (86.1)	
**Hypertension, n/N (%)**								
	Yes	249/1694 (14.7)		182/1185 (15.4)		270/1178 (22.9)	613/2773 (22.1)	
	No	1445/1694 (85.3)		1003/1185 (84.6)		908/1178 (77.1)	2160/2773 (77.9)	
**Smoking status, n/N (%)**								
	Never	302/1694 (17.8)		198/1185 (16.7)		226/1178 (19.2)	348/2773 (12.6)	
	Former	1279/1694 (75.5)		916/1185 (77.3)		843/1178 (71.6)	2246/2773 (81)	
	Current	113/1694 (6.7)		71/1185 (6)		109/1178 (9.2)	179/2773 (6.4)	
**Alcohol consumption, n/N (%)**								
	Never	120/1694 (7.1)		137/1185 (11.6)		144/1178 (12.2)	187/2773 (6.7)	
	Occasionally	115/1694 (6.8)		56/1185 (5)		80/1178 (7)	68/2773 (3)	
	Regularly	1459/1694 (86.1)		992/1185 (83.7)		954/1178 (81)	2518/2773 (90.8)	

### Performance Comparison of TCMPCSD-BERT and Baseline Models

**Figure 2:**
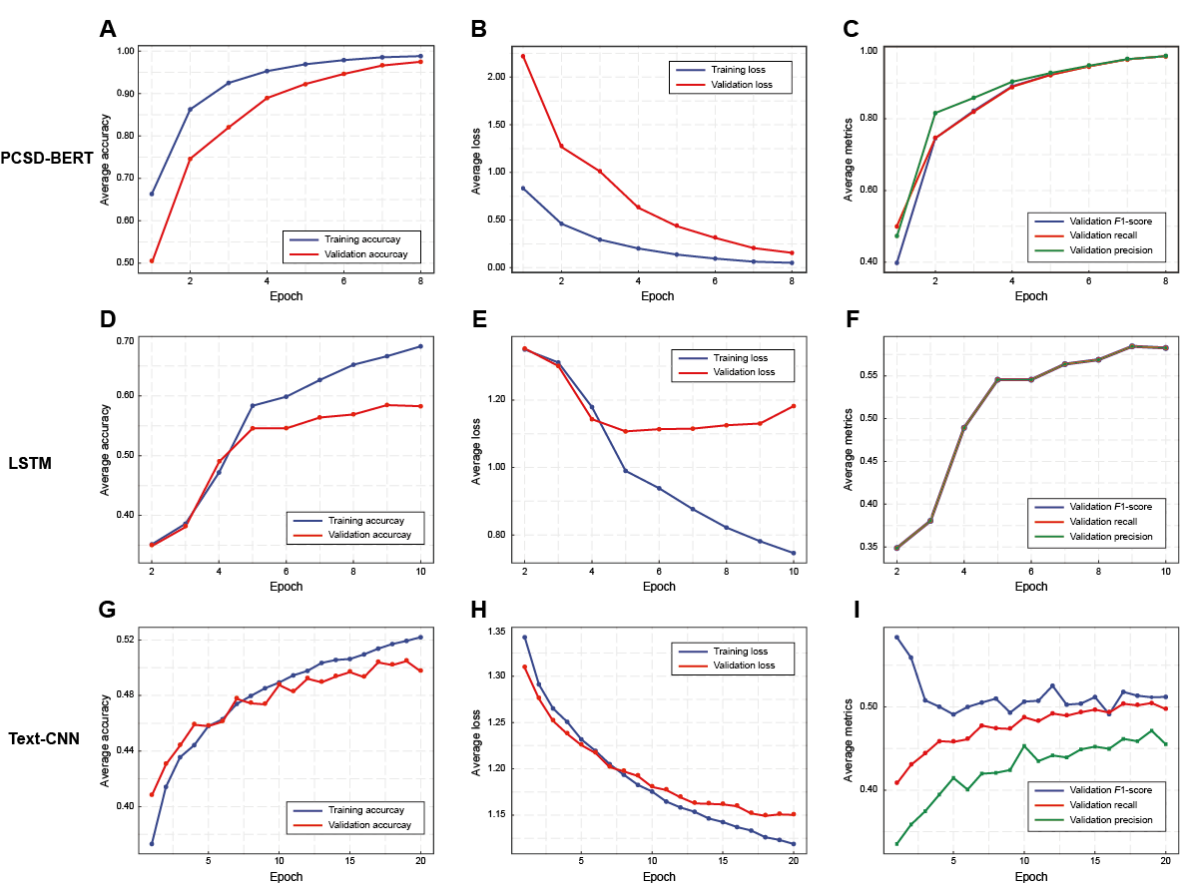
Training and validation performance curves of TCMPCSD-BERT, LSTM, and text-CNN models. (A) Training and validation accuracy curves for TCMPCSD-BERT. (B) Training and validation loss curves for TCMPCSD-BERT. (C) Validation F1-score, recall, and precision curves for TCMPCSD-BERT. (D) Training and validation accuracy curves for LSTM. (E) Training and validation loss curves for LSTM. (F) Validation F1-score, recall, and precision curves for LSTM. (G) Training and validation accuracy curves for text-CNN. (H) Training and validation loss curves for text-CNN. (I) Validation F1-score, recall, and precision curves for text-CNN. LSTM: long short-term memory; TCMPCSD-BERT: traditional Chinese medicine pancreatic cancer syndrome differentiation bidirectional encoder representations from transformers; text-CNN: text convolutional neural network.

As illustrated in Figure 2, TCMPCSD-BERT demonstrated superior syndrome differentiation performance in both training and validation phases. With increasing epochs, the accuracy of the training and validation datasets improved steadily, with validation accuracy closely approaching training accuracy. This pattern indicates the model’s strong generalization capacity and the absence of overfitting (Figure 2A). Simultaneously, the training and validation losses decreased in parallel, reflecting good convergence and the model’s effectiveness in capturing critical features for the TCM syndrome differentiation task (Figure 2B). The *F*_1_-score, recall, and precision metrics on the validation dataset progressively improved throughout training, ultimately reaching approximately 0.97, underscoring the model’s high accuracy and reliability in syndrome differentiation (Figure 2C).

In contrast, the LSTM model displayed clear signs of overfitting. As the number of epochs increased, training accuracy approached 0.7, while validation accuracy rose slowly and consistently remained lower than training accuracy, indicating limited generalization on the validation dataset (Figure 2D). Training loss continued to decrease, whereas validation loss plateaued or slightly increased, further confirming overfitting (Figure 2E). The *F*_1_-score, recall, and precision of the LSTM model stabilized at around 0.55 after an initial increase, revealing limited classification capability (Figure 2F).

For the text-CNN model, training accuracy increased with additional epochs but plateaued around 0.52 after approximately 20 epochs. Validation accuracy exhibited limited improvement and fluctuated considerably, peaking near 0.48 but consistently remaining below training accuracy, suggesting a tendency toward overfitting (Figure 2G). Although training loss declined significantly, validation loss leveled off after an initial drop, remaining slightly higher than training loss and indicating insufficient generalization on the validation dataset (Figure 2H). The *F*_1_-score, recall, and precision on the validation dataset were both low and unstable, highlighting text-CNN’s limited classification capability in syndrome differentiation. The substantial fluctuations in these metrics suggest inconsistent performance across different validation folds, hindering text-CNN’s ability to establish stable differentiation rules (Figure 2I).

As shown in Table 2, McNemar test suggests that TCMPCSD-BERT demonstrated significantly better classification performance than the 3 baseline models in distinguishing all 4 TCM syndrome types. As shown in Table 3, when compared with the traditional baseline models, TCMPCSD-BERT achieved higher mean values across all 4 evaluation metrics. The 95% bootstrap CIs for TCMPCSD-BERT did not overlap with those of the comparator models. The results from Welch *t* test indicated that these differences were statistically significant in all comparisons (*P*<.01).

**Table 2:** McNemar test comparing TCMPCSD-BERT^a^ and baseline models across syndrome categories.

Variable			TCMPCSD-BERT^a^ correct only/total		Baseline models correct only/total		Chi-square (*df*)		*P* value
**TCMPCSD-BERT versus BERT^b^ without fine-tuning**									
	Damp-heat syndrome	67/169		9/169		42.75 (1)		<.001^c^	
	Spleen-deficiency syndrome	49/119		8/119		28.07 (1)		<.001^c^	
	Damp-heat with spleen-deficiency syndrome	48/118		7/118		29.09 (1)		<.001^c^	
	Others	107/277		10/277		78.77 (1)		<.001^c^	
**TCMPCSD-BERT versus LSTM^d^**									
	Damp-heat syndrome	76/169		4/169		63.01 (1)		<.001^c^	
	Spleen-deficiency syndrome	56/119		6/119		38.73 (1)		<.001^c^	
	Damp-heat with spleen-deficiency syndrome	58/118		8/118		36.38 (1)		<.001^c^	
	Others	126/277		7/277		104.69 (1)		<.001^c^	
**TCMPCSD-BERT versus text-CNN^e^**									
	Damp-heat syndrome	80/169		4/169		66.96 (1)		<.001^c^	
	Spleen-deficiency syndrome	57/119		4/119		44.33 (1)		<.001^c^	
	Damp-heat with spleen-deficiency syndrome	56/118		3/118		45.83 (1)		<.001^c^	
	Others	136/277		11/277		104.60 (1)		<.001^c^	

^a^TCMPCSD-BERT: traditional Chinese medicine pancreatic cancer syndrome differentiation bidirectional encoder representations from transformers.

^b^BERT: bidirectional encoder representations from transformers.

^c^The *P* values were calculated using the McNemar test without continuity correction.

^d^LSTM: long short-term memory.

^e^Text-CNN: text convolutional neural network.

**Table 3:** Performance metrics with bootstrap 95% CIs and statistical comparisons between TCMPCSD-BERT^a^ and baseline models.

Models and metrics			Mean (SD)		95% CIs^b^		*P* value^c^
TCMPCSD-BERT							
	Macroprecision	0.935 (0.008)		0.918-0.951		—^d^	
	Macrorecall	0.921 (0.011)		0.900-0.942		—	
	Macro–*F*_1_-score	0.927 (0.009)		0.908-0.945		—	
	Accuracy	0.919 (0.010)		0.899-0.939		—	
BERT^e^ without fine-tuning							
	Macroprecision	0.531 (0.019)		0.495-0.567		<.01^f^	
	Macrorecall	0.540 (0.021)		0.500-0.580		<.01^f^	
	Macro–*F*_1_-score	0.527 (0.020)		0.489-0.565		<.01^f^	
	Accuracy	0.548 (0.019)		0.510-0.586		<.01^f^	
LSTM^g^							
	Macroprecision	0.481 (0.019)		0.443-0.519		<.01^f^	
	Macrorecall	0.492 (0.020)		0.451-0.531		<.01^f^	
	Macro–*F*_1_-score	0.484 (0.020)		0.446-0.522		<.01^f^	
	Accuracy	0.491 (0.019)		0.454-0.529		<.01^f^	
Text-CNN^h^							
	Macroprecision	0.483 (0.020)		0.444-0.522		<.01^f^	
	Macrorecall	0.483 (0.020)		0.443-0.523		<.01^f^	
	Macro–*F*_1_-score	0.477 (0.019)		0.437-0.515		<.01^f^	
	Accuracy	0.475 (0.019)		0.436-0.511		<.01^f^	

^a^TCMPCSD-BERT: traditional Chinese medicine pancreatic cancer syndrome differentiation bidirectional encoder representations from transformers.

^b^Bootstrap 95% CIs.

^c^Compared with traditional Chinese medicine pancreatic cancer syndrome differentiation bidirectional encoder representations from transformers model.

^d^Not applicable.

^e^BERT: bidirectional encoder representations from transformers.

^f^*P* values are obtained from the Welch *t* test.

^g^LSTM: long short-term memory.

^h^Text-CNN: text convolutional neural network.

### Validation of TCMPCSD-BERT Model’s Syndrome Differentiation Performance in Practical Applications

**Figure 3:**
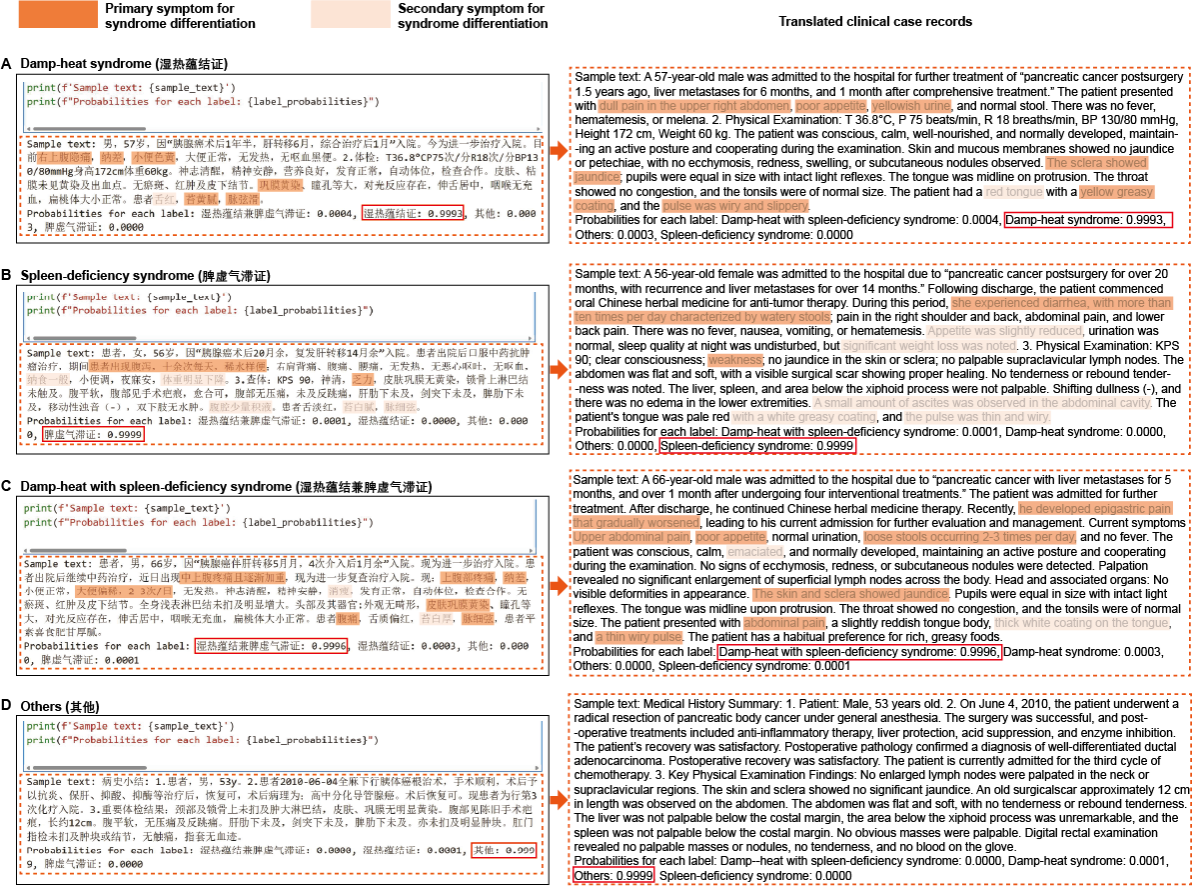
Syndrome differentiation of clinical texts by TCMPCSD-BERT. (A) A case diagnosed as “damp-heat syndrome” by clinical experts. (B) A case diagnosed as “spleen-deficiency syndrome.” (C) A case diagnosed as “damp-heat with spleen-deficiency syndrome.” (D) A case without clear syndrome differentiation features, classified as “others.” TCMPCSD-BERT: traditional Chinese medicine pancreatic cancer syndrome differentiation bidirectional encoder representations from transformers.

Upon completion of the training and performance assessment of the TCMPCSD-BERT model, its practical effectiveness in syndrome differentiation was further examined using real-world clinical cases. Cases were selected based on clinical expert diagnoses, representing 4 categories: DH, “spleen-deficiency syndrome,” DH-SD, and cases without clear syndrome differentiation (classified as “others”). These cases were input into the TCMPCSD-BERT model to evaluate its classification accuracy and interpretability. Figure 3 illustrates the practical implementation of TCMPCSD-BERT within the Jupyter Notebook environment. The “sample text” section below the code block displays the input clinical case text, and the translated clinical case records on the right provide an English version of the input clinical case text on the left for reference. The “probabilities for each label” section shows the confidence scores generated by TCMPCSD-BERT for each syndrome label. Diagnostic features within the clinical text were manually annotated under expert guidance, with primary symptoms highlighted in dark orange and secondary symptoms highlighted in light orange to enhance interpretability. For each input case, the model generated confidence scores for all syndrome labels (“probabilities for each label”), with the highest confidence score indicating the final predicted classification. To provide interpretability, diagnostic features within the clinical text were annotated under expert guidance, with primary symptoms highlighted in dark orange and secondary symptoms in light orange.

For instance, in a case diagnosed as DH, the clinical text included features such as “右上腹隐痛” (abdominal pain), “纳差” (loss of appetite), and “舌红” (red tongue), corresponding to the primary and secondary symptoms of DH. The model predicted this case as DH with a confidence score of 0.9993, consistent with the clinical expert’s diagnosis (Figure 3A). In a case diagnosed as “spleen-deficiency syndrome,” features such as “腹泻” (loose stools), “纳食一般” (small appetite), and “乏力” (fatigue) were identified. The model classified this case as “spleen-deficiency syndrome” with a confidence score of 0.9999 (Figure 3B). For a case exhibiting characteristics of both DH and “spleen-deficiency syndrome,” diagnostic features such as “腹痛” (abdominal pain) and “纳差” (loss of appetite or small appetite) were highlighted. The model predicted this case as DH-SD with a confidence score of 0.9996 (Figure 3C). Lastly, for a case without significant diagnostic features, the model correctly excluded other syndrome labels and classified it as “others” with a confidence score of 0.9999 (Figure 3D).

Table S6 in Multimedia Appendix 9 provides a detailed summary of the diagnostic features extracted from each clinical case and their correspondence with the TCM syndrome differentiation guidelines, along with their classification as primary or secondary symptoms. The table includes examples for DH and “spleen-deficiency syndrome,” listing the diagnostic features in the clinical text, their corresponding entries in the guidelines, and their classification within the differentiation framework. In all evaluated cases, the predictions generated by TCMPCSD-BERT were consistent with expert diagnoses, validating its effectiveness in practical TCM syndrome differentiation tasks.

### Visualization of Self-Attention Mechanism in TCMPCSD-BERT

**Figure 4:**
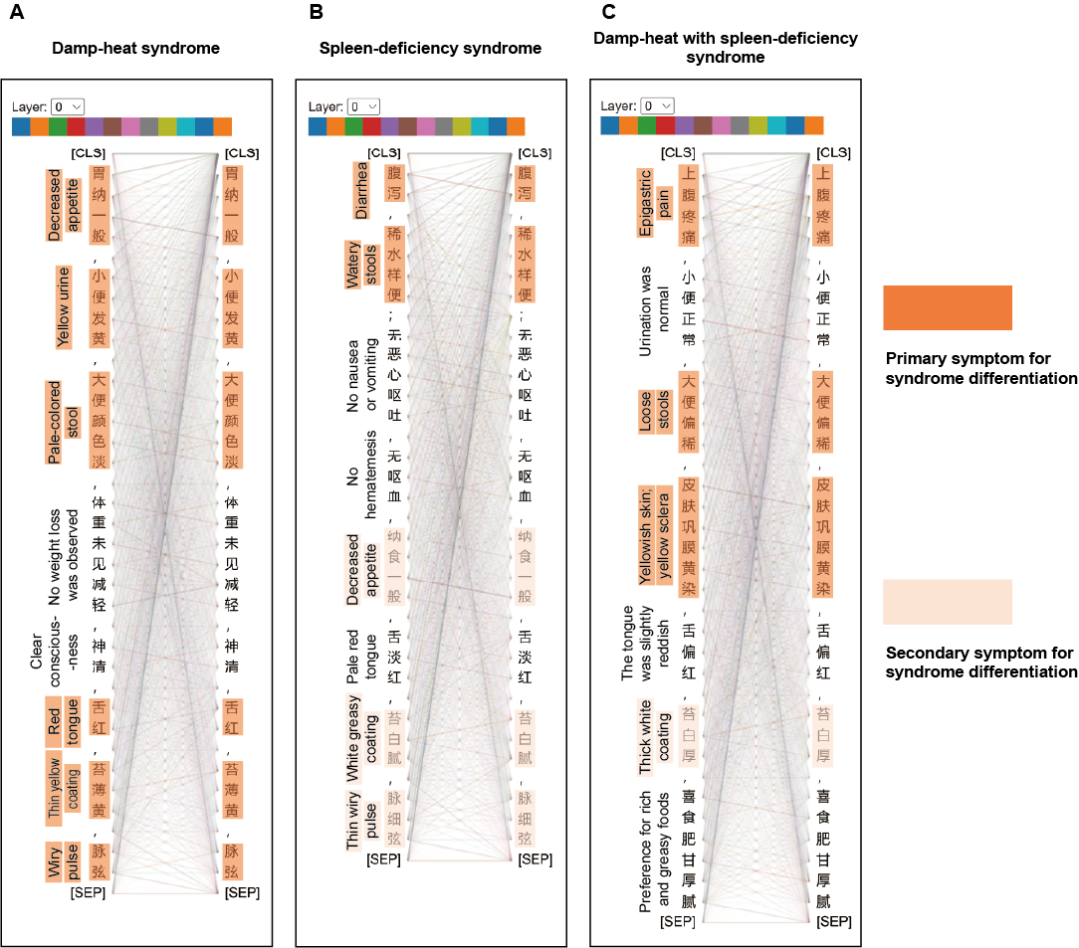
Visualization of attention distributions in TCMPCSD-BERT across syndrome texts. This figure illustrates the attention distributions in layer 0 of the TCMPCSD-BERT model for three syndrome types: (A) damp-heat syndrome, (B) spleen-deficiency syndrome, and (C) damp-heat with spleen-deficiency syndrome. CLS: classification token; SEP: separator token; TCMPCSD-BERT: traditional Chinese medicine pancreatic cancer syndrome differentiation bidirectional encoder representations from transformers.

As illustrated in Figure 4, the TCMPCSD-BERT model captures associations between vocabulary elements in clinical case texts for different syndrome types in syndrome differentiation tasks. The lines between words represent semantic associations, with the thickness of the lines reflecting the strength of these associations. Thicker lines indicate higher attention weights assigned by the model, highlighting the interdependence and significance of specific terms in syndrome differentiation. The translated clinical case records on the left provide an English version of the input clinical case text on the right for reference. Diagnostic features within the clinical text were manually annotated under expert guidance, with primary symptoms highlighted in dark orange and secondary symptoms highlighted in light orange to enhance interpretability. The [SEP] token shown in the figure is a special marker in the BERT model, used to distinguish sentences or paragraphs during text processing. This token ensures that the model can correctly identify textual boundaries and accurately interpret contextual relationships.

As shown in Figure 4A, in the text for DH, the model strengthens associations among terms such as “胃纳一般” (loss of appetite), “小便发黄” (yellow urine), “大便颜色淡” (gray-white stool), “舌红” (red tongue), “苔薄黄” (yellow coating), and “脉弦” (wiry and rapid pulse). In Figure 4B, for “spleen-deficiency syndrome,” the model highlights associations among terms such as “腹泻” (loose stools), “纳食一般” (small appetite), “舌淡红” (pale and swollen tongue), “苔白腻” (white slippery coating), and “脉细弦” (thin wiry pulse). In Figure 4C, for DH-SD, the model reinforces associations between terms such as “上腹疼痛” (abdominal pain), “大便偏稀” (loose stools), “皮肤巩膜黄染” (yellowish skin and yellow sclera), and “苔白厚” (white thick coating). This attention distribution demonstrates the model’s ability to effectively identify structural relationships between TCM-specific terms, thereby constructing a precise framework for syndrome differentiation.

Beyond associations between specific terms, we also observe that all words within the same passage exhibit a degree of interrelation. This suggests that the model considers not only the relationships among key terms but also integrates contextual links across all words, contributing to a comprehensive understanding of the overall context.

### Attribution Analysis of Word-Syndrome Associations in TCMPCSD-BERT

**Figure 5:**
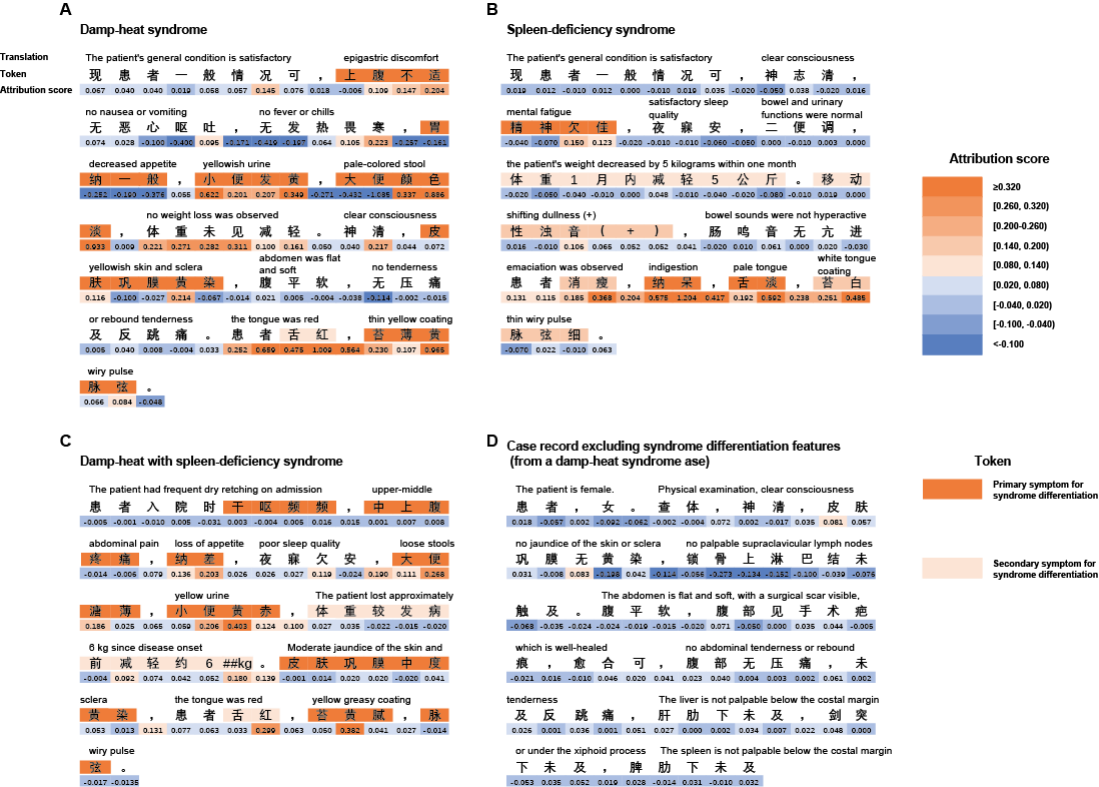
Gradient attribution analysis of TCMPCSD-BERT on syndrome texts. (A) A case diagnosed as “damp-heat syndrome” by clinical experts. (B) A case diagnosed as “spleen-deficiency syndrome.” (C) A case diagnosed as “damp-heat with spleen-deficiency syndrome.” (D) Content from a case diagnosed as “damp-heat syndrome” that does not include any diagnostic features related to syndrome differentiation. TCMPCSD-BERT: traditional Chinese medicine pancreatic cancer syndrome differentiation bidirectional encoder representations from transformers.

To evaluate the model’s ability to identify the associations between words and syndrome labels in syndrome differentiation tasks, integrated gradients were applied to clinical texts representing 3 typical syndromes: DH, “spleen-deficiency syndrome,” and DH-SD. This analysis aimed to reveal how the model assesses the importance of key diagnostic terms when interpreting clinical texts. As shown in Figure 5, the first row (translation) provides the English translation of the second row (token), which contains the original clinical case text in Chinese. The color of each token in the second row indicates its importance in real-world TCM syndrome differentiation: dark orange denotes primary diagnostic features, while light orange represents secondary features. The third row (attribution score) shows the attribution scores assigned to each token, with the values color-coded by magnitude; higher scores are represented in darker orange, indicating greater contributions to syndrome differentiation. By comparing token colors with their corresponding attribution scores, the alignment between the model’s judgments and clinical standards can be intuitively analyzed.

The TCMPCSD-BERT model assigns higher attribution scores to certain key diagnostic features. For instance, in DH, the highest attribution score for “大便颜色淡” (gray-white stool) was 0.933 (Figure 5A); in “spleen-deficiency syndrome,” the highest score for “纳呆” (indigestion) reached 1.204 (Figure 5B); and in DH-SD, the highest score for “小便黄赤” (yellow urine) was 0.403 (Figure 5C). Additionally, Figure 5D shows the attribution scores for content in the DH case that does not include diagnostic elements related to syndrome differentiation, where these terms generally exhibit lower attribution scores.

Table S7 in Multimedia Appendix 10 provides a detailed summary of the key diagnostic features extracted from each clinical text, their correspondence with the TCM syndrome differentiation guidelines, and their classification within the syndrome differentiation framework, along with attribution scores. The table highlights the highest attribution scores assigned to different diagnostic features and compares them with the average attribution scores of nondiagnostic contents. It is observed that the attribution scores for most diagnostic features are higher than those of nondiagnostic content. This distinction further validates the model’s ability to differentiate critical diagnostic features from irrelevant content. However, the attribution scores for some features deviate from clinical standards, reflecting the model’s limitations in assigning appropriate importance weights to certain features.

### Case Studies of Specific Error Patterns in TCMPCSD-BERT

The TCMPCSD-BERT model exhibited misclassification in certain cases from the test set. To gain a deeper understanding of the model’s error patterns, we selected misclassified cases from 3 typical syndromes and presented the attribution analysis results for these cases in Figure S1 (Multimedia Appendix 11).

Figure S1A in Multimedia Appendix 11 presents the attribution analysis result where the TCMPCSD-BERT model misclassified a DH case as “spleen-deficiency syndrome.” It can be observed that the model assigned lower attribution scores to key diagnostic features of DH, such as “上腹疼痛” (abdominal pain), “低热” (persistent low-grade fever), “脉弦数” (wiry and rapid pulse); while assigning higher scores to the diagnostic feature of “近2个月体重减轻2.5kg左右” (emaciation) which is associated with “spleen-deficiency syndrome.” Despite the case exhibiting multiple secondary symptoms of “spleen-deficiency syndrome” (3 secondary symptoms), the absence of a primary symptom prevented it from meeting the diagnostic criteria for “spleen-deficiency syndrome.”

Figure S1B in Multimedia Appendix 11 displays the attribution analysis results where the TCMPCSD-BERT model misclassified a “spleen-deficiency syndrome” case as DH-SD. The model assigned lower attribution scores to key diagnostic features of “spleen-deficiency syndrome,” such as “移动性浊音（+）” (ascites), “双下肢无凹陷性水肿” (edema in lower limbs), and “苔薄腻” (thin and greasy coating); while assigning higher scores to features associated with DH, such as “大便黏腻不爽、味重” (foul-smelling loose stools), and “脉弦数” (wiry and rapid pulse). Despite the case presenting 1 primary symptom and 1 secondary symptom related to DH, it failed to meet the diagnostic criteria for DH.

Figure S1C in Multimedia Appendix 11 shows the attribution analysis results where the TCMPCSD-BERT model misclassified a DH-SD case as “spleen-deficiency syndrome.” It can be observed that the model assigned lower attribution scores to the diagnostic features of “低热” (persistent low-grade fever) and “感上腹疼痛” (abdominal pain), which are key features of DH.

Table S8 in Multimedia Appendix 11 provides a detailed summary of the key diagnostic features extracted from each misclassified case’s clinical text, along with their correspondence to the TCM syndrome differentiation guidelines and their classification within the syndrome differentiation framework, as well as the attribution scores. Furthermore, a comparison is made between the attribution scores of these diagnostic features and the average attribution scores of nondiagnostic content, and the table indicates whether the model correctly assigned higher attribution scores to the diagnostic features.

### Performance Comparison Between TCMPCSD-BERT and LLMs Guided by Prompt Engineering Framework

This study further evaluated the efficacy of LLMs in differentiating syndromes in pancreatic cancer cases based on TCM principles, using a prompt engineering framework. Detailed descriptions of the prompt engineering frameworks and corresponding test cases for English- and Chinese-based LLMs are provided in the second and third sections of Multimedia Appendix 6, respectively.

As shown in Table 4, McNemar test indicates that TCMPCSD-BERT generally exhibited statistically superior classification performance compared to the evaluated LLMs across most TCM syndrome types. Although some comparisons did not reach conventional significance thresholds (eg, *P*=.07), the overall results suggest that TCMPCSD-BERT may offer more consistent differentiation performance in this task. As shown in Table 5, when compared with the LLMs, TCMPCSD-BERT achieved higher mean values across all 4 evaluation metrics, with no overlap between its bootstrap 95% CIs and those of the comparator models. Results from Welch *t* test indicated that these differences were statistically significant in all comparisons (*P*<.01).

**Table 4:** McNemar test comparing TCMPCSD-BERT^a^ and LLMs^b^ across syndrome categories.

Variable			TCMPCSD-BERT correct only/total		LLMs correct only/total		Chi-square (*df*)		*P* values
**TCMPCSD-BERT versus ChatGPT-4**									
	Damp-heat syndrome	38/169		11/169		13.80 (1)		<.001^c^	
	Spleen-deficiency syndrome	27/119		8/119		10.31 (1)		<.001^c^	
	Damp-heat with spleen-deficiency syndrome	24/118		5/118		12.45 (1)		<.001^c^	
	Others	66/277		20/277		24.60 (1)		<.001^c^	
**TCMPCSD-BERT versus ChatGPT-4o**									
	Damp-heat syndrome	27/169		14/169		4.12 (1)		<.05^c^	
	Spleen-deficiency syndrome	17/119		8/119		3.24 (1)		.07^c^	
	Damp-heat with spleen-deficiency syndrome	18/118		9/118		3.00 (1)		.08^c^	
	Others	40/277		17/277		9.28 (1)		.002^c^	
**TCMPCSD-BERT versus Ernie Bot 4.0 Turbo**									
	Damp-heat syndrome	35/169		9/169		15.36 (1)		<.001^c^	
	Spleen-deficiency syndrome	25/119		7/119		10.13 (1)		<.001^c^	
	Damp-heat with spleen-deficiency syndrome	25/118		7/118		10.13 (1)		<.001^c^	
	Others	58/277		14/277		26.89 (1)		<.001^c^	
**TCMPCSD-BERT versus Kimi**									
	Damp-heat syndrome	41/169		11/169		17.31 (1)		<.001^c^	
	Spleen-deficiency syndrome	27/119		4/119		13.36 (1)		<.001^c^	
	Damp-heat with spleen-deficiency syndrome	27/118		6/118		13.36 (1)		<.001^c^	
	Others	66/277		15/277		32.11 (1)		<.001^c^	
**TCMPCSD-BERT versus Zhipu Qingyan**									
	Damp-heat syndrome	26/169		12/169		5.16 (1)		<.05^c^	
	Spleen-deficiency syndrome	19/119		9/119		3.57 (1)		.06^c^	
	Damp-heat with spleen-deficiency syndrome	16/118		6/118		—^d^		.05^e^	
	Others	45/277		20/277		8.86 (1)		.002^c^	

^a^TCMPCSD-BERT: traditional Chinese medicine pancreatic cancer syndrome differentiation bidirectional encoder representations from transformers.

^b^LLM: large language model.

^c^The *P* values were calculated using the McNemar test without continuity correction.

^d^Not applicable.

^e^The *P* values were calculated using the exact McNemar test.

**Table 5:** Performance metrics with bootstrap 95% CIs and statistical comparisons between TCMPCSD-BERT^a^ and baseline models.

Models and metrics			Mean (SD)		95% CIs^b^		*P* value^c^
TCMPCSD-BERT							
	Macroprecision	0.935 (0.008)		0.918-0.951		—^d^	
	Macrorecall	0.921 (0.011)		0.900-0.942		—	
	Macro–*F*_1_-score	0.927 (0.009)		0.908-0.945		—	
	Accuracy	0.919 (0.010)		0.899-0.939		—	
ChatGPT-4							
	Macroprecision	0.740 (0.017)		0.706-0.774		<.001^e^	
	Macrorecall	0.752 (0.017)		0.717-0.787		<.001^e^	
	Macro–*F*_1_-score	0.744 (0.017)		0.710-0.778		<.001^e^	
	Accuracy	0.748 (0.017)		0.716-0.780		<.001^e^	
ChatGPT-4o							
	Macroprecision	0.841 (0.015)		0.811-0.869		<.001^e^	
	Macrorecall	0.846 (0.015)		0.817-0.875		<.001^e^	
	Macro–*F*_1_-score	0.843 (0.014)		0.813-0.871		<.001^e^	
	Accuracy	0.845 (0.014)		0.817-0.873		<.001^e^	
Ernie Bot 4.0 Turbo							
	Macroprecision	0.734 (0.018)		0.699-0.769		<.001^e^	
	Macrorecall	0.745 (0.018)		0.710-0.780		<.001^e^	
	Macro–*F*_1_-score	0.738 (0.018)		0.704-0.773		<.001^e^	
	Accuracy	0.749 (0.017)		0.716-0.782		<.001^e^	
Kimi							
	Macroprecision	0.755 (0.017)		0.723-0.788		<.001^e^	
	Macrorecall	0.764 (0.017)		0.732-0.797		<.001^e^	
	Macro–*F*_1_-score	0.758 (0.016)		0.725-0.790		<.001^e^	
	Accuracy	0.761 (0.016)		0.729-0.794		<.001^e^	
Zhipu Qingyan							
	Macroprecision	0.838 (0.015)		0.808-0.865		<.001^e^	
	Macrorecall	0.846 (0.014)		0.816-0.873		<.001^e^	
	Macro–*F*_1_-score	0.841 (0.014)		0.812-0.868		<.001^e^	
	Accuracy	0.846 (0.014)		0.818-0.873		<.001^e^	

^a^TCMPCSD-BERT: traditional Chinese medicine pancreatic cancer syndrome differentiation bidirectional encoder representations from transformers.

^b^Bootstrap 95% CIs.

^c^Compared with traditional Chinese medicine pancreatic cancer syndrome differentiation bidirectional encoder representations from transformers model.

^d^Not applicable.

^e^*P* values are obtained from the Welch *t* test.

### Comparison of Unstructured Long-Text Processing and Syndrome Differentiation Specificity for Pancreatic Cancer Between TCMPCSD-BERT and Web-Based Platforms

This study presents a preliminary comparison between TCMPCSD-BERT and online syndrome differentiation platforms in terms of unstructured long-text processing performance and the specificity of pancreatic cancer syndrome differentiation. As shown in Figure S2A (Multimedia Appendix 12), when we input a medical case diagnosed by a clinical expert as DH into the TCMPCSD-BERT model, it accurately predicted the case as DH with a confidence of 0.9994, closely matching the expert’s diagnosis. In contrast, as shown in Figure S2B (Multimedia Appendix 12), when entering the syndrome differentiation elements for pancreatic cancer with damp-heat accumulation, such as “腹部胀痛” (corresponding to the syndrome differentiation element in the guidelines: abdominal pain), “小便色黄” (yellow urine), “皮肤黄染” (yellowish skin), “舌红” (red tongue), “脉滑数” (rapid pulse) from the case in Figure S2A in Multimedia Appendix 12, the platform’s output is “邪热蕴蒸证” (evil-heat obstruction syndrome), rather than “湿热蕴结证” (DH). This result highlights that while the platform can perform basic syndrome differentiation, it fails to adequately recognize the specific pathological features of pancreatic cancer, resulting in a lack of specificity in its output. Furthermore, as shown in Figure S2C (Multimedia Appendix 12), when the original case’s long text was input, although the platform was able to process the text and provide a syndrome differentiation result, the output was “出血证” (bleeding syndrome), and key syndrome differentiation elements such as “腹部胀痛” (corresponding to the syndrome differentiation element in the guidelines: abdominal pain), “小便色黄” (yellow urine), “皮肤黄染” (yellowish skin), “舌红” (red tongue), “脉滑数” (rapid pulse) were not included in the “symptoms” (syndrome differentiation basis) sections. This indicates that the platform has limitations in processing unstructured long-text data and fails to effectively extract and recognize important syndrome differentiation features.

Additionally, in Figures S3 and S4 (Multimedia Appendix 12), we present comparison examples of TCMPCSD-BERT and the web-based platform in processing unstructured long texts and syndrome differentiation specificity for cases of spleen-deficiency syndrome and DH-SD. TCMPCSD-BERT also demonstrated stronger long-text processing performance and more accurate syndrome differentiation specificity in these pancreatic cancer syndrome tasks. Finally, in Figure S5 (Multimedia Appendix 12), we present the consultation interface of a certain 4-diagnostic instrument. Although the 4-diagnostic instrument supports basic syndrome differentiation operations, some devices still require users to manually select syndrome differentiation elements. This operational approach cannot directly process unstructured long-text clinical cases, resulting in relatively lower data processing efficiency and a more cumbersome operation process.

## Discussion

### Principal Findings

This study trained the TCMPCSD-BERT model using 6830 TCM medical records from pancreatic cancer cases. Compared to online syndrome differentiation platforms and the 4-diagnostic instrument, TCMPCSD-BERT demonstrates superior performance and efficiency in automatically identifying syndrome-related features from unstructured medical records, particularly in processing long-text, unstructured data. In comparison with LLMs guided by the prompt engineering framework, TCMPCSD-BERT shows significant advantages in terms of stability, accuracy, and operability. Furthermore, TCMPCSD-BERT is more capable of precisely identifying clinical features relevant to the syndrome differentiation of pancreatic cancer, showcasing improved specificity and customization, thus further confirming its potential for application in automated TCM diagnosis.

### Cultural and Conceptual Relevance of TCM Syndrome Patterns in Pancreatic Cancer

In TCM theory, DH and spleen-deficiency syndrome are considered 2 of the more commonly observed foundational patterns in patients with pancreatic cancer. Although these concepts have not been systematically validated within the framework of modern biomedical science, they have been applied in TCM clinical practice for many years and may offer an alternative perspective for understanding the general health status of patients with this disease.

Within the TCM theoretical system, the term “spleen” does not correspond precisely to the anatomical spleen as defined in modern medicine. Instead, it refers to a set of physiological functions related to the transformation of food, the regulation of fluids, and the generation of qi and blood [44]. It is believed that spleen function plays a vital role in maintaining internal homeostasis. Spleen-deficiency syndrome typically refers to a weakened state of the spleen and stomach functions, potentially resulting in impaired transformation and distribution of nutrients and subsequent systemic imbalances. Clinically, it is often associated with symptoms such as poor appetite, digestive difficulties, fatigue, and weakness [45-48]. Among patients with pancreatic cancer, particularly in the middle to advanced stages of disease progression, similar manifestations are frequently observed, including progressive weight loss, malnutrition, and reduced immune function [45]. While the underlying mechanisms remain to be fully elucidated, some clinical observations suggest that TCM interventions aimed at strengthening spleen function—such as treatments based on the principle of invigorating the spleen and replenishing qi—may lead to partial improvements in patient-reported symptoms and general condition, potentially enhancing tolerance to antitumor therapies [49]. These findings are still preliminary and require further high-quality studies for confirmation; however, they tentatively indicate that spleen-deficiency syndrome may represent a common clinical phenotype in patients with pancreatic cancer. Under the guidance of TCM’s holistic and pattern-differentiation principles, regulation of spleen function is viewed as a potentially supportive therapeutic approach that may contribute to improving patients’ functional status and mitigating treatment-related burdens.

DH, however, is often considered to be closely associated with dietary and lifestyle factors. TCM holds that excessive consumption of greasy, rich foods, alcohol, or unhygienic dietary practices may impair spleen and stomach function, leading to internal accumulation of dampness and stagnation of qi, which over time may transform into heat, giving rise to damp-heat pathology. For instance, alcohol is believed to be both “hot in nature and damp in substance,” and its overconsumption may exacerbate damp-heat accumulation. Similarly, rich foods are thought to contribute to the generation of dampness and heat [44]. In clinical practice, symptoms such as loss of appetite, abdominal distension, nausea, and jaundice—commonly observed in pancreatic cancer—are often interpreted within the TCM framework as indicative of DH [50]. Although systematic mechanistic studies on the relationship between DH and pancreatic cancer are still lacking, some clinical observations have indicated that patients with DH in advanced, unresectable pancreatic cancer may experience partial symptom relief—such as improvements in appetite, fatigue, and abdominal distension—when treated with a combination of chemotherapy and herbal formulas aimed at clearing heat and resolving dampness [51]. As such, this pattern is also regarded as relatively common among patients with this malignancy. It is worth noting that although some exploratory studies have begun to investigate the possible molecular, cellular, or microbiome-related underpinnings of these syndromes in the context of pancreatic cancer, the overall research in this area remains in an early stage, and the underlying biological mechanisms have yet to be clearly defined [47,48,52-55]. Against this backdrop, leveraging deep learning and other artificial intelligence techniques to conduct more objective and standardized phenotypic assessments of TCM syndromes in clinical populations may help advance syndrome research toward greater precision and empirical rigor. Such efforts may also facilitate the identification of potential biological correlates underlying TCM diagnostic categories, offering new avenues for integrative research.

### Feature Labeling in Expert Knowledge Learning for the TCMPCSD-BERT Model and Analysis of Study Results

In this study, TCMPCSD-BERT is designed to learn expert knowledge by identifying the key elements of syndrome differentiation in the training data. The model’s learning focus is influenced by several factors, including “knowledge framework construction,” “syndrome feature extraction,” “syndrome differentiation element weighting and symptom severity assessment,” and “syndrome differentiation thresholds setting.” A more detailed discussion on how these factors impact the focus of the TCMPCSD-BERT model’s learning can be found in Multimedia Appendix 13 (feature labeling in expert knowledge learning for the TCMPCSD-BERT model).

In comparative analyses with the 3 traditional baseline models, McNemar test indicated that TCMPCSD-BERT achieved statistically significantly higher classification accuracy across all 4 TCM syndrome types. Consistently, the mean values for macroprecision, macrorecall, macro–*F*_1_-score, and accuracy were greater for TCMPCSD-BERT in all comparisons, with its 95% bootstrap CIs showing no overlap with those of the comparator models. Welch *t* test further indicated that these differences were statistically significant in every comparison (*P*<.01). All pairwise comparisons exhibited evidence of heteroscedasticity, reflected in unequal variances between the distributions of performance metrics. In such contexts, Welch *t* test is generally considered appropriate, as it is designed to address heteroscedasticity by avoiding the pooling of variances and by adjusting the degrees of freedom, thereby helping to maintain control over type I error rates. On this basis, the statistical inferences from Welch *t* test remain interpretable even in the presence of unequal variances. In the present analysis, “variance” refers to the dispersion of metric distributions across the 5000 bootstrap samples for each model. Unequal variances may arise when models differ in the stability of their predictive performance—such as when 1 model shows minimal variation across resamples while another exhibits greater fluctuations. Such differences may be influenced by the randomness inherent in bootstrap resampling and by the class distribution of the test set, particularly under class imbalance. The presence of unequal variances does not, in itself, imply that the comparative results are unreliable. Rather, the persistence of statistically significant differences under the more conservative conditions of Welch *t* test may indicate that the observed differences are not solely attributable to variability in performance stability across models [56].

In the visualization of the self-attention mechanism of TCMPCSD-BERT, we observed that the model effectively identifies the relational structure between words within terms. For example, in Figure 4A, the terms “苔-薄-黄” (thin yellow coating) and “脉-弦” (wiry pulse), in Figure 4B, “纳-食” (appetite), “苔-白-腻” (white greasy coating), and “脉-细-弦” (thin wiry pulse), and in Figure 4C, “苔-白-厚” (thick white coating), represent combinations of words that are not typical in everyday language. However, TCMPCSD-BERT successfully “notices” the relationships between these words, as indicated by the thicker lines in the visualization, demonstrating the model’s ability to construct a vocabulary framework related to syndrome differentiation in pancreatic cancer and effectively identify the structural relationships of these specialized terms.

We used the integrated gradients algorithm to interpret the decision-making process of the TCMPCSD-BERT model. This algorithm assigns attribution scores to each word in the clinical text, with higher scores indicating greater importance of the term in syndrome differentiation. This helps us understand how the model processes and interprets key information in clinical texts. In Figures 5A-5C, we can see that the model assigns higher attribution scores to the diagnostic features corresponding to each syndrome type, emphasizing the critical role these symptoms play in the differentiation process. In Figure 5D, when we select a clinically diagnosed DH case and input the portion of the case that does not include syndrome differentiation features, the model assigns lower attribution scores to these irrelevant contents. This contrasts sharply with the higher scores assigned to the relevant diagnostic symptoms. These results demonstrate that the TCMPCSD-BERT model effectively identifies key diagnostic features across different syndrome types and assigns an appropriate weight to them. At the same time, the model effectively distinguishes irrelevant content from syndrome differentiation, minimizing its attention to such content.

Although the TCMPCSD-BERT model performed well on the test set, some misclassifications still occurred. To further understand the model’s error patterns, we selected misclassified cases from 3 typical syndrome types and conducted attribution analysis to explore the model’s shortcomings when handling complex symptoms. In 1 case where DH was misclassified as “spleen-deficiency syndrome” (Figure S1 in Multimedia Appendix 11), the attribution analysis showed that the model assigned lower attribution scores to several key symptoms of DH while assigning higher scores to symptoms associated with spleen-deficiency syndrome. Despite the case exhibiting multiple secondary symptoms related to spleen-deficiency syndrome, the absence of primary symptoms led to an incorrect diagnosis. In Figure S1B in Multimedia Appendix 11, the model misclassified a “spleen-deficiency syndrome” case as DH-SD. The model assigned lower attribution scores to several key symptoms of spleen-deficiency syndrome and higher scores to features associated with DH. Although the case displayed 1 primary symptom and 1 secondary symptom related to DH, it did not meet the diagnostic criteria for DH. In Figure S1C in Multimedia Appendix 11, the model misclassified a DH-SD case as “spleen-deficiency syndrome.” The attribution analysis showed that the model assigned lower attribution scores to key symptoms of DH (eg, “low-grade fever” and “abdominal pain”), which could be the cause of the misclassification. Furthermore, the model also assigned higher attribution scores to some clinically irrelevant features, such as “大便秘结” (constipation, 0.451) in Figure S1A in Multimedia Appendix 11, “小便调” (normal urination, 1.399) in Figure S1B in Multimedia Appendix 11, and “二便正常” (normal bowel and urination, 0.162). These misclassifications reflect the model’s insensitivity to less common symptoms (eg, low-grade fever) and its potential overreliance on secondary symptoms or confusion between subtle differences in syndrome features. While the model can capture some associations between syndrome features, it may fail to accurately distinguish the weighting of primary and secondary symptoms when faced with ambiguous boundaries between syndrome types, complex multisyndrome symptoms, or atypical symptom presentations, leading to misdiagnosis. Potential causes of misclassification may stem from the wide and sometimes vague definitions of syndromes and symptoms in TCM, which can blur the boundaries between different syndrome types. When the boundaries between syndromes are ambiguous, the model might not be able to differentiate between primary and secondary symptoms effectively. Additionally, the model’s reliance on secondary symptoms and its insensitivity to less common symptoms can contribute to misdiagnosis, highlighting the need for more precise and objective symptom definitions in future model improvements.

We compared the performance of the TCMPCSD-BERT model with that of English, Chinese, and TCM-specialized LLMs under the prompt engineering framework for syndrome differentiation tasks. McNemar test results showed that TCMPCSD-BERT generally demonstrated statistically superior classification accuracy across most TCM syndrome differentiation tasks. In particular, when compared with general-purpose LLMs (such as ChatGPT-4, Ernie Bot 4.0 Turbo, and Kimi), TCMPCSD-BERT exhibited significant advantages across all 4 syndrome categories (*P*<.01). However, when compared with more powerful or domain-specialized models (eg, ChatGPT-4o and the TCM-oriented model Zhipu Qingyan), the statistical advantage of TCMPCSD-BERT was not consistent across all tasks. For example, in the classification of “spleen-deficiency syndrome” and DH-SD, several comparisons yielded *P* values (eg, *P*=.072 and *P*=.083 for ChatGPT-4o; *P*=.059 and *P*=.053 for Zhipu Qingyan) that did not meet the conventional threshold for statistical significance. On the 1 hand, the intrinsic complexity of the 2 syndromes—characterized by overlapping or ambiguous symptom presentations—may increase the difficulty of accurate differentiation, thus posing challenges for all models. However, it is worth noting that spleen-deficiency syndrome (n=1185, 17.3%) and DH-SD (n=1178, 17.2%) represent the 2 syndrome types with the smallest number of cases in the test set. Compared to DH (n=1694) and other syndromes (n=2773), this imbalance in sample distribution may not only affect the sensitivity of statistical analyses but could also limit the TCMPCSD-BERT model’s ability to effectively learn syndrome-specific features during training. These factors may have contributed to the model’s relatively lower classification performance in these categories. Based on these observations, future studies should consider expanding the sample size for these less-represented syndrome types to enhance the model’s robustness and discriminatory capacity in imbalanced classification tasks.

In most cases, these LLMs were able to process the input clinical case content with reasonable accuracy. However, it is important to note that LLMs are sometimes affected by issues such as “large model hallucination” [18], “long-text forgetting” [19], and “instability,” which can result in incorrect outputs. In contrast, the TCMPCSD-BERT model demonstrated superior stability in the specific task of syndrome differentiation for pancreatic cancer. The model maintained high diagnostic accuracy when handling longer texts or complex symptoms and effectively mitigated the impact of the “forgetting” issue, ensuring reliable and consistent diagnoses. Furthermore, due to the inherent “long-text forgetting” issue in LLMs, it is often necessary to restart a new conversation and re-enter the prompt framework after 10 to 20 queries. This process makes the use of LLMs cumbersome in practical applications, limiting their ability to provide continuous and stable service. As a result, the TCMPCSD-BERT model exhibited greater stability, accuracy, and operational efficiency in the specific task of pancreatic cancer syndrome differentiation compared to LLMs guided by the prompt engineering framework.

In this study, we conducted a preliminary comparison between the TCMPCSD-BERT model, online syndrome differentiation platforms, and certain 4-diagnostic instruments in terms of unstructured long-text processing performance and specificity in pancreatic cancer syndrome differentiation. In TCM clinical practice, different diseases have their own diagnostic characteristics, meaning that the same symptoms may have significantly different implications for syndrome differentiation depending on the disease. For instance, when inputting the syndrome differentiation elements for pancreatic cancer “damp-heat accumulation syndrome,” the platform may mistakenly identify it as a different disease, such as a hepatitis-related syndrome, and fail to assess the pancreatic cancer syndrome content accurately. Moreover, web-based platforms have not yet been able to effectively process long, unstructured clinical case texts. While they can perform basic syndrome differentiation tasks, they have significant limitations in identifying the specific pathological features of pancreatic cancer, leading to a lack of diagnostic specificity in their outputs. These platforms and 4-diagnostic instruments often require users to manually select syndrome differentiation elements, making them cumbersome when handling unstructured long texts and resulting in lower data processing efficiency. The root cause of this issue may lie in the limitations of computational power and memory in web-based platforms and 4-diagnostic instruments, which typically use lightweight models that are not well-suited for handling unstructured long-text data [57,58]. In contrast, the TCMPCSD-BERT model performs more accurately and efficiently in this task. Furthermore, by building on the TCMPCSD-BERT model and incorporating more powerful models, such as vision transformer, we can create a more efficient and precise syndrome quantification framework. This scalability and potential remain unmatched by current web-based platforms and hardware-based syndrome differentiation tools.

### Limitations and Future Directions

Compared to previous NLP studies on TCM syndrome differentiation [14-17], this study demonstrates several key advantages. First, it trained the TCMPCSD-BERT model on a larger dataset comprising 6830 clinical cases, which enhances its generalization ability in real-world applications and allows it to effectively handle the uncertainty and complexity of actual clinical scenarios. Additionally, this study conducted a more comprehensive evaluation and analysis of the TCMPCSD-BERT model, including self-attention visualization and integrated gradient analysis, revealing its alignment with clinical standards. Furthermore, through the analysis of misclassified cases, this study explored the model’s limitations in handling complex symptoms. Moreover, this research also performed an initial evaluation of several LLMs with English and Chinese backgrounds, as well as TCM-specific LLMs, assessing their performance in TCM syndrome differentiation tasks for pancreatic cancer under the guidance of the prompt engineering framework, and compared them with TCMPCSD-BERT. The results indicated that, especially in research contexts and specific problem evaluations, customized models currently retain irreplaceable advantages in terms of precision and stability. Additionally, several studies support this view, demonstrating that for specific tasks, small- to medium-scale customized models can achieve and, in some cases, surpass the performance of large-scale models with relatively fewer data requirements [31,59-61].

However, this study does have several limitations. Foremost, it is important to acknowledge that the definitions of “syndrome types” and “syndrome elements” in this study remain relatively subjective and broad. While existing literature has demonstrated a close association between DH and inflammatory microenvironments, as well as a strong link between spleen-deficiency syndrome and energy metabolism, syndrome differentiation continues to predominantly rely on the observation of clinical symptoms and signs. Currently, effective clinical diagnostic indicators to guide syndrome differentiation are still in development. In light of these limitations, there are several potential solutions and improvements for future research. First, it is important to address the broad nature and potential overlap of many syndrome elements in the current differentiation process. We believe that future studies should incorporate the insights of more authoritative experts across various fields to refine and clarify the definitions of the symptoms and signs associated with each syndrome type. Furthermore, incorporating a graded approach, such as differentiating symptoms into mild, moderate, and severe categories, or developing a concise scoring system, would improve the precision of syndrome differentiation. This stratification can help mitigate the broadness of the syndrome elements and reduce overlap, ultimately leading to more accurate and distinct syndrome definitions. Second, this study used independent evaluations by 2 experts and quantified the consistency of their annotations using Cohen κ coefficient, which yielded a value of 0.913, indicating a high level of agreement between the 2 experts in syndrome differentiation. However, only a single round of annotation was conducted. Future research could benefit from implementing multiple rounds of evaluation, which would provide a more robust and reliable means of mitigating subjective biases. By incorporating iterative assessments, we may achieve even more precise evaluations of syndrome characteristics, further enhancing the reliability of the differentiation process. The syndrome differentiation model developed in this study, based on the BERT architecture, aims to assist in automating the identification of syndrome-related information embedded within patient case data. By integrating the BERT architecture with image processing models, a more objective and standardized approach to quantifying syndrome information can be achieved. This method will not only significantly enhance the accuracy of syndrome differentiation but also provide an important tool for subsequent research, assisting in the identification of clinical diagnostic biomarkers for pancreatic cancer and promoting the objectification and precision of TCM syndrome differentiation.

Furthermore, the BERT model requires a substantial amount of data for training. The 6830 clinical cases used in this study are relatively limited for a 4-category classification task, and as the number of classification labels increases, the data requirements grow significantly. As shown in the “Dataset and Model Training Details” section and Table 1, the number of cases corresponding to the 4 syndrome labels is not balanced, and there are noticeable differences in baseline characteristics—such as gender distribution, tumor staging, and treatment plans—across these syndrome categories. Additionally, data collection should ensure the adequate representation and comprehensiveness of symptoms to reflect expert diagnostic thought processes and avoid biases in the model’s learning and judgment. The current dataset only includes a subset of common clinical symptoms and signs, leaving many syndrome-related symptoms insufficiently learned. Additionally, to ensure adequate representation of each label and avoid potential modeling bias due to severe class imbalance, this study included only syndrome types with at least 500 corresponding cases as classification targets. Cases with unclear syndrome manifestations or those belonging to underrepresented categories were collectively assigned to the “others” group. While this strategy was intended to improve the model’s training stability and reduce the risk of overfitting to poorly represented classes, it may inevitably constrain the model’s ability to learn and recognize rarer but clinically relevant syndrome patterns. This limitation could affect the model’s adaptability and generalizability in more diverse or nuanced clinical contexts. Furthermore, the 4 syndrome categories adopted in this study exhibit certain limitations in diagnostic breadth and are insufficient to encompass the complex and diverse syndrome patterns and reasoning processes involved in TCM clinical practice for pancreatic cancer. To address these limitations, future work will aim to incorporate a larger volume of high-quality clinical records and explore transfer learning strategies to extend the diagnostic breadth of the TCMPCSD-BERT model and enhance its potential applicability in real-world clinical scenarios.

Moreover, another important limitation of this study is that all training, validation, and testing data were obtained from a single institution—Fudan University Shanghai Cancer Center. Although we used internal 10-fold cross-validation and withheld a portion of the data as an independent test set, this internal evaluation may not fully capture the model’s generalizability to external clinical settings. Given that syndrome-related language expressions, documentation habits, and diagnostic styles can vary across institutions and regions, center-specific data patterns may lead to potential overfitting or bias, thus limiting the external applicability of the TCMPCSD-BERT model. Unfortunately, due to institutional restrictions and data privacy regulations, we were unable to obtain external datasets from other hospitals for cross-institutional validation in this study. Acknowledging this limitation, we plan to expand our collaboration network and actively seek access to deidentified data from additional centers in future work. Such efforts will facilitate a more comprehensive evaluation of model robustness across diverse clinical environments and further enhance the reliability and applicability of the proposed framework.

### Conclusions

The TCMPCSD-BERT model demonstrates potential for identifying syndrome-related elements from unstructured clinical case texts and supporting automated, scalable syndrome differentiation in pancreatic cancer. Based on the findings of this study, it appears to offer improved operability over 4-diagnostic instruments and web-based platforms, as well as greater stability and task-specific accuracy than LLMs. Nonetheless, these results should be interpreted with caution due to the subjectivity of syndrome definitions, data imbalance, and reliance on preprocessed and expert-annotated inputs. Future research involving larger and more representative populations is needed to further validate the model and explore its potential for broader application in real-world clinical settings.

## Appendix

Multimedia Appendix 1 Workflow of feature extraction, expert annotation, and data preprocessing.

Multimedia Appendix 2 TCMPCSD-BERT model architecture and training strategy. TCMPCSD-BERT: traditional Chinese medicine pancreatic cancer syndrome differentiation bidirectional encoder representations from transformers.

Multimedia Appendix 3 Chinese-English comparison for Figure 1.

Multimedia Appendix 4 Evaluation metrics, McNemar test, and stratified bootstrap statistical analysis for multi-class syndrome differentiation.

Multimedia Appendix 5 Explanation of syndrome differentiation decisions.

Multimedia Appendix 6 LLMs prompt engineering. LLM: large language model.

Multimedia Appendix 7 The Prompt Framework for LLMs in English and Chinese. LLM: large language model.

Multimedia Appendix 8 Interrater agreement and optimal model parameters.

Multimedia Appendix 9 Mapping clinical features to TCM guideline terms for syndrome differentiation. TCM: traditional Chinese medicine.

Multimedia Appendix 10 Attribution analysis of diagnostic features in TCM syndromes. TCM: traditional Chinese medicine.

Multimedia Appendix 11 Attribution analysis of misclassified cases.

Multimedia Appendix 12 Comparison of TCMPCSD-BERT, web-based platforms, and diagnostic instruments in pancreatic cancer syndrome differentiation. TCMPCSD-BERT: traditional Chinese medicine pancreatic cancer syndrome differentiation bidirectional encoder representations from transformers.

Multimedia Appendix 13 Feature labeling in expert knowledge learning for the TCMPCSD-BERT model. TCMPCSD-BERT: traditional Chinese medicine pancreatic cancer syndrome differentiation bidirectional encoder representations from transformers.

## Abbreviations

BERT: bidirectional encoder representations from transformers
DH: damp-heat syndrome
DH-SD: damp-heat with spleen-deficiency syndrome
LLM: large language model
LSTM: long short-term memory
NLP: natural language processing
TCM: traditional Chinese medicine
TCMPCSD-BERT: traditional Chinese medicine pancreatic cancer syndrome differentiation bidirectional encoder representations from transformers
Text-CNN: text convolutional neural network
XGBoost: extreme gradient boosting

## Data Availability

The source code underlying this paper is available on GitHub [62]. The datasets generated and analyzed during this study are not publicly available due to patient privacy considerations. However, deidentified data may be made available from the corresponding author upon reasonable request and with approval from the institutional ethics committee.
