# Supplementary material for: Identification of Syndrome Types in Patients With Pancreatic Cancer From Free Text in Electronic Medical Records: Model Development and Validation
Source: JMIR Form Res. 2025 Oct 3;9:e70602. doi: 10.2196/70602 (PMC12534766; doi:10.2196/70602)
Supplement: Multimedia Appendix 1 [file formative_v9i1e70602_app1.docx]

## Feature Engineering and Data Preprocessing

### Feature Engineering

The TCM syndrome differentiation standards used in this study were based on *Integrated Chinese and Western Medicine in Oncology*, edited by Wei Hou[1], and *Principles of Syndrome Differentiation and Treatment in TCM*, edited by Zhaoqin Fang[2]. By combining these references with the specific patient data collected, we included two primary syndromes (damp-heat syndrome and spleen-deficiency syndrome) and one combined syndrome (damp-heat with spleen-deficiency syndrome). The specific diagnostic elements and criteria for each syndrome type are detailed in Supplementary Table 1.

**Supplementary Table 1. Diagnostic elements of damp-heat syndrome and spleen-deficiency syndrome, and criteria for determining the four syndrome type labels.**

| **Category** | **Damp-Heat Syndrome** | **Spleen-Deficiency Syndrome** |
| --- | --- | --- |
| **Primary Symptoms** | Yellowish skin; yellow sclera; yellow urine; gray-white stool; loss of appetite; nausea; vomiting; abdominal bloating; abdominal pain. | Fatigue; weakness; shortness of breath; dull lower back pain; fullness in chest and abdomen, aggravated after meals; loose stools; indigestion. |
| **Primary Tongue Indicators** | Yellow coating; yellow greasy coating. | Pale and swollen tongue. |
| **Primary Pulse Indicators** | Wiry, rapid pulse. | Deficient pulse. |
| **Secondary Symptoms** | Persistent low-grade fever; thirst without strong desire to drink; bitter taste; foul breath; foul-smelling loose stools; restlessness. | Small appetite; emaciation; shortness of breath; spontaneous sweating; aversion to cold; cold limbs; discomfort or pain in the upper abdomen relieved by pressure; facial puffiness; pale complexion; aversion to wind; dry mouth with little desire to drink; nausea; vomiting; edema in lower limbs; ascites. |
| **Additional Tongue Indicators** | Red tongue. | Teeth marks on the tongue edges; white slippery coating; thin white coating; pale tongue; thin coating; thin greasy coating. |
| **Additional Pulse Indicators** | Rapid pulse. | Deep and thin pulse; thin and weak pulse; deep and slow pulse; thin pulse; thin wiry pulse; soft and moderate pulse. |

### Syndrome Differentiation Criteria

The syndrome is identified if two primary symptoms are present along with any primary tongue or pulse indicator. It is also identified if two primary symptoms and one secondary symptom are present, along with any tongue or pulse indicator related to this syndrome. Additionally, the syndrome can be identified if one primary symptom and at least two secondary symptoms are present, along with any related tongue or pulse indicator. Damp-Heat with Spleen-Deficiency Syndrome is defined by clinical features that simultaneously meet the criteria for both damp-heat and spleen-deficiency syndromes. Cases with fewer than 500 records for a single syndrome type, or clinical presentations lacking specific syndrome differentiation characteristics, were collectively categorized as “Others.”

### Removal of Irrelevant Text

Most clinical records in this study included documentation from multiple patient visits, often spanning extended periods. However, symptoms that had previously resolved—particularly those noted to have improved substantially following treatment—no longer contributed meaningful diagnostic value for current syndrome differentiation. In accordance with TCM diagnostic principles, syndrome classification is primarily based on the patient's present signs and symptoms (ke xia zheng), rather than historical complaints that have faded or disappeared. Moreover, retaining such outdated content could introduce noise and interfere with the model’s ability to accurately learn the relevant syndrome-related features.

To address this issue, we implemented a clinically informed filtering process, as illustrated in section 1.1 of Figure 1. Each record was reviewed by experienced TCM physicians, who excluded irrelevant historical or background content and retained only current symptoms and historically significant information deemed essential for accurate syndrome identification. This expert-guided selection approach is also aligned with common practices in TCM randomized controlled trials (RCTs), in which syndrome classification is typically based on the patient’s active presentation at the time of assessment[3,4].

Additionally, we standardized the length of each input record to 150–500 words. This constraint served two purposes: first, it ensured the retention of essential diagnostic context while minimizing redundant or non-informative information; second, it aligned with the technical input limitation of the BERT model, which accepts a maximum of 512 tokens[5]. Input records exceeding this threshold risk being truncated or fragmented, potentially compromising semantic coherence and model performance. While recent transformer variants allow longer inputs, they generally require significantly larger training datasets to be effective, which exceeded the scope of the current study[6,7].

This clinically grounded and technically optimized preprocessing procedure helped ensure that the model could efficiently focus on the core diagnostic signals most relevant to syndrome differentiation in a real-world, low-data context.

### Extraction of Syndrome Differentiation Features and Assignment of Syndrome Labels

Symptoms and signs in TCM clinical case records are typically documented in unstructured text format. While many clinical expressions are relatively straightforward—such as “yellowish skin,” “yellow urine,” and “loose stools”—and can be directly mapped to the diagnostic elements defined in syndrome differentiation guidelines, some clinically relevant information may be conveyed in more subtle, diverse, or implicit linguistic forms within unstructured clinical narratives.

For example, in the case of the diagnostic element Fatigue associated with Spleen-Deficiency Syndrome, a patient who is a manual laborer may describe their symptom as: “I used to be fine at work, but lately I have to sit down and rest after just a short while.” Though “fatigue” is not explicitly stated, the description implies a decline in physical endurance and energy. Similarly, the diagnostic element foul-smelling loose stools associated with Damp-Heat Syndrome may appear in the clinical record as: “Recently, my stool has been loose and smells worse than usual. Even after finishing, I still feel like I haven’t fully emptied my bowels.” This type of expression reflects multiple diagnostic elements—loose stools, foul smell, and tenesmus-like sensation—all conveyed indirectly. Another example is persistent low-grade fever, also associated with Damp-Heat Syndrome, which may be described as: “Recently, I’ve had a mild feverish sensation in the afternoons or after dinner, especially on my face or body.” Although the patient may not report a measured fever, the contextual description indicates the presence of a subjective low-grade fever.

Such challenges are not unique to TCM; similar issues are commonly encountered in modern clinical NLP tasks, which frequently rely on patient-reported complaints (subjective descriptions) or clinician-documented impressions and observations as primary input sources. Given that the vast majority of real-world medical records remain in unstructured form, a substantial portion of diagnostic information must still be extracted through linguistic cues and clinical interpretation[8]. Therefore, reliance on expert annotation remains a common and realistic limitation in the development of language-driven clinical decision models, both in TCM and Western medicine.

To address this, in the present study, each clinical case record was first reviewed to remove irrelevant content (see Section 1.2 of Figure 1), and then independently reviewed by two experienced TCM physicians, each with over 10 years of clinical practice. These physicians extracted syndrome-relevant features in accordance with standard differentiation criteria and converted them into structured representations (see Section 1.3 of Figure 1). Subsequently, each physician independently annotated syndrome elements and assigned syndrome labels to each case based on the same differentiation standards (see Section 1.4 of Figure 1). To ensure the consistency and reliability of annotations, the two physicians compared their results across all records. In cases of disagreement, detailed comments were recorded and referred to a senior TCM expert for adjudication until a final consensus was reached.

### Data Preprocessing

The annotated text data were first cleaned to remove non-text elements, redundant punctuation, and other irrelevant information. Since this study involves a multi-class classification task, the cleaned text was converted into a one-hot encoding format to meet the requirements for multi-class processing (as shown in section 1.5 of Figure 1). Next, the text was tokenized using the WordPiece tokenizer from the pre-trained BERT model, breaking down sentences into tokens (T). Special markers [CLS] were added at the beginning of the entire input text and [SEP] at the end of each segment, ensuring that the BERT model could accurately interpret the structure and contextual relationships within the text.

### Application of Cohen’s Kappa Coefficient in Evaluating Inter-Annotator Agreement

The Cohen’s kappa coefficient is a statistical measure used to evaluate the degree of agreement between two annotators in classification tasks. Unlike the simple percent agreement, the Cohen’s kappa coefficient accounts for agreement that may occur by chance, thereby providing a more objective assessment of the true level of agreement between raters. In this study, which involves a four-class classification task, the computation of Cohen’s kappa is based on a contingency table constructed from the annotation results of two experts. The rows represent the annotations of Expert A, and the columns represent the annotations of Expert B, as shown below:

| Expert A \ Expert B | damp-heat syndrome (1) | spleen-deficiency syndrome (2) | damp-heat with spleen-deficiency syndrome (3) | Others (4) | Row total |
| --- | --- | --- | --- | --- | --- |
| damp-heat syndrome (1) | $a_{11}$ | $a_{12}$ | $a_{13}$ | $a_{14}$ | $R_{1}$ |
| spleen-deficiency syndrome (2) | $a_{21}$ | $a_{22}$ | $a_{23}$ | $a_{24}$ | $R_{2}$ |
| damp-heat with spleen-deficiency syndrome (3) | $a_{31}$ | $a_{32}$ | $a_{33}$ | $a_{34}$ | $R_{3}$ |
| Others (4) | $a_{41}$ | $a_{42}$ | $a_{43}$ | $a_{44}$ | $R_{4}$ |
| Column total | $C_{1}$ | $C_{2}$ | $C_{3}$ | $C_{4}$ | $N$ |

Where:

- $a_{ij}$ denotes the number of cases annotated as category $i$ by Expert A and as category $j$ by Expert B;
- $R_{i}$ is the total number of cases that Expert A annotated as category $i$ (row total);
- $C_{j}$ is the total number of cases that Expert B annotated as category $j$ (column total);
- $N$ is the total number of cases.

Observed agreement ($p_{0}$) is the proportion of cases for which both experts assigned the same label:

$$p_{0}=\frac{\sum_{i=1}^{4} a_{ii}}{N}$$

Expected agreement ($p_{e}$) is the sum of the probabilities that both experts would randomly agree on each category:

$$p_{e}=\sum_{i=1}^{4} \left( \frac{R_{i}}{N}\times\frac{C_{i}}{N} \right)$$

The Cohen’s kappa coefficient ($\kappa$) is then calculated as:

$$\kappa=\frac{p_{0}-p_{e}}{1-p_{e}}$$

According to the empirical benchmark proposed by Landis and Koch (1977)[9], the Cohen’s Kappa coefficient can be interpreted as follows:

- 0.00–0.20: slight agreement;
- 0.21–0.40: fair agreement;
- 0.41–0.60: moderate agreement;
- 0.61–0.80: substantial agreement;
- 0.81–1.00: almost perfect agreement.

## Reference

1. Hou W. *Integrated Chinese and Western Medicine in Oncology*. Beijing: People's Medical Publishing House; 2022.978-7-117-34044-1: 978-7-117-34044-1.

2. Fang Z. *Principles of Syndrome Differentiation and Treatment in TCM*. Shanghai: Shanghai University of Traditional Chinese Medicine Press; 2008.7-81121-035-5: 7-81121-035-5.

3. Lin L, Xiao J, Wu L, et al. Efficacy and safety of Kegan Liyan oral liquid for patients with acute pharyngitis: A randomized, double-blinded, placebo-controlled, multi-center trial. Phytomedicine. Nov 2024;134:155960. doi:10.1016/j.phymed.2024.155960

4. Wen T, Liu X, Pang T, et al. The Efficacy of Chaihu-Guizhi-Ganjiang Decoction on Chronic Non-Atrophic Gastritis with Gallbladder Heat and Spleen Cold Syndrome and Its Metabolomic Analysis: An Observational Controlled Before-After Clinical Trial. Drug Des Devel Ther. 2024;18:881-897. doi:10.2147/dddt.S446336

5. Cui Y, Che W, Liu T, Qin B, Yang Z. Pre-Training With Whole Word Masking for Chinese BERT. IEEE/ACM Transactions on Audio, Speech, and Language Processing. 2019;29:3504-3514.

6. Han X, Zhang Z, Ding N, et al. Pre-Trained Models: Past, Present and Future. ArXiv. 2021;abs/2106.07139

7. Dai Z, Yang Z, Yang Y, Carbonell JG, Le QV, Salakhutdinov R. Transformer-XL: Attentive Language Models beyond a Fixed-Length Context. ArXiv. 2019;abs/1901.02860

8. Au Yeung J, Shek A, Searle T, et al. Natural language processing data services for healthcare providers. BMC Med Inform Decis Mak. Nov 26 2024;24(1):356. doi:10.1186/s12911-024-02713-x

9. Landis JR, Koch GG. The measurement of observer agreement for categorical data. Biometrics. Mar 1977;33(1):159-74.
