# Supplementary material for: Identification of Syndrome Types in Patients With Pancreatic Cancer From Free Text in Electronic Medical Records: Model Development and Validation
Source: JMIR Form Res. 2025 Oct 3;9:e70602. doi: 10.2196/70602 (PMC12534766; doi:10.2196/70602)
Supplement: Multimedia Appendix 2 [file formative_v9i1e70602_app2.docx]

### Input Embedding

The input embedding primarily consists of token embeddings ($E_{token}$), segment embeddings ($E_{segment}$), and position embeddings ($E_{position}$). These three embedding vectors collectively form the input to the BERT model, ensuring the model can capture the semantic information of tokens, contextual order, and sentence structure.

### Multi-head Attention

The self-attention mechanism within the Transformer architecture is essential to the BERT model’s capability for understanding syndrome differentiation terminology and context, supporting both MLM and multi-class classification tasks, and enabling a comprehensive understanding of TCM syndrome differentiation features. Once input embeddings are processed, each embedding vector $E_{i}$ is passed into the Transformer for further processing. Initially, three distinct weight matrices, $W_{Q}$, $W_{k}$, and $W_{V}$, are applied to generate the query vector $Q_{i}$, key vector $K_{i}$, and value vector $V_{i}$, represented as follows:

$$Q_{i}=W_{Q}E_{i}, K_{i}=W_{K}E_{i}, V_{i}=W_{V}E_{i}$$

In this setup, $Q_{i}$ represents the token currently being processed, while $K_{i}$ represents the remaining tokens within the context. The model evaluates the relevance between the current token and other tokens by calculating the dot product between $Q_{i}$ and all tokens in the input text sequence $(K_{i})$, represented as:

$$Attention\left( Q, K, V \right)=softmax\left( \frac{QK^{T}}{\sqrt{d_{k}}} \right)V$$

This equation quantifies the association (attention weights) between tokens, applying these weights to $V$ to produce a final representation that integrates semantic, positional, and contextual information. This mechanism enables the model to capture complex, long-range dependencies, a core strength of the Transformer architecture. The resultant representation is integrated through self-attention to form the hidden state $H$:

$$H_{i}=Attention\left( Q_{i}, K_{i}, V_{i} \right)+E_{i}$$

With each iteration, $H$ is updated. By passing the hidden representation from the previous layer $H^{\left( n-1 \right)}$ into the current layer, the model generates a new hidden representation $H^{(n)}$：

$$H^{(n)}=Attention(H^{\left( n-1 \right)})$$

The self-attention mechanism equips the model with the ability to interpret context and capture intricate semantic relationships, making it particularly effective for clinical records that are rich in symptoms and complex semantic associations. Building upon this framework, the MLM task further enhances the model’s understanding of TCM-related semantics and contextual nuances.

### Masked Language Model (MLM) Task

The MLM task improves the model’s contextual understanding by randomly masking specific tokens and prompting the model to predict their content. In each iteration, a subset of tokens $H$ is randomly masked, resulting in $H_{mask}$:

$$H_{mask}^{(t)}=Mask(H^{\left( t \right)})$$

Tokens that remain unmasked proceed to generate $Q$, $K$, and $V$ and are processed through the self-attention mechanism, providing the model with contextual information. Using this context, the model then predicts the content of the masked tokens:

$$P\left( word \right| context)=softmax(W_{h}h_{mask}+b)$$

In this process, a cross-entropy loss function quantifies the difference between the model’s predicted probability distribution and the actual tokens:

$$\mathcal{L}=-\sum_{i} y_{i}\log(p_{i})$$

Where $y_{i}$ represents the true labels, and $p_{i}$ is the predicted probability. Through gradient backpropagation, the model iteratively adjusts its parameters in response to the loss value until it can accurately predict the masked tokens. The MLM task compels the model not only to consider direct token relationships but also to grasp the broader context within sentences and paragraphs through the self-attention mechanism. Over the course of training, the model continuously optimizes its parameters, gradually refining the representations of $E_{token}$ and $E_{segment}$, thereby enhancing its ability to understand the semantics and contextual relationships within TCM clinical texts.

### Design of the Multi-Class Classification Output Layer

Following fine-tuning through the MLM task, the BERT model develops a more nuanced understanding of the semantics and contextual information within TCM clinical records. We then modify the model’s output layer to better capture the relationship between clinical record content and syndrome labels. When a TCM clinical record is input into the model, the text is first processed through input embedding and the self-attention mechanism, generating a hidden state $H$. At this stage, $H$ effectively encapsulates the semantic and contextual information within the clinical record. The hidden state of the [CLS] token, $H_{CLS}^{(N)}$, is utilized as a global representation of the entire input sequence. This embedding aggregates the main semantic features of the input text and is represented as a fixed-length feature vector:

$$Z_{Global}=H_{CLS}^{(N)}$$

The feature vector, $Z_{global}$, is then fed into a fully connected layer, where the softmax activation function computes the prediction probability $p_{i}$ for each syndrome type (indicating the model’s predicted probability for the $i$*-* th syndrome type):

$$p_{i}=softmax(WZ_{Global}+b)$$

For the multi-class classification task, a binary cross-entropy loss function is employed to quantify the difference between the model’s predicted probability distribution and the true syndrome labels:

$$L=-\sum_{i=1}^{N} [y_{i}\log\left( p_{i} \right)+\left( 1-y_{i} \right)\log\left( 1-p_{i} \right)]$$

Where $y_{i}$ represents the true label. Through gradient backpropagation, the model iteratively adjusts its parameters based on the loss value, progressively improving its accuracy in syndrome label prediction. Throughout this process, the model learns not only the semantic connections between words but also the complex associations between clinical record content and syndrome labels. Ultimately, the model becomes proficient in interpreting clinical records and accurately predicting syndrome labels, and we designate this trained model as TCMPCSD-BERT.
