# Supplementary material for: Identification of Syndrome Types in Patients With Pancreatic Cancer From Free Text in Electronic Medical Records: Model Development and Validation
Source: JMIR Form Res. 2025 Oct 3;9:e70602. doi: 10.2196/70602 (PMC12534766; doi:10.2196/70602)
Supplement: Multimedia Appendix 3 [file formative_v9i1e70602_app3.docx]

**Supplementary Table 2. Chinese-English comparison for Figure 1.**

| **Location in Figure 1** | **English Translation** | **Original Text in Chinese** |
| --- | --- | --- |
| **1.1 Removal of Irrelevant Text** | On March 11, 2019, the patient underwent TACE. Gemcitabine (1.0 g) was infused into the celiac trunk via microcatheter, followed by cisplatin (40 mg) mixed with 3 mL of ultra-liquid iodized oil into the proper hepatic artery. The procedure was successful, and the patient recovered well. The patient was admitted for further treatment. Currently, the patient reports abdominal pain, fatigue, weakness, adequate sleep, poor appetite, yellowish urine, and normal bowel movements, with a 6 kg weight loss over the past two months. Physical examination revealed a KPS score of 80. The patient was conscious and breathing steadily, with moderate jaundice in the skin and sclera. No superficial lymphadenopathy was detected. Shifting dullness was positive (+), and moderate edema was noted in both lower extremities. The patient's tongue was red with a thin yellow coating, and the pulse was wiry. | 于2019.03.11行TACE，于腹腔干注入吉西他滨1.0，微导管跟进，超选于肝固有动脉注入顺铂 40mg及超液化碘油3ml，过程顺利，术后恢复可。现为进一步治疗我院门诊收治入院。目前患者诉腹痛，神疲乏力，睡眠可，食欲欠佳，小便色黄，大便正常,体重近2月下降6kg。体格检查：KPS 80。神志清楚，呼吸平稳，皮肤巩膜中度黄染，浅表淋巴结未触及肿大；移动性浊音(+)。双下肢中度水肿。患者舌红，苔薄黄，脉弦。 |
| **1.2 Syndrome Differentiation Feature Extraction** | Currently, the patient reports abdominal pain, fatigue, weakness, adequate sleep, poor appetite, yellowish urine, and normal bowel movements, with a 6 kg weight loss over the past two months. Physical examination revealed a KPS score of 80. The patient was conscious and breathing steadily, with moderate jaundice in the skin and sclera. No superficial lymphadenopathy was detected. Shifting dullness was positive (+), and moderate edema was noted in both lower extremities. The patient's tongue was red with a thin yellow coating, and the pulse was wiry. | 目前患者诉腹痛，神疲乏力，睡眠可，食欲欠佳，小便色黄，大便正常,体重近2月下降6kg。体格检查：KPS 80。神志清楚，呼吸平稳，皮肤巩膜中度黄染，浅表淋巴结未触及肿大；移动性浊音 (+)。双下肢中度水肿。患者舌红，苔薄黄，脉弦。 |
| **1.3 Mapping to Guidelines** | abdominal pain | 腹痛 |
|  | fatigue | 神疲 |
|  | weakness | 乏力 |
|  | loss of appetite | 食欲不振 |
|  | small appetite | 食少 |
|  | yellow urine | 小便黄 |
|  | emaciation | 消瘦 |
|  | yellowish skin | 皮肤黄染 |
|  | yellow sclera | 巩膜黄染 |
|  | ascites | 腹水 |
|  | edema in lower limbs | 下肢浮肿 |
|  | red tongue | 舌红 |
|  | yellow coating | 苔黄 |
|  | wiry, rapid pulse | 脉弦数 |
|  | thin wiry pulse | 脉弦细 |
| **1.4 Syndrome Label Assignment** | Damp-Heat with Spleen-Deficiency Syndrome | 湿热蕴结兼脾虚气滞证 |
|  | Damp-Heat Syndrome | 湿热蕴结证 |
|  | Others | 其他 |
|  | Spleen-Deficiency Syndrome | 脾虚气滞证 |
| **1.4.1 Labeled TCM^a^ Clinical Records (Example 1)** | (1) A 58-year-old male patient. (2) The patient was admitted for further treatment of "pancreatic cancer with pelvic metastasis for 2 months, one month after interventional chemotherapy." (3) Presenting Symptoms at Admission: The patient reported lower abdominal pain, poor appetite, yellow-colored urine with normal volume, and constipation. No fever or jaundice was observed. He reported a weight loss of 3 kilograms since discharge. (4) Physical Examination: The Karnofsky Performance Status (KPS) was 90. The patient was alert and lucid, with no significantly enlarged supraclavicular lymph nodes palpable. The abdomen was flat and soft, with tenderness in the lower abdomen (+) but no rebound tenderness (-). The liver, subxiphoid, and spleen were not palpable below the costal margin. Bowel sounds were normal, without hyperactivity, and shifting dullness was positive (+). An old surgical scar approximately 50 cm long was observed on the anterior aspect of the right thigh, which had healed well. No pitting edema was noted in the lower extremities. The patient’s tongue was red with a yellow greasy coating, and the pulse was wiry. | （1）患者男，58岁。（2）因“胰腺癌盆腔转移2月，介入化疗后1月余”入院。为进一步治疗再次入院。（3）入院时：下腹部痛，纳差，小便色黄，量正常，大便不畅，无发热，无黄疸，出院至今消瘦3公斤。（4）体格检查：KPS 90,神清，锁骨上淋巴结未扪及明显增大。腹平软，下腹部压痛（+），反跳痛（-），肝肋下未及，剑突下未及，脾肋下未及。肠鸣音无亢，移动性浊音（+）。右下肢大腿正面可见一陈旧性手术疤痕，长约50cm,愈合良好，双下肢无凹陷性水肿。患者舌红，苔黄腻，脉弦。 |
| **1.4.2 Labeled TCM Clinical Records (Example 2)** | Male, 58 years old. Hospitalized for "persistent mild lower abdominal pain accompanied by weight loss for over 3 months and a pancreatic tail mass discovered half a month ago." Admitted to the department for further diagnosis and treatment. The patient reported mild lower abdominal pain, poor appetite, and slightly dry stools for over 2 months, which were relieved with the use of glycerin enemas. He denied a history of constipation. Urine was yellow, and there was no significant weight loss over the past 3 months. The patient denied nausea, vomiting, or fever. Physical Examination: Karnofsky Performance Status (KPS): 90. The patient was alert and lucid. No significantly enlarged supraclavicular lymph nodes were palpable. The abdomen was flat and soft, with no tenderness or rebound tenderness. The liver, subxiphoid, and spleen were not palpable below the costal margin. Bowel sounds were normal without hyperactivity, and shifting dullness was negative (-). No edema was noted in the lower extremities. Auxiliary Examinations: Abdominal CT (External Hospital, 2012.10.19): 1. Abnormal density and disorganized structure in the pancreatic tail, splenic hilum, and hepatic hilum regions; 2. Low-density lesion in the posterior segment of the right hepatic lobe; 3. Mild splenomegaly; 4. Dilation and hydronephrosis in the right renal pelvis and upper ureter; 5. Gallbladder not clearly visualized; 6. Abdominal MRI (External Hospital, 2012.10.25): 1. Substantial mass in the pancreatic tail, likely pancreatic tail cancer, with lymph node metastasis in the hepatic hilum and retroperitoneal region; 2. Mild dilation of the intrahepatic and common bile ducts; 3. Dilation of the right renal pelvis and ureter; 4. Multiple small cysts in the right hepatic lobe and left kidney; 5. Small amount of ascites. Tumor Markers (External Hospital, 2012.10.20): AFP: 4.91 ng/ml, CA125: 131.2 U/ml, CA199: 147.80 U/ml, CA211: 21.26 ng/ml, CA724: 203.80 U/ml. Pathology (External Hospital, 2012.10.26): Chronic inflammation of the rectal mucosa with focal glandular atrophy. The patient’s tongue was red with a yellow greasy coating, and the pulse was wiry. | 男，58岁，因“下腹隐痛伴消瘦3月余，发现胰尾占位半月余”入院。今为进一步诊治，入住我科病房，入院时下腹部隐痛，无恶心呕吐，纳差，大便偏干2月余，使用开塞露能够解出，既往无便秘，小便黄，近3个月来体重无明显减轻。体格检查：KPS 90,神清，锁骨上淋巴结未扪及明显增大。腹平软，无压痛及反跳痛。肝肋下未及，剑突下未及，脾肋下未及。肠鸣音无亢，移动性浊音（-），双下肢无水肿。辅助检查：腹部CT（外院，2012.10.19）：1.胰尾、脾门区及肝门部密度异常、结构紊乱；2.肝右后叶下段低密度灶；3.脾脏稍大；4.右肾盂及右输尿管上段扩张积液；5.胆囊显示不清；6.腹部MRI（外院，2012.10.25）：1.胰腺尾部实质性占位，考虑：胰尾癌可能大，伴肝门部、腹膜后区淋巴结转移；2.肝内胆管、胆总管轻度扩张；3.右侧肾盂、输尿管扩张；4.肝右叶、左肾多发小囊肿；5.少量腹腔积液。肿瘤标志物(外院，2012.10.20)：AFP 4.91,CA125 131.2U/ml,CA199 147.80U/ml,CA211 21.26ng/ml,CA724 203.80U/ml。病理（外院，2012.10.26）：（直肠）粘膜慢性炎，局灶腺体萎缩。患者舌红，苔黄腻，脉弦。 |
| **1.4.3 Labeled TCM Clinical Records (Example 3)** | 1. Case Features: One month prior, the 26-year-old female patient experienced unexplained epigastric distension and pain. A mass in the pancreatic body and tail was identified through examination at an external hospital. The patient reported no significant symptoms, including fever, abdominal pain, nausea, vomiting, jaundice, abdominal distension, or diarrhea. She was admitted to our hospital for further evaluation and treatment and was diagnosed with "pancreatic tumor" at outpatient registration. Her general condition remained stable during the course of the illness. She was alert, in good spirits, and maintained normal bowel and urinary function. 2. Provisional Diagnosis and Discussion: (1) Diagnosis: Pancreatic Tumor. (2) Diagnostic Basis: 1. Female, 26 years old. 2. Admitted for "epigastric distension and pain lasting more than one month." 3. Physical Examination: ECOG performance status of 1. The patient was alert, cooperative during the examination, and exhibited no superficial lymphadenopathy. There was no jaundice of the skin or sclera. The abdomen was flat and soft, with no tenderness or rebound tenderness in the upper right quadrant. The liver, subxiphoid, and spleen were not palpable below the costal margin. No significant masses were detected. Murphy's sign was negative, as was shifting dullness. Bowel sounds were 3-5 per minute. 4. Auxiliary Examination: CT Scan (Zhongshan Hospital, 2015-10-21): Enhanced abdominal CT revealed a cystic lesion in the pancreatic tail, suggestive of a possible cystadenoma. No significant retroperitoneal lymphadenopathy was observed. MRI was recommended for further evaluation if necessary. | 一、病例特点: 患者1月前无明显诱因下自觉上腹胀痛，外院查体提示胰体尾占位。患者无明显自觉症状，无发热、腹痛、恶心呕吐，无皮肤巩膜黄染，无腹胀腹泻。为进一步治疗来我院。门诊以“胰腺肿瘤”收治入院。患者病来一般情况良好，神志清、精神可，大小便基本正常。二、拟诊讨论: (一)诊断:胰腺肿瘤 (二)诊断依据:1.患者，女，26岁2.因“上腹胀痛一月余”入院3.体检：ECOG：1，神志清，查体合作，全身浅表淋巴结未及肿大，皮肤巩膜无黄染，腹平软。右上腹深压痛阴性，无明显肌紧张、反跳痛。余腹部无明显压痛及反跳痛。肝肋下未及，剑突下未及，脾肋下未及。亦未扪及明显肿块。Murphy征阴性，移动性浊音阴性，肠鸣音3-5次/分4.辅助检查：CT:(中山医院，2015-10-21)诊断: 腹部CT增强扫描：胰尾囊性占位，考虑囊腺瘤可能，建议必要时结合MRI扫描。腹膜后未见明显肿大淋巴结。 |
| **1.4.4 Labeled TCM Clinical Records (Example 4)** | Admission History: 1. A 50-year-old female was admitted due to “pancreatic cancer with liver metastasis undergoing comprehensive treatment for over 3 years.” The patient had not undergone imaging follow-up in the past three months but reported a generally good condition. She sought further treatment and was admitted. The patient was alert, in good spirits, with poor appetite, normal sleep, and normal bowel and urinary function. However, she experienced a weight loss of 5 kg since her last discharge. 2. Physical Examination: T: 37°C, P: 80 bpm, R: 18 breaths/min, BP: 98/75 mmHg. The patient was alert, calm, and cooperative during the examination. She exhibited normal development and maintained a self-supporting position. No jaundice, petechiae, liver palms, spider angiomas, ecchymosis, redness, swelling, or subcutaneous nodules were observed. No significantly enlarged lymph nodes were palpable. No deformities of the skull. No jaundice in the sclera, pupils were equal in size and reactive to light. The tongue was midline, and the throat showed no congestion or abnormal tonsillar size. The neck was soft, with no jugular vein distention, thyroid gland enlargement, or tracheal deviation. Symmetrical chest with clear and even breathing sounds. Regular rhythm at 80 bpm, with no pathological murmurs. Flat and soft, with no tenderness or rebound tenderness. The liver, subxiphoid, and spleen were not palpable below the costal margin. No significant masses were detected. No palpable masses. No deformities; joints had normal mobility. Physiological reflexes were present, with no pathological reflexes elicited. The tongue was pale red with a white coating, and the pulse was thin. | 入院病史: 1.女，50岁；因“胰腺癌肝转移综合治疗3年余”入院；近3月多来患者未行影像学复查，一般情况好，为求进一步治疗收治入院。神清，精神好，食欲不振，睡眠可，二便正常，出院至今消瘦5kg。2.体检：T37°C，P80次/分，R18次/分，BP98/75mmHg，神志清醒，精神安静，发育正常，自动体位，检查合作；皮肤粘膜未见黄染及出血点,无肝掌、蜘蛛痣，无瘀斑、红肿及皮下结节；全身浅表淋巴结未扪及明显增大；头颅外观无畸形，巩膜无黄染、瞳孔等大，对光反应存在，伸舌居中，咽喉无充血，扁桃体大小正常；颈软，颈静脉无怒张，气管居中，甲状腺无肿大；胸廓对称，呼吸均匀、清晰，心律齐，心率80次/分，未闻及病理杂音；腹平软，无压痛及反跳痛，肝肋下未及，剑突下未及，脾肋下未及，亦未扪及明显肿块；直肠指检未及肿块，外生殖器未查；脊柱四肢无畸形，关节运动自如；生理反射存在，病理反射未引出。舌淡红，苔白，脉细。 |
| **1.4.5 Labeled TCM Clinical Records (Example 5)** | Admission History: 1. A 46-year-old female was admitted for further treatment of “pancreatic cancer with liver metastasis diagnosed 6.5 years ago, with four liver interventional therapies performed over three years ago.” At the time of admission, the patient reported fatigue, poor appetite, mild epigastric and upper abdominal pain, diarrhea with watery stools (3-4 times daily), but no fever or jaundice. She had experienced a weight loss of 5 kg in the past six months. 2. Physical Examination: T: 36.5°C, P: 78 bpm, R: 18 breaths/min, BP: 120/85 mmHg, Height: 152 cm, Weight: 52 kg. The patient was alert, calm, well-nourished, and cooperative during the examination. She exhibited normal development and maintained a self-supporting position. No jaundice, petechiae, ecchymosis, redness, swelling, or subcutaneous nodules were observed. No significantly enlarged lymph nodes were palpable. Head and Neck: No deformities of the skull. No jaundice in the sclera. Pupils were equal in size and reactive to light. The tongue was midline, and the throat showed no congestion or abnormal tonsillar size. The neck was soft, with no jugular vein distention, thyroid enlargement, or tracheal deviation. Symmetrical chest with clear and even breathing sounds. Regular rhythm at 78 bpm, with no pathological murmurs. Flat and soft, with no tenderness or rebound tenderness. The liver, subxiphoid, and spleen were not palpable below the costal margin. No significant masses were detected. No palpable masses were found during rectal examination. External genitalia were not examined. No deformities; joints had normal mobility. Physiological reflexes were present, and no pathological reflexes were elicited. The patient’s tongue was red with a yellow greasy coating, and the pulse was wiry and rapid. | 入院病史: 1.患者，女，46岁，因“确诊胰腺癌肝转移6年半，4次肝介入后3年余”入院。现为进一步治疗入院。入院时：乏力，纳差，中上腹隐痛，腹泻，水样便，一日3-4次，无发热，无黄疸。近半年消瘦10斤。2.体检：T: 36.5°C,P:78次/分,R:18次/分,BP:120/85mmHg,身高:152cm,体重: 52kg。神志清醒，精神安静，营养良好，发育正常，自动体位，检查合作。皮肤、粘膜:未见黄染及出血点。无瘀斑、红肿及皮下结节。全身浅表淋巴结未扪及明显增大。头部及其器官:外观无畸形，巩膜无黄染、瞳孔等大，对光反应存在，伸舌居中，咽喉无充血，扁桃体大小正常。颈软，颈静脉无怒张，气管居中，甲状腺无肿大。胸廓对称，呼吸均匀、清晰。心律齐，心率78次/分，未闻及病理杂音。腹平软，无压痛及反跳痛。肝肋下未及，剑突下未及，脾肋下未及。亦未扪及明显肿块。肛门及外生殖器:直肠指检未及肿块，外生殖器未查。脊柱四肢:无畸形，关节运动自如。神经系统: 生理反射存在，病理反射未引出。患者舌红，苔黄腻，脉弦数。 |
| **1.4.6 Labeled TCM Clinical Records (Example 6)** | Admission History: 1. The patient, a 46-year-old female, was admitted for further treatment of "pancreatic cancer with liver metastasis diagnosed 6.5 years ago, two months after hepatic interventional therapy." At admission, the patient reported fatigue and mild epigastric and upper abdominal pain, with no fever but mild jaundice of the skin and sclera. She experienced a weight loss of 5 kg over the past six months. 2. Physical Examination: The patient was alert, calm, well-nourished, and cooperative during the examination. She exhibited normal development and maintained a self-supporting position. No ecchymosis, redness, swelling, or subcutaneous nodules. No significantly enlarged lymph nodes were palpable. The neck was soft, with no jugular vein distention, thyroid gland enlargement, or tracheal deviation. Symmetrical chest with clear and even breathing sounds. Regular rhythm at 78 bpm, with no pathological murmurs. Flat and soft, with no tenderness or rebound tenderness. The liver, subxiphoid, and spleen were not palpable below the costal margin. No significant masses were detected. No palpable masses were found during rectal examination. External genitalia were not examined. No deformities; joints had normal mobility. Physiological reflexes were present, and no pathological reflexes were elicited. 3. Auxiliary Examinations: 2005.02.10 CT Scan: Pancreatic cancer with multiple liver metastases. 2005.04.04 Hepatic Biopsy (at our hospital): Metastatic adenocarcinoma. 2011.09.19 Follow-up CT: Multiple low-density lesions in the liver, considered metastatic tumors. A low-density lesion in the pancreatic body with possible involvement of the splenic vein. Enlarged retroperitoneal lymph nodes. High-density lesion in the left iliac bone, suspected metastasis. 2011.09.20 Gastroscopy: Atrophic gastritis with erosion and duodenal bulb ulceration. The patient’s tongue was red with a yellow greasy coating, and the pulse was wiry. | 入院病史: 1.患者，女，46岁，因“确诊胰腺癌肝转移6年半,肝介入术后2月余”入院。现为进一步治疗入院。入院时：乏力，中上腹隐痛，无发热，皮肤巩膜轻度黄染。近半年消瘦10斤。2.体检：神志清醒，精神安静，营养良好，发育正常，自动体位，检查合作。无瘀斑、红肿及皮下结节。全身浅表淋巴结未扪及明显增大。颈软，颈静脉无怒张，气管居中，甲状腺无肿大。胸廓对称，呼吸均匀、清晰。心律齐，心率78次/分，未闻及病理杂音。腹平软，无压痛及反跳痛。肝肋下未及，剑突下未及，脾肋下未及。亦未扪及明显肿块。肛门及外生殖器:直肠指检未及肿块，外生殖器未查。脊柱四肢:无畸形，关节运动自如。神经系统: 生理反射存在，病理反射未引出。3.辅检：2005.02.10CT示：胰腺癌，肝多发转移。2005.04.04我院肝穿：转移性腺癌。2011.09.19复查CT：肝内多发低密度影，考虑转移瘤，胰腺体部低密度影，占位并侵犯脾静脉可能，腹膜后肿大淋巴结，左侧髂骨高密度影，转移待排。2011.09.20胃镜：萎缩性胃炎伴糜烂，十二指肠球部溃疡。患者舌红，苔黄腻，脉弦。 |
| **1.4.7 Labeled TCM Clinical Records (Example 7)** | 1. Case Characteristics: (1) A 46-year-old female was admitted for further treatment of “pancreatic cancer with liver metastasis diagnosed 6.5 years ago, 13 months after six interventional therapies.” At admission, the patient reported fatigue, mild epigastric and upper abdominal pain, occasional nausea and acid reflux, poor appetite, and disturbed sleep. She denied vomiting, fever, or jaundice. Urine was yellow, and bowel movements were normal. She experienced an 8 kg weight loss over the past six months. (3) Physical Examination: KPS: 80. The patient was alert, in good spirits, with no ecchymosis, redness, or subcutaneous nodules. No significantly enlarged superficial lymph nodes were palpable. Abdomen: Soft and flat, with tenderness in the upper abdomen but no rebound tenderness. The liver, subxiphoid, and spleen were not palpable below the costal margin. No significant masses were detected. Shifting dullness was positive (+). Bowel Sounds: 4 per minute, no hyperactivity. No edema was observed in the lower extremities. (4) Auxiliary Examinations: CT Scan (External Hospital, 2005.02.10): Pancreatic cancer with multiple liver metastases. Pathology (Our Hospital, 2005.04.04): Hepatic biopsy confirmed metastatic adenocarcinoma. CT Scan (External Hospital, 2011.09.19): Multiple low-density lesions in the liver suggestive of metastatic tumors; low-density lesion in the pancreatic body with possible involvement of the splenic vein; enlarged retroperitoneal lymph nodes; high-density lesion in the left iliac bone, suspected metastasis. Gastroscopy (External Hospital, 2011.09.20): Atrophic gastritis with erosion and duodenal bulb ulceration. CT Scan (Jiangsu Cancer Hospital, 2013.08.14): Post-treatment findings of pancreatic cancer with liver metastasis: 1. Lesion in the pancreatic body still visible. 2. Multiple liver metastases. 3. Enlarged retroperitoneal lymph nodes. The patient’s tongue was red with a yellow greasy coating, and the pulse was wiry. | 一、病例特点: （1）患者，女，46岁，因“确诊胰腺癌肝转移6年半,6次介入术后13月”入院。现为进一步治疗入院。入院时：乏力，中上腹隐痛，时有恶心，无呕吐，时有反酸，无发热，无黄疸，纳少眠差，小便黄，大便正常。近半年体重减轻8kg。（3）体格检查：KPS80，神志清，精神可。无瘀斑、红肿及皮下结节，全身浅表淋巴结未扪及明显增大，腹平软，上腹压痛，无反跳痛，肝肋下未及，剑突下未及，脾肋下未及，亦未扪及明显肿块，移动性浊音（+），肠鸣音4次/分，无亢进。双下肢无水肿。（4）辅助检查： CT示：外院（2005.02.10）胰腺癌，肝多发转移。病理：本院（2005.04.04）肝穿示转移性腺癌。CT：外院（2011.09.19）肝内多发低密度影，考虑转移瘤，胰腺体部低密度影，占位并侵犯脾静脉可能，腹膜后肿大淋巴结，左侧髂骨高密度影，转移待排。胃镜：外院（2011.09.20）萎缩性胃炎伴糜烂，十二指肠球部溃疡。CT： 江苏省肿瘤医院（2013.08.14）胰腺癌肝转移治疗后：1.胰体部仍见病灶。2.肝多发转移3.腹腔后淋巴结肿大。（二）拟诊讨论: (一)诊断:胰腺癌肝转移。患者舌红，苔黄腻，脉弦。 |

^a^TCM: traditional Chinese medicine.
