# Supplementary material for: Identification of Syndrome Types in Patients With Pancreatic Cancer From Free Text in Electronic Medical Records: Model Development and Validation
Source: JMIR Form Res. 2025 Oct 3;9:e70602. doi: 10.2196/70602 (PMC12534766; doi:10.2196/70602)
Supplement: Multimedia Appendix 4 [file formative_v9i1e70602_app4.docx]

## Evaluation Metrics for Multi-Class Syndrome Differentiation

As this study involves a four-class classification task, we selected Macro-Precision, Macro-Recall, Macro-F1, and Accuracy as the primary evaluation metrics to comprehensively assess the model's performance in TCM syndrome differentiation.

### Macro-Precision

Precision is used to evaluate the positive predictive value of the model, indicating the proportion of correctly predicted samples among all samples predicted as a particular class. It is defined as:

$${Precision}_{i}=\frac{{TP}_{i}}{{TP}_{i}+{FP}_{i}}$$

Where ${TP}_{i}$ represents the number of samples whose true label is class $i$ and are correctly predicted as class $i$ and ${FP}_{i}$ denotes the number of samples that do not belong to class $i$ but are incorrectly predicted as class $i$.

To account for all syndrome classes equally, Macro-Precision is calculated as the unweighted average of precision values across all classes:

$$Macro-Precision=\frac{1}{C}\sum_{i=1}^{C} {Precision}_{i}$$

Where $C=4$, corresponding to the four TCM syndrome categories in this study.

### Macro-Recall

Recall is used to measure the sensitivity (or true positive rate) of the model, reflecting the proportion of correctly identified samples among all instances that truly belong to a given class. It is defined as:

$${Recall}_{i}=\frac{{TP}_{i}}{{TP}_{i}+{FN}_{i}}$$

Where ${TP}_{i}$ denotes the number of samples that are correctly predicted as class $i$, and ${FN}_{i}$ denotes the number of samples that actually belong to class $i$ but are incorrectly classified as other classes.

To provide an equal-weighted evaluation across all syndrome types, Macro-Recall is calculated as the arithmetic mean of recall values for each class:

$$Macro-Recall=\frac{1}{C}\sum_{i=1}^{C} {Recall}_{i}$$

Where $C=4$, corresponding to the four TCM syndrome categories in this study.

### Macro-F1

F1-score represents the harmonic mean of Precision and Recall, and serves as a balanced measure that simultaneously considers both the sensitivity and the precision of the model. It is calculated as:

$${F1}_{i}=\frac{{2\cdot Precision}_{i}\times{Recall}_{i}}{{Precision}_{i}+{Recall}_{i}}$$

To provide a class-balanced assessment, Macro-F1 is defined as the unweighted average of the F1-scores across all classes:

$$Macro-F1=\frac{1}{C}\sum_{i=1}^{C} {F1-Score}_{1}$$

Where $C=4$, corresponding to the four TCM syndrome categories in this study.

### Accuracy

Accuracy refers to the overall correctness of the model's predictions and is defined as the proportion of correctly classified samples among all test samples:

$$Accuracy=\frac{Number of Correct Predictions}{Total Number of Samples}$$

## McNemar’s Test for Paired Classifier Comparison

McNemar’s test is a non-parametric statistical method commonly used to evaluate whether there is a statistically significant difference in the performance of two paired classifiers on the same set of instances. It is based on a 2×2 contingency table that focuses on the discordant pairs—cases where the two models yield different predictions. This method is particularly appropriate for classification tasks involving discrete binary outcomes[1].

As this study involves a multi-class classification task, McNemar’s test was adapted using a “one-vs-rest” strategy to evaluate each syndrome category individually. For each syndrome type (e.g., damp-heat syndrome), we reformulated the task as a binary classification problem (“correctly recognized” vs. “not correctly recognized”). The test was then conducted separately for each syndrome label to assess pairwise differences between models.

For example, when comparing the performance of TCMPCSD-BERT and LSTM in identifying damp-heat syndrome, the following hypotheses were tested:

- Null hypothesis ($H_{0}$): There is no significant difference in classification accuracy between TCMPCSD-BERT and LSTM for damp-heat syndrome.
- Alternative hypothesis ($H_{1}$): There is a significant difference in classification accuracy between TCMPCSD-BERT and LSTM for damp-heat syndrome.

A 2×2 contingency table was constructed as follows:

|  | TCMPCSD-BERT Correct | TCMPCSD-BERT Incorrect | Row Total |
| --- | --- | --- | --- |
| LSTM Correct | a | b | a+b |
| LSTM Incorrect | c | d | c+d |
| Column Total | a+c | b+d | n |

In this table:

- a represents the number of cases correctly predicted by both models;
- b represents the number of cases correctly predicted by LSTM but misclassified by TCMPCSD-BERT;
- c represents the number of cases correctly predicted by TCMPCSD-BERT but misclassified by LSTM;
- d represents the number of cases misclassified by both models;
- n denotes the total number of test cases (683 in this study).

### McNemar’s Chi-Square Approximation (when b + c ≥ 25)

When the number of discordant predictions (b+c) is greater than or equal to 25, the McNemar chi-square approximation without continuity correction is considered reliable[2]. Under the null hypothesis $H_{0}$, it is assumed that b=c, indicating no significant difference in the models’ performance on discordant cases. The test statistic is calculated as:

$$x^{2}=\frac{{(b-c)}^{2}}{b+c}\sim x^{2}$$

Let $x^{2}$=$k$，be the value calculated from the observed data. The corresponding *P* value is:

$$P=Pr(x^{2}\geq k)$$

Here, $k$ refers to the chi-square statistic calculated from the observed data. The corresponding *P* value represents the probability of observing a difference as extreme as, or more extreme than, the current one, assuming the null hypothesis holds. A small *P* value (e.g., < 0.05) suggests that such a level of disagreement between the models’ predictions is unlikely to occur if their performance were truly equivalent. Consequently, this provides statistical evidence that the two models may differ significantly in their classification performance for the given syndrome type. In general, a larger chi-square value corresponds to a smaller *P* value, indicating a greater likelihood that the observed performance difference—such as between TCMPCSD-BERT and LSTM in identifying damp-heat syndrome—is statistically significant.

### Exact McNemar’s Test (for small sample sizes: b+c < 25)

When the number of discordant cases (b+c) is less than 25, the chi-square approximation becomes unreliable. In such cases, the exact McNemar’s test based on the binomial distribution is employed[2]. Under the null hypothesis $H_{0}$, each discordant pair is equally likely (with probability 0.5) to favor either of the two models—TCMPCSD-BERT or LSTM—if their performance is truly equivalent. The exact *P* value is calculated using the following formula:

$$P=2\times\sum_{i=0}^{min(b,c)} \binom{b+c}{i}\left( \frac{1}{2} \right)^{b+c}$$

Where $\binom{b+c}{i}$ denotes the number of combinations in which $i$ out of the b+c discordant samples are correctly classified by one model and misclassified by the other. The *P* value thus represents the probability of observing the current level of disagreement—or a more extreme imbalance—between the two models, assuming that their performance is identical. A small *P* value (e.g., < 0.05) indicates that such a discrepancy is unlikely under the null hypothesis and may suggest a statistically significant difference in classification performance for the specific syndrome type.

## Stratified Bootstrap Resampling and Statistical Testing for Robust Evaluation of Multi-Class Classification Performance

Bootstrap is a non-parametric resampling method that operates by repeatedly sampling with replacement from the original dataset to generate multiple resampled datasets of the same size. For each bootstrap sample, relevant statistics (e.g., Macro-Precision, Macro-Recall, Macro-F1, and Accuracy) are recalculated, and this process is repeated thousands of times to construct empirical distributions of the evaluation metrics[3]. Unlike traditional parametric inference methods that rely on assumptions such as normality or other specific distributional forms, the bootstrap approach does not require prior assumptions about the population distribution. This makes it particularly robust and flexible when dealing with complex or non-linear statistics. In recent years, bootstrap techniques have been widely adopted in both medical and machine learning research, especially for constructing confidence intervals for classification metrics and quantifying uncertainty in model performance.

In this study, to more comprehensively assess the stability and statistical uncertainty of each model’s classification performance, we adopted a bootstrap resampling strategy following prior research. Specifically, we applied the percentile bootstrap method to estimate the 95% confidence intervals (CIs) and the bootstrap means for each evaluation metric, including Macro-Precision, Macro-Recall, Macro-F1, and Accuracy. Furthermore, based on the 5,000 bootstrap estimates obtained for each metric per model, we conducted independent samples t-tests to examine whether the observed differences between models were statistically significant [4-6].

Given that this study involves a four-class classification task, applying a conventional (non-stratified) bootstrap procedure to the entire test set may result in substantial class imbalance within resampled subsets. In some cases, certain syndrome categories may even be entirely absent in individual bootstrap samples. Such imbalance could introduce evaluation bias and substantially compromise the stability of harmonized metrics such as Macro-Precision, Macro-Recall, and Macro-F1. To mitigate this issue, a stratified bootstrap strategy was adopted to ensure that the class proportions remained consistent across all resampling iterations. Specifically, the original test set was first partitioned into four subsets based on the ground-truth labels:

- Damp-heat syndrome: 169 samples;
- Spleen-deficiency syndrome: 119 samples;
- Damp-heat with spleen-deficiency syndrome: 118 samples
- Others: 277 samples.

Then, stratified resampling with replacement was conducted independently within each class as follows:

- Subset 1: 169 samples were drawn with replacement from the damp-heat syndrome subset;
- Subset 2: 119 samples were drawn with replacement from the spleen-deficiency syndrome subset;
- Subset 3: 118 samples were drawn with replacement from the damp-heat with spleen-deficiency syndrome subset;
- Subset 4: 277 samples were drawn with replacement from the Others subset.

These four subsets were subsequently combined to form a complete bootstrap sample of 683 records, thereby completing one iteration of stratified bootstrap sampling.

Following the procedure described above, we performed 5,000 iterations of stratified bootstrap resampling on the prediction results of each model. For each bootstrap sample, four evaluation metrics—Macro-Precision, Macro-Recall, Macro-F1, and Accuracy—were computed. This process yielded 5,000 bootstrap estimates for each metric per model. Based on these estimates, we constructed 95% bootstrap confidence intervals (Bootstrap 95% CIs) using the percentile method, and calculated the bootstrap mean (i.e., the mean of the bootstrap estimates) for each metric. The bootstrap mean is defined as:

$$\hat{\theta}_{boot.mean}=\frac{1}{B}\sum_{b=1}^{B} \hat{\theta}^{(b)}$$

Where $\hat{\theta}^{(b)}$ denotes the value of the statistic (e.g., precision) computed on the $b$-th bootstrap sample, and $B$ is the total number of bootstrap iterations (in this study, $B$=5000).

Given that the bootstrap resampling in this study was performed independently for each model based on their respective predictions on the same test set, and that the bootstrap samples for each model are mutually independent, we employed statistical tests to evaluate performance differences between models. Specifically, after obtaining the distribution of each metric (Macro-Precision, Macro-Recall, Macro-F1, and Accuracy) across 5,000 stratified bootstrap samples per model, we applied independent samples t-tests to examine whether the observed differences between TCMPCSD-BERT and the other models were statistically significant. When the bootstrap estimates satisfied the assumptions of normality and homogeneity of variance, the classical independent samples t-test was used. In cases where unequal variances were detected, Welch’s t-test was adopted as an alternative. If the distribution of the metrics exhibited substantial deviation from normality and could not be normalized through transformation, the non-parametric Mann–Whitney U test was employed instead.

## Reference

1. Kim S, Lee W: **Does McNemar's test compare the sensitivities and specificities of two diagnostic tests?** *Stat Methods Med Res* 2017, **26**(1):142-154.

2. Hazra A, Gogtay N: **Biostatistics Series Module 4: Comparing Groups - Categorical Variables**. *Indian J Dermatol* 2016, **61**(4):385-392.

3. Hinkley D: **Bootstrap Methods: Another Look at the Jackknife**. 2008.

4. Jung H, Lee H, Nam EW: **Mediating Effect of Social Capital on the Association Between Digital Literacy and Life Satisfaction Among Older Adults in South Korea: Cross-Sectional Study**. *JMIR Form Res* 2025, **9**:e68163.

5. Sinci KA, Koska IO, Cetinoglu YK, Erdogan N, Koc AM, Eliyatkin NO, Koska C, Candan B: **Deep learning-based classification of parotid gland tumors: integrating dynamic contrast-enhanced MRI for enhanced diagnostic accuracy**. *BMC Med Imaging* 2025, **25**(1):264.

6. Chu CW, Lu CM, Yeung WK: **Systematic Evaluation of Atrous Spatial Pyramid Pooling in U-Net for Pore Segmentation in Plasma Electrolytic Oxidation Coatings**. *Langmuir* 2025, **41**(25):16368-16377.
