# Supplementary material for: Identification of Syndrome Types in Patients With Pancreatic Cancer From Free Text in Electronic Medical Records: Model Development and Validation
Source: JMIR Form Res. 2025 Oct 3;9:e70602. doi: 10.2196/70602 (PMC12534766; doi:10.2196/70602)
Supplement: Multimedia Appendix 5 [file formative_v9i1e70602_app5.docx]

To further quantify the model's reliance on specific symptoms and signs in the syndrome differentiation task, we applied the Integrated Gradients algorithm[1] to assess the contribution of each term in the clinical record text to syndrome prediction. Among the key principles of the Integrated Gradients algorithm are "sensitivity" and "implementation invariance."

### Sensitivity

Sensitivity requires that the model's output should change in response to variations in the input features. When changes in the input features affect the model’s output, the Integrated Gradients algorithm should be able to reflect this variation. For example, in the task of differentiating Damp-heat syndrome, if the presence of a symptom (such as "yellow tongue") significantly influences the model's output (e.g., causing the model to predict Damp-Heat syndrome), the Integrated Gradients algorithm should capture this change and assign a high attribution score to "yellow tongue."

### Implementation Invariance

Implementation invariance requires that the model's explanation should be independent of the specific implementation of the input representation. In other words, different input transformations or representations should not affect the model's explanation. Suppose we have two different models with distinct internal architectures and computational paths, but they produce consistent outputs for the same input (e.g., clinical symptom descriptions). If we apply the Integrated Gradients algorithm for attribution analysis on both models, and the attribution results (such as the importance of "yellow tongue" in Damp-Heat syndrome prediction) are the same regardless of which model is used, then these two models are considered functionally equivalent, and the attribution algorithm used satisfies the condition of implementation invariance. Specifically, if the attribution analysis yields consistent results across models with different implementation paths, and the attribution responsibility is entirely attributed to the input data rather than the internal implementation of the model, then the attribution method satisfies implementation invariance. This helps ensure the reliability and consistency of attribution analysis, avoiding interference from internal implementation details in the interpretation results.

Before the introduction of the "Integrated Gradients algorithm," "gradient-based attribution[2]” and "backpropagation-based attribution[1]” were the two main attribution methods.

### Gradient Attribution

Gradient attribution primarily measures the contribution of each input feature to the model's prediction by directly calculating the "gradient of the output with respect to the input." The calculation method is simple and intuitive. However, in the case of deep learning, neural networks themselves are nonlinear function representations, which can be viewed as a stack of multiple nonlinear functions. Nonlinear functions inevitably exhibit stages of gradient saturation. For instance, consider the commonly used ReLU function:

$$f\left( x \right)=1-ReLU\left( 1-x \right)=\left\{ \begin{aligned} x, x<1 \\ 1,x>1 \end{aligned} \right.$$

The $ReLU$activation function is typically defined as $ReLU\left( z \right)=max(0, z)$, meaning if $z$ is positive, the output is $z$, otherwise the output is 0. In the example function above, if $\left( 1-x \right)$ is positive, $ReLU$ outputs $1-x$; however, when $x\geq1$, the $ReLU$ output is always 0, which violates the "sensitivity" axiom, as the model's output no longer changes with variations in the input features.

Regarding "implementation invariance," if we assume that the nonlinear function is differentiable everywhere within its domain, and let $i$ be the input and $h$ be the model's internal implementation details, by applying the chain rule, we obtain:

$$\frac{\partial f}{\partial i}=\frac{\partial f}{\partial h}\cdot\frac{\partial h}{\partial i}$$

This formula demonstrates that based on the direct gradient information between input and output, we can ignore the internal implementation details of the model. Therefore, this attribution method satisfies implementation invariance[1].

### Backpropagation-based Attribution

Backpropagation-based attribution algorithms typically avoid the "gradient saturation" problem by assigning larger discrete values to the input features. Using the previously mentioned $ReLU$ activation function as an example, when the input $x\geq1$, , the output of $ReLU$ is always 0, leading to gradient vanishing. To prevent gradient vanishing, one common approach is to "alter the magnitude or value range of the input features" or to "use appropriate bias values" to ensure that the input feature's value range does not enter the negative region of the $ReLU$ function, thus avoiding gradient vanishing.

However, setting large discrete values may trigger a "step phenomenon," leading the attribution algorithm to no longer satisfy the chain rule. Specifically, changes in input values could cause the function to exhibit discontinuities in certain regions, resulting in a failure to uphold the chain rule:

$$\frac{f\left( x_{1} \right)-f(x_{0})}{i\left( x_{1} \right)-i(x_{0})}\neq\frac{f\left( x_{1} \right)-f(x_{0})}{h\left( x_{1} \right)-h(x_{0})}\cdot\frac{h\left( x_{1} \right)-h(x_{0})}{i\left( x_{1} \right)-i(x_{0})}$$

This "discontinuity" indicates that the internal implementation details of the function cannot be ignored, thereby failing to satisfy implementation invariance.

### Integrated Gradients Algorithm

The Integrated Gradients algorithm combines the ideas of "direct gradients in gradient-based attribution" and "backpropagation-based attribution" to simultaneously satisfy the two key axioms of "sensitivity" and "implementation invariance." This algorithm estimates the contribution of each input feature to the model output by calculating the change between the input and the baseline input (e.g., a zero or blank state), which can be expressed as:

$${IntegratedGradients(x}_{i})=\left( x_{i}-x_{i}^{'} \right)\times\int_{a=0}^{1} \frac{\partial F(x_{i}^{'}+\alpha\left( x_{i}-x_{i}^{'} \right))}{\partial x_{i}^{'}}d\alpha$$

$x_{i}$ represents the $i$-th input feature;

$x_{i}^{'}$ represents the baseline value corresponding to $x_{i}$;

$F$ represents the TCMPCSD-BERT model in this study;

$\alpha$ is the interpolation parameter, used to generate a "gradual transition from the baseline value $x_{i}^{'}$ to $x_{i}$” with $\alpha$ ranging from 0 to 1.

For this study, suppose we use the Integrated Gradients algorithm to evaluate the impact of "yellow tongue" on the model's prediction of "Damp-heat syndrome." In this case:

- $x_{i}$ represents the semantic representation of "yellow tongue" (after embedding into a word vector);
- $x_{i}^{'}$ is the baseline value corresponding to "yellow tongue" (i.e., the blank state);
- $x_{i}-x_{i}^{'}$ represents the semantic difference of the "yellow tongue" feature relative to the featureless state, reflecting the impact of the "yellow tongue" feature expressed in the TCMPCSD-BERT model;
- The interpolation process $\alpha\left( x_{i}-x_{i}^{'} \right)$ with $\alpha$∈[0, 1] is used for a gradual transition from the baseline value $x_{i}^{'}$ to the actual input feature $x_{i}$, forming a weighted gradient of the "yellow tongue" feature. This weighted gradient reflects the transition of the input feature from weak to strong. Through this gradual transition, the interpolation process helps ensure the smooth propagation of the gradient, effectively avoiding issues like "gradient vanishing" or "discontinuity."
- $F(x_{i}^{'}+\alpha\left( x_{i}-x_{i}^{'} \right)$ is the output of the TCMPCSD-BERT model when the input value is $(x_{i}^{'}+\alpha\left( x_{i}-x_{i}^{'} \right)$, i.e., as the interpolation $\alpha$ changes from 0 to 1, the input gradually transitions from the baseline value $x_{i}^{'}$ (i.e., the state with no "yellow tongue" feature) to the actual input $x_{i}$ (i.e., the state with the "yellow tongue" feature). Through this gradual transition, we can observe the impact of the "yellow tongue" feature on the confidence value for the prediction of "Damp-heat syndrome" by the TCMPCSD-BERT model.
- $\frac{\partial F(x_{i}^{'}+\alpha\left( x_{i}-x_{i}^{'} \right))}{\partial x_{i}^{'}}$ is the change rate of the model output as the input transitions from the baseline $x_{i}^{'}$ to the target input $x_{i}$, and ultimately, by integrating over different interpolation weights $\alpha$, we obtain the total contribution of the "yellow tongue" feature to the prediction of "Damp-heat syndrome" by the TCMPCSD-BERT model.。

In traditional direct gradient attribution algorithms, when the input feature values are at the gradient saturation stage, the attribution weight obtained for that feature tends to be negligible. However, this does not imply that the input feature is unimportant. The Integrated Gradients algorithm, by selecting an infinite number of integration points between the baseline value and the input value for integration summation, resolves the gradient saturation problem and adheres to the sensitivity axiom. Moreover, the Integrated Gradients algorithm relies solely on the relationship between input and output, independent of the model's specific implementation, thus also satisfying the "implementation invariance" axiom.

Therefore, in this study, we will use the Integrated Gradients algorithm to quantify the impact of each word or symptom on the prediction results. For example, in clinical records, symptoms such as "abdominal pain" and "yellow, greasy tongue coating" may significantly influence the prediction of Damp-heat syndrome. Through Integrated Gradients analysis, these symptoms will receive higher attribution scores, reflecting their key role in the model’s prediction, while symptoms with lower attribution scores indicate minimal or negligible impact on the prediction.

## Reference

1. Sundararajan M, Taly A, Yan Q. Axiomatic Attribution for Deep Networks. 2017:

2. Shrikumar A, Greenside P, Shcherbina A, Kundaje A. Not Just a Black Box: Learning Important Features Through Propagating Activation Differences. ArXiv. 2016;abs/1605.01713
