# Supplementary material for: Identification of Syndrome Types in Patients With Pancreatic Cancer From Free Text in Electronic Medical Records: Model Development and Validation
Source: JMIR Form Res. 2025 Oct 3;9:e70602. doi: 10.2196/70602 (PMC12534766; doi:10.2196/70602)
Supplement: Multimedia Appendix 6 [file formative_v9i1e70602_app6.docx]

## Large Language Models (LLMs) Prompt Engineering

### Prompt Design

In terms of prompt design, this study drew inspiration from parts of the MedPrompt strategy proposed by the Microsoft team in 2023[1] and recent similar studies[2,3]. By integrating the characteristics of TCM syndrome differentiation tasks, the following prompt design scheme was initially developed:

**(1) Role-Play Prompting**: Role-Play Prompting is defined as the approach where a clear identity and task background are assigned to the model in Prompt design, guiding it to be focused on specific tasks and enhancing its concentration and execution capabilities[4]. In this study, the model is designated as "a TCM expert in the field of integrated Chinese and Western medicine for cancer treatment" by this approach. By being assigned such an identity, the model's thought framework is brought closer to the professional scope of TCM theory, thereby being focused on the execution of the task of syndrome differentiation and classification for pancreatic cancer. To further improve the model's adaptability to the task, the syndrome differentiation elements and standards that need to be followed are outlined in this study, providing clear rule guidance for the task. This design is aimed at reducing deviations and hallucination generation by the model during task execution, ensuring that it is better adapted to the task requirements.

**(2) In-Context Learning (ICL)**: In-Context Learning (ICL) is an effective method that assists LLMs in understanding task procedures and output formats by providing them with a small number of high-quality examples before the formal task execution[5]. In this study, experts have selected two representative example cases for each syndrome type (due to the relatively smaller parameters of the Chinese model, only one case is selected for each label). Furthermore, to avoid potential biases arising from a fixed input order, the input sequence of the examples is dynamically adjusted by generating random numbers each time ICL is conducted in a new conversation. This allows the examples to be input into the LLMs in different orders, thereby better guiding them through the specific operational procedures of the syndrome differentiation task.

**(3)** **Chain-of-Thought (CoT)**: Chain-of-Thought (CoT) enhances the performance of LLMs in complex reasoning tasks by guiding them to generate intermediate reasoning steps, thereby breaking down complex problems into clear logical chains[6]. CoT typically relies on a small number of high-quality examples manually crafted by experts, which not only include questions and answers but also explicitly demonstrate the reasoning pathways. These examples serve as Prompt inputs to help the model better understand task logic and generate accurate results[7]. In this study, the approach of manually crafting Chain-of-Thought examples is adopted, with high-quality reasoning examples designed by experts in the field of TCM. These examples systematically showcase the core basis of TCM syndrome differentiation, including the step-by-step analysis process of primary symptoms, secondary symptoms, tongue features, and pulse features. They provide a clear logical framework for LLMs, guiding the model to complete the syndrome differentiation task.

**(4) Choice Shuffling Ensemble**: Studies have shown that LLMs may exhibit a preference for specific option positions when faced with multiple-choice questions, independent of the option content itself[8]. To eliminate this positional bias, this study dynamically adjusts the order of the four syndrome type labels using a random sequence generation method and performs syndrome differentiation five times for each case. The specific process includes: randomly regenerating the permutation order of the syndrome type labels for each syndrome differentiation task; using LLMs to perform five independent syndrome differentiations on the same case, each time with a different label order; and finally, selecting the syndrome type label that appears most frequently as the final syndrome differentiation result for the case through voting statistics based on the five results. This method effectively reduces positional bias in the syndrome differentiation process by introducing a mechanism of option shuffling and voting ensemble, thereby improving the stability and reliability of the results. In this step, this study refers to the design scheme of similar clinical studies, simplifies the experimental process, and does not fully follow the multi-temperature parameter setting and large-scale permutation combination strategy suggested in the MedPrompt method[2,3].

To mitigate the impact of context forgetting and model hallucination issues in LLMs when dealing with long text tasks, this study rebuilds the dialogue interface every 15 questions, reloads the task context and rules, and repeats the processes of ICL, CoT, and Choice Shuffling Ensemble until all cases have completed syndrome differentiation[9].

### Construction of Test Set and Evaluation of LLMs' Syndrome Differentiation Performance

**Construction of Test Set**: To evaluate the application performance of LLMs in the syndrome differentiation task, this study randomly selected 100 case records for each syndrome type label from the annotated pancreatic cancer case dataset, ultimately constructing a Prompt test set comprising 400 case records.

**Performance Evaluation**: Based on the aforementioned test set, this study conducted three independent tests for each LLM. After each test, four metrics—Macro-Precision, Macro-Recall, Macro-F1, and Macro-Accuracy—were calculated. Finally, the average values from the three tests were used to assess the overall performance of each LLM in the syndrome differentiation task. The performance was also compared with that of the TCMPCSD-BERT model to verify its relative effectiveness in the pancreatic cancer syndrome differentiation task. The Prompt prompting framework used in this study is detailed in Supplementary Table 3.

### Supplementary Table 3. Main prompt framework and questioning process for prompt engineering

| **Step** | **Action** |
| --- | --- |
| **1** | Role-playing Prompt: Establish the role of a "TCM Expert", clarifying the task background, syndrome differentiation elements, and rules. |
| **2** | In-Context Learning and Chain-of-Thought: Provide 8/4 examples in random order, demonstrating how to gradually analyze and determine the syndrome type based on the patient's case content:  (2.1) Input the complete patient case as the basis for reasoning and analysis.  (2.2) Extract the primary and secondary symptom features from the case and map them to the corresponding syndrome differentiation elements.  (2.3) Extract the tongue and pulse characteristics from the case and map them to the corresponding syndrome differentiation elements.  (2.4) Synthesize the clinical features, compare them with the syndrome differentiation standards, and output the final syndrome differentiation result. |
| **3** | Formal Questioning:  (3.1) Input the complete case content that needs syndrome differentiation.  (3.2) Pose the question.  (3.3) Guide the model to analyze and judge according to the method outlined in steps 2.1 to 2.4.  (3.4) Output the syndrome differentiation result.  (3.5) Repeat the process from step 3.1 to 3.4 for a total of 5 times, tally all results, and select the syndrome type label that appears most frequently as the final syndrome differentiation result for the case (to avoid model bias towards fixed options, randomize the result options for each questioning). |
| **4** | Input New Case and Repeat Questioning Process: Input the second case that needs syndrome differentiation and repeat the questioning process outlined in step 3. Continue this process for each subsequent case until all cases have been analyzed. |
| **5** | Rebuild Dialogue Interface: When the total number of questions (starting from step 3) reaches 15, rebuild the dialogue interface and repeat the Prompt guidance and questioning process from steps 1 to 4.^a^ |

^a^ To reduce potential context forgetting issues in long text tasks, during steps 1 and 2, the model only needs to understand the Prompt guidance content without providing answers. From step 3 onwards, the model actively participates in answering questions.

## Reference

1. Nori H, Lee YT, Zhang S, et al. Can Generalist Foundation Models Outcompete Special-Purpose Tuning? Case Study in Medicine. ArXiv. 2023;abs/2311.16452

2. Wang X, Wei J, Schuurmans D, Le Q, Chi EH, Zhou D. Self-Consistency Improves Chain of Thought Reasoning in Language Models. ArXiv. 2022;abs/2203.11171

3. Zhang J, Sun K, Jagadeesh A, et al. The potential and pitfalls of using a large language model such as ChatGPT, GPT-4, or LLaMA as a clinical assistant. J Am Med Inform Assoc. Sep 1 2024;31(9):1884-1891. doi:10.1093/jamia/ocae184

4. Kong A, Zhao S, Chen H, et al. Better Zero-Shot Reasoning with Role-Play Prompting. 2023:

5. Brown TB, Mann B, Ryder N, et al. Language Models are Few-Shot Learners. ArXiv. 2020;abs/2005.14165

6. Wei J, Wang X, Schuurmans D, et al. Chain of Thought Prompting Elicits Reasoning in Large Language Models. ArXiv. 2022;abs/2201.11903

7. Singhal K, Tu T, Gottweis J, et al. Towards Expert-Level Medical Question Answering with Large Language Models. ArXiv. 2023;abs/2305.09617

8. Ko M, Lee J, Kim H, Kim G, Kang J. Look at the First Sentence: Position Bias in Question Answering. 2020:

9. Liu NF, Lin K, Hewitt J, et al. Lost in the Middle: How Language Models Use Long Contexts. Transactions of the Association for Computational Linguistics. 2023;12:157-173.
