# Supplementary material for: Identification of Syndrome Types in Patients With Pancreatic Cancer From Free Text in Electronic Medical Records: Model Development and Validation
Source: JMIR Form Res. 2025 Oct 3;9:e70602. doi: 10.2196/70602 (PMC12534766; doi:10.2196/70602)
Supplement: Multimedia Appendix 7 [file formative_v9i1e70602_app7.docx]

## The Prompt Framework for Large Language Models in English （for ChatGPT4、ChatGPT4o）

**Next, I will provide an example of “Role-Play Prompting” design, an example of “** **In-Context Learning and Chain-of-Thought,” as well as eight subsequent task instances. Please read and review these materials without responding. After I complete all 10 stages of the presentation and pose specific questions, I will then ask you to address my inquiries accordingly.**

### Role-Play Prompting

You are a traditional Chinese medicine (TCM) expert specializing in the integrative treatment of oncology, particularly pancreatic cancer. Based on the patient's medical case information and the following “diagnostic elements and criteria,” determine which of the following four syndromes the patient's condition belongs to:

1. Damp-Heat Syndrome

2. Spleen-Deficiency Syndrome

3. Damp-Heat with Spleen-Deficiency Syndrome

4. Others

Below are the detailed diagnostic elements for each syndrome, including primary symptoms, tongue features, pulse characteristics, and secondary symptoms.

- **Syndrome: Damp-Heat Syndrome**
- **Primary Symptoms:** yellowish skin; yellow sclera; yellow urine; gray-white stool; loss of appetite; nausea; vomiting; abdominal bloating; abdominal pain.
- **Secondary Symptoms:** persistent low-grade fever; thirst without strong desire to drink; bitter taste; foul breath; foul-smelling loose stools; restlessness.
- **Primary Tongue:** yellow coating; yellow greasy coating.
- **Primary Pulse Indicators:** wiry, rapid pulse
- **Additional Tongue:** red tongue
- **Additional Pulse Indicators:** rapid pulse
- **Syndrome: Spleen-Deficiency Syndrome**
- **Primary Symptoms:** fatigue; weakness; shortness of breath; dull lower back pain; fullness in chest and abdomen, aggravated after meals; loose stools; indigestion.
- **Secondary Symptoms:** small appetite; emaciation; shortness of breath; spontaneous sweating; aversion to cold; cold limbs; discomfort or pain in the upper abdomen relieved by pressure; facial puffiness; pale complexion; aversion to wind; dry mouth with little desire to drink; nausea; vomiting; edema in lower limbs; ascites.
- **Primary Tongue:** pale and swollen tongue.
- **Primary Pulse Indicators:** deficient pulse.
- **Additional Tongue:** teeth marks on the tongue edges; white slippery coating; thin white coating; pale tongue; thin coating; thin greasy coating.
- **Additional Pulse Indicators:** deep and thin pulse; thin and weak pulse; deep and slow pulse; thin pulse; thin wiry pulse; soft and moderate pulse.

**(1) Diagnostic Criteria for Single Syndrome Types:**

- The syndrome is identified if two primary symptoms are present along with any primary tongue or pulse indicator.
- It is also identified if two primary symptoms and one secondary symptom are present, along with any tongue or pulse indicator related to this syndrome.
- Additionally, the syndrome can be identified if one primary symptom and at least two secondary symptoms are present, along with any related tongue or pulse indicator.

**(2) Damp-Heat with Spleen-Deficiency Syndrome is defined by clinical features that simultaneously meet the criteria for both damp-heat and spleen-deficiency syndromes.**

**(3) Others:**

For patients who do not meet any of the above diagnostic criteria, or for whom the case information is insufficient to make a clear classification, they will be uniformly categorized as “Other.”

### In-Context Learning and Chain-of-Thought

Next, we will demonstrate how to systematically analyze and determine a patient’s syndrome type based on their medical case information through examples. In these examples, we will analyze the patient’s “primary symptoms,” “secondary symptoms,” “primary tongue indicators,” “additional tongue indicators,” “primary pulse indicators”and “additional pulse indicators.” All relevant information will be integrated and compared against the diagnostic criteria of each syndrome type, ultimately identifying the patient’s syndrome type.

In the following examples, each patient’s medical case will be analyzed in the following steps:

**(1) Input of Medical Case Information**

The details of the patient’s case will be provided as input.

**(2) Analysis of Primary and Secondary Symptoms**

- Feature Extraction: Identify and extract diagnostically significant symptoms and signs from the case information.
- Mapping Diagnostic Elements: Map the extracted symptom features to the corresponding diagnostic elements of Damp-Heat Syndrome or Spleen-Deficiency Syndrome (primary symptoms/secondary symptoms).

**(3) Analysis of Tongue and Pulse Features**

- Identification of Tongue and Pulse Features: Identify and extract diagnostically significant tongue and pulse characteristics from the case information.
- Mapping Diagnostic Elements: Map the extracted tongue and pulse characteristics to the corresponding diagnostic elements of Damp-Heat Syndrome or Spleen-Deficiency Syndrome (primary tongue indicators/additional tongue features; primary pulse features/ additional pulse indicators).

**(4) Integrated Analysis and Determination**

- Integration of Features: Integrate information on primary symptoms, secondary symptoms, tongue features, and pulse features, comparing them with the diagnostic criteria to identify all diagnostic element characteristics of the patient.
- Output of Diagnostic Results: Provide the final diagnostic conclusion.

### Example 1: Damp-Heat Syndrome

**(1) Input of Medical Case Information:**

男，58岁，因“下腹隐痛伴消瘦3月余，发现胰尾占位半月余”入院。今为进一步诊治，入住我科病房，入院时下腹部隐痛，无恶心呕吐，纳差，大便偏干2月余，使用开塞露能够解出，既往无便秘，小便黄，近3个月来体重无明显减轻。体格检查：KPS 90,神清，锁骨上淋巴结未扪及明显增大。腹平软，无压痛及反跳痛。肝肋下未及，剑突下未及，脾肋下未及。肠鸣音无亢，移动性浊音（-），双下肢无水肿。辅助检查：腹部CT（外院，2012.10.19）：1.胰尾、脾门区及肝门部密度异常、结构紊乱；2.肝右后叶下段低密度灶；3.脾脏稍大；4.右肾盂及右输尿管上段扩张积液；5.胆囊显示不清；6.腹部MRI（外院，2012.10.25）：1.胰腺尾部实质性占位，考虑：胰尾癌可能大，伴肝门部、腹膜后区淋巴结转移；2.肝内胆管、胆总管轻度扩张；3.右侧肾盂、输尿管扩张；4.肝右叶、左肾多发小囊肿；5.少量腹腔积液。肿瘤标志物(外院，2012.10.20)：AFP 4.91,CA125 131.2U/ml,CA199 147.80U/ml,CA211 21.26ng/ml,CA724 203.80U/ml。病理（外院，2012.10.26）：（直肠）粘膜慢性炎，局灶腺体萎缩。患者舌红，苔黄腻，脉弦。

Question: Please determine which syndrome type the patient belongs to:

A. Spleen-Deficiency Syndrome

B. Damp-Heat Syndrome

C. Others

D. Damp-Heat with Spleen-Deficiency Syndrome

**(2) Analysis of Primary and Secondary Symptoms**

- **Feature Extraction:** Identify and extract diagnostically significant symptoms and signs from the case information.

Primary Symptoms: 下腹隐痛 (abdominal pain); 纳差 (loss of appetite); 小便黄 (yellow urine).

Secondary Symptoms: 纳差（small appetite）；腹腔积液（ascites）

- **Mapping Diagnostic Elements**: Map the extracted symptom features to the corresponding diagnostic elements of Damp-Heat Syndrome or Spleen-Deficiency Syndrome (primary symptoms/secondary symptoms).

**Damp-Heat Syndrome:**

Abdominal pain (matches the primary symptoms of Damp-Heat Syndrome)

Yellow urine (matches the primary symptoms of Damp-Heat Syndrome)

Loss of appetite (matches the primary symptoms of Damp-Heat Syndrome)

**Spleen-Deficiency Syndrome:**

Small appetite（matches the secondary symptoms of Spleen-Deficiency Syndrome）

Ascites（matches the secondary symptoms of Spleen-Deficiency Syndrome）

**(3) Analysis of Tongue and Pulse Features**

- **Identification of Tongue and Pulse Features:** Identify and extract diagnostically significant tongue and pulse characteristics from the case information.

Tongue Features：舌红 (red tongue): 苔黄腻 (yellow greasy coating)

Pulse Features: 脉弦 (wiry, rapid pulse); 脉弦 (thin wiry pulse)

- **Mapping Diagnostic Elements:** Map the extracted tongue and pulse features to the corresponding diagnostic elements of Damp-Heat Syndrome or Spleen-Deficiency Syndrome (primary tongue indicators/additional tongue indicators; primary pulse indicators/additional pulse indicators).
- **Damp-Heat Syndrome:**

Yellow greasy coating（matches the primary tongue indicators of Damp-Heat Syndrome）。

Red tongue（matches the additional tongue indicators of Damp-Heat Syndrome）

Wiry, rapid pulse（matches the primary pulse indicators of Damp-Heat Syndrome）

- **Spleen-Deficiency Syndrome:**

Thin wiry pulse（matches the additional pulse indicators of Spleen-Deficiency Syndrome）

**Integrated Analysis and Determination:**

The patient exhibits three primary symptoms of Damp-Heat Syndrome (abdominal pain, loss of appetite, yellow urine), one primary tongue feature (yellow, greasy coating), one additional tongue feature (red tongue), and one primary pulse feature (wiry, rapid pulse), meeting the diagnostic criteria for Damp-Heat Syndrome.

The patient also exhibits two secondary symptoms of Spleen-Deficiency Syndrome (small appetite, ascites) and one additional pulse feature (thin wiry pulse), which do not meet the diagnostic criteria for Spleen-Deficiency Syndrome.

**Conclusion:** The patient is classified as: B. Damp-Heat Syndrome.

### Example 2: Damp-Heat with Spleen-Deficiency Syndrome

**(1) Input of Medical Case Information:**

（1）患者男，58岁。（2）因“胰腺癌盆腔转移2月，介入化疗后1月余”入院。为进一步治疗再次入院。入院时：下腹部痛，纳差，小便色黄，量正常，大便不畅，无发热，无黄疸，出院至今消瘦3公斤。（4）体格检查：KPS 90,神清，锁骨上淋巴结未扪及明显增大。腹平软，下腹部压痛（+），反跳痛（-），肝肋下未及，剑突下未及，脾肋下未及。肠鸣音无亢，移动性浊音（+）。右下肢大腿正面可见一陈旧性手术疤痕，长约50cm,愈合良好，双下肢无凹陷性水肿。患者舌红，苔黄腻，脉弦。

Question: Please determine which syndrome type the patient belongs to:

A. Damp-Heat Syndrome

B. Spleen-Deficiency Syndrome

C. Damp-Heat with Spleen-Deficiency Syndrome

D. Others

**(2) Analysis of Primary and Secondary Symptoms**

- **Feature Extraction:** Identify and extract diagnostically significant symptoms and signs from the case information.

Primary Symptoms: 下腹部痛，下腹部压痛（+）（abdominal pain）；纳差（loss of appetite）；小便色黄（yellow urine）。

Secondary Symptoms: 纳差（small appetite）；腹腔积液，移动性浊音（+）（ascites）；出院至今消瘦3公斤（emaciation）；双下肢无凹陷性水肿（edema in lower limbs）

- **Mapping Diagnostic Elements**: Map the extracted symptom features to the corresponding diagnostic elements of Damp-Heat Syndrome or Spleen-Deficiency Syndrome (primary symptoms/secondary symptoms).
- **Damp-Heat Syndrome:**

Abdominal pain (matches the primary symptoms of Damp-Heat Syndrome)

Loss of appetite (matches the primary symptoms of Damp-Heat Syndrome)

Yellow urine (matches the primary symptoms of Damp-Heat Syndrome)

- **Spleen-Deficiency Syndrome:**

Small appetite（matches the secondary symptoms of Spleen-Deficiency Syndrome）

Ascites（matches the secondary symptoms of Spleen-Deficiency Syndrome）

Emaciation（matches the secondary symptoms of Spleen-Deficiency Syndrome）

Edema in lower limbs（matches the secondary symptoms of Spleen-Deficiency Syndrome）

**(3) Analysis of Tongue and Pulse Features**

- **Identification of Tongue and Pulse Features:** Identify and extract diagnostically significant tongue and pulse characteristics from the case information.

Tongue Features：舌红 (red tongue): 苔黄腻 (yellow greasy coating)

Pulse Features: 脉弦 (wiry, rapid pulse); 脉弦 (thin wiry pulse)

- **Mapping Diagnostic Elements:** Map the extracted tongue and pulse features to the corresponding diagnostic elements of Damp-Heat Syndrome or Spleen-Deficiency Syndrome (primary tongue indicators/additional tongue indicators; primary pulse indicators/additional pulse indicators).
- **Damp-Heat Syndrome:**

Yellow greasy coating（matches the primary tongue indicators of Damp-Heat Syndrome）。

Red tongue（matches the additional tongue indicators of Damp-Heat Syndrome）

Wiry, rapid pulse（matches the primary pulse indicators of Damp-Heat Syndrome）

- **Spleen-Deficiency Syndrome:**

Thin wiry pulse（matches the additional pulse indicators of Spleen-Deficiency Syndrome）

**Integrated Analysis and Determination:**

The patient exhibits three primary symptoms of Damp-Heat Syndrome (abdominal pain, loss of appetite, yellow urine), one primary tongue feature (yellow, greasy coating), one additional tongue feature (red tongue), and one primary pulse feature (wiry, rapid pulse), meeting the diagnostic criteria for Damp-Heat Syndrome.

The patient also exhibits four secondary symptoms of Spleen-Deficiency Syndrome (small appetite, ascites, emaciation, edema in lower limbs) and one additional pulse feature (thin wiry pulse), meeting the diagnostic criteria for Spleen-Deficiency Syndrome.

**Conclusion:** The patient is classified as: C. Damp-Heat with Spleen-Deficiency Syndrome.

### Example 3: Others

**(1) Input of Medical Case Information:**

一、病例特点: 患者1月前无明显诱因下自觉上腹胀痛，外院查体提示胰体尾占位。患者无明显自觉症状，无发热、腹痛、恶心呕吐，无皮肤巩膜黄染，无腹胀腹泻。为进一步治疗来我院。门诊以“胰腺肿瘤”收治入院。患者病来一般情况良好，神志清、精神可，大小便基本正常。二、拟诊讨论: (一)诊断:胰腺肿瘤 (二)诊断依据:1.患者，女，26岁2.因“上腹胀痛一月余”入院3.体检：ECOG：1，神志清，查体合作，全身浅表淋巴结未及肿大，皮肤巩膜无黄染，腹平软。右上腹深压痛阴性，无明显肌紧张、反跳痛。余腹部无明显压痛及反跳痛。肝肋下未及，剑突下未及，脾肋下未及。亦未扪及明显肿块。Murphy征阴性，移动性浊音阴性，肠鸣音3-5次/分4.辅助检查：CT:(中山医院，2015-10-21)诊断: 腹部CT增强扫描：胰尾囊性占位，考虑囊腺瘤可能，建议必要时结合MRI扫描。腹膜后未见明显肿大淋巴结。

Question: Please determine which syndrome type the patient belongs to:

A. Damp-Heat with Spleen-Deficiency Syndrome

B. Others

C. Spleen-Deficiency Syndrome

D. Damp-Heat Syndrome

**(2) Analysis of Primary and Secondary Symptoms**

- **Feature Extraction:** Identify and extract diagnostically significant symptoms and signs from the case information.

Primary Symptoms: None

Secondary Symptoms: None

- **Mapping Diagnostic Elements**: Map the extracted symptom features to the corresponding diagnostic elements of Damp-Heat Syndrome or Spleen-Deficiency Syndrome (primary symptoms/secondary symptoms).

**Damp-Heat Syndrome:**

None

**Spleen-Deficiency Syndrome:**

None

**(3) Analysis of Tongue and Pulse Features**

- **Identification of Tongue and Pulse Features:** Identify and extract diagnostically significant tongue and pulse characteristics from the case information.

Tongue Features：None

Pulse Features: None

- **Mapping Diagnostic Elements:** Map the extracted tongue and pulse features to the corresponding diagnostic elements of Damp-Heat Syndrome or Spleen-Deficiency Syndrome (primary tongue indicators/additional tongue indicators; primary pulse indicators/additional pulse indicators).
- **Damp-Heat Syndrome:**

None

- **Spleen-Deficiency Syndrome:**

None

**Integrated Analysis and Determination:**

The patient does not meet the diagnostic criteria for either Damp-Heat Syndrome or Spleen-Deficiency Syndrome.

**Conclusion:** The patient is classified as: B. Others.

### Example 4: Spleen-Deficiency Syndrome

**(1) Input of Medical Case Information:**

入院病史: 1.女，50岁；因“胰腺癌肝转移综合治疗3年余”入院；近3月多来患者未行影像学复查，一般情况好，为求进一步治疗收治入院。神清，精神好，食欲不振，睡眠可，二便正常，出院至今消瘦5kg。2.体检：T37°C，P80次/分，R18次/分，BP98/75mmHg，神志清醒，精神安静，发育正常，自动体位，检查合作；皮肤粘膜未见黄染及出血点,无肝掌、蜘蛛痣，无瘀斑、红肿及皮下结节；全身浅表淋巴结未扪及明显增大；头颅外观无畸形，巩膜无黄染、瞳孔等大，对光反应存在，伸舌居中，咽喉无充血，扁桃体大小正常；颈软，颈静脉无怒张，气管居中，甲状腺无肿大；胸廓对称，呼吸均匀、清晰，心律齐，心率80次/分，未闻及病理杂音；腹平软，无压痛及反跳痛，肝肋下未及，剑突下未及，脾肋下未及，亦未扪及明显肿块；直肠指检未及肿块，外生殖器未查；脊柱四肢无畸形，关节运动自如；生理反射存在，病理反射未引出。舌淡红，苔白，脉细。

Question: Please determine which syndrome type the patient belongs to:

A. Damp-Heat Syndrome

B. Spleen-Deficiency Syndrome

C. Others

D. Damp-Heat with Spleen-Deficiency Syndrome

**(2) Analysis of Primary and Secondary Symptoms**

- **Feature Extraction:** Identify and extract diagnostically significant symptoms and signs from the case information.

Primary Symptoms: 食欲不振（loss of appetite）

Secondary Symptoms: 食欲不振（small appetite）；出院至今消瘦5kg（emaciation）

- **Mapping Diagnostic Elements**: Map the extracted symptom features to the corresponding diagnostic elements of Damp-Heat Syndrome or Spleen-Deficiency Syndrome (primary symptoms/secondary symptoms).
- **Damp-Heat Syndrome:**

Loss of appetite (matches the primary symptoms of Damp-Heat Syndrome)

**Spleen-Deficiency Syndrome:**

Small appetite (matches the secondary symptoms of Spleen-Deficiency Syndrome)

Emaciation (matches the secondary symptoms of Spleen-Deficiency Syndrome)

**(3) Analysis of Tongue and Pulse Features**

- **Identification of Tongue and Pulse Features:** Identify and extract diagnostically significant tongue and pulse characteristics from the case information.

Tongue Features: 舌淡红 (pale tongue)，苔白 (thin white coating)

Pulse Features: 脉细 (thin pulse)

- **Mapping Diagnostic Elements:** Map the extracted tongue and pulse features to the corresponding diagnostic elements of Damp-Heat Syndrome or Spleen-Deficiency Syndrome (primary tongue indicators/additional tongue indicators; primary pulse indicators/additional pulse indicators).
- **Damp-Heat Syndrome:**

None

- **Spleen-Deficiency Syndrome:**

Pale tongue (matches the additional tongue indicators of Spleen-Deficiency Syndrome)

Thin white coating (matches the additional tongue indicators of Spleen-Deficiency Syndrome)

Thin pulse (符合脾虚气滞证或见脉)

**Integrated Analysis and Determination:**

The patient exhibits one primary symptoms of Damp-Heat Syndrome (loss of appetite) , which do not meet the diagnostic criteria for Damp-Heat Syndrome.

The patient also exhibits two secondary symptoms of Spleen-Deficiency Syndrome (small appetite, emaciation) and two additional tongue indicators (pale tongue, thin white coating), meeting the diagnostic criteria for Spleen-Deficiency Syndrome.

**Conclusion:** The patient is classified as: B. Spleen-Deficiency Syndrome

### Example 5: Damp-Heat with Spleen-Deficiency Syndrome

**(1) Input of Medical Case Information:**

患者，女，47岁，因“上腹疼痛4月余，发现胰腺及肝占位半月”入院，今为求继续治疗入住我院，现患者一般情况可，上腹部隐痛不适，乏力，无发热、恶心、呕吐，纳差，大便正常，小便黄，无消瘦。2.体检：神志清醒，精神安静，营养中等，发育正常，自动体位，检查合作。皮肤及巩膜中度黄染，浅表淋巴结未扪及明显增大。头部外观无畸形，瞳孔等大，对光反应存在，伸舌居中，颈软，颈静脉无怒张，气管居中，胸廓对称，双肺呼吸音清，未闻及干湿罗音。心律齐，未闻及病理杂音。腹平软,中上腹部压痛，无反跳痛，肝脾未及，移动性浊音（+）。肠鸣音4次/分，外生殖器未查。脊柱四肢无畸形，关节运动自如。双下肢无凹陷性水肿。生理反射存在，病理反射未引出。患者舌质偏红**，**苔白厚**，脉细弦**。

Question: Please determine which syndrome type the patient belongs to:

A. Spleen-Deficiency Syndrome

B. Others

C. Damp-Heat Syndrome

D. Damp-Heat with Spleen-Deficiency Syndrome

**(2) Analysis of Primary and Secondary Symptoms**

- **Feature Extraction:** Identify and extract diagnostically significant symptoms and signs from the case information.

主症：上腹部隐痛不适 (abdominal pain); 乏力 (fatigue); 纳差 (loss of appetite); 小便黄(yellow urine); 皮肤及巩膜中度黄染 (yellowish skin); 皮肤及巩膜中度黄 (yellow sclera)

次症：移动性浊音 (+) (ascites）；双下肢无凹陷性水肿 (edema in lower limbs)

- **Mapping Diagnostic Elements**: Map the extracted symptom features to the corresponding diagnostic elements of Damp-Heat Syndrome or Spleen-Deficiency Syndrome (primary symptoms/secondary symptoms).

**Damp-Heat Syndrome:**

Abdominal pain (matches the primary symptoms of Damp-Heat Syndrome)

Loss of appetite (matches the primary symptoms of Damp-Heat Syndrome)

Yellow urine (matches the primary symptoms of Damp-Heat Syndrome)

Yellowish skin (matches the primary symptoms of Damp-Heat Syndrome)

Yellow sclera (matches the primary symptoms of Damp-Heat Syndrome)

**Spleen-Deficiency Syndrome:**

Fatigue（matches the primary symptoms of Spleen-Deficiency Syndrome）

Ascites（matches the secondary symptoms of Spleen-Deficiency Syndrome）

Edema in lower limbs（matches the secondary symptoms of Spleen-Deficiency Syndrome）

**(3) Analysis of Tongue and Pulse Features**

- **Identification of Tongue and Pulse Features:** Identify and extract diagnostically significant tongue and pulse characteristics from the case information.

Tongue Features：苔白厚 (white slippery coating)

Pulse Features:脉细弦 (wiry, rapid pulse), 脉细弦 (thin wiry pulse)

- **Mapping Diagnostic Elements:** Map the extracted tongue and pulse features to the corresponding diagnostic elements of Damp-Heat Syndrome or Spleen-Deficiency Syndrome (primary tongue indicators/additional tongue indicators; primary pulse indicators/additional pulse indicators).
- **Damp-Heat Syndrome:**

Wiry, rapid pulse (matches the primary pulse indicators of Damp-Heat Syndrome)

- **Spleen-Deficiency Syndrome:**

White slippery coating (matches the additional tongue indicators of Spleen-Deficiency Syndrome)

Thin wiry pulse（matches the additional pulse indicators of Spleen-Deficiency Syndrome）

**Integrated Analysis and Determination:**

The patient exhibits five primary symptoms of Damp-Heat Syndrome (abdominal pain, loss of appetite, yellow urine, yellowish skin, yellow sclera), and one primary pulse feature (wiry, rapid pulse), meeting the diagnostic criteria for Damp-Heat Syndrome.

The patient also exhibits one primary symptoms of Spleen-Deficiency Syndrome (fatigue), two secondary symptoms (ascites, edema in lower limbs) one additional tongue feature (white slippery coating), one additional pulse feature (thin wiry pulse), meeting the diagnostic criteria for Spleen-Deficiency Syndrome.

**Conclusion:** The patient is classified as: D. Damp-Heat with Spleen-Deficiency Syndrome.

### Example 6: Damp-Heat Syndrome

**(1) Input of Medical Case Information:**

女，67岁，因“胰腺癌肝转移近5月，综合治疗后1月”入院。为进一步治疗，门诊拟“胰腺癌肝转移”收入病房。患者入院时无发热，自觉右上腹闷胀不适，腰背部酸痛，纳差，夜寐欠安，二便可，体重较上次出院明显减轻。体格检查：KPS80，神清，锁骨上淋巴结未及肿大，皮肤巩膜轻度黄染。腹平软，无压痛及反跳痛，右侧肋缘下2cm可触及肿块，无触痛，肝剑突下未及，脾肋下未及，肠鸣音3-4次/分，移动性浊音（-），双下肢无水肿。辅助检查：细胞病理 本院 （日期：2012.04.09）编号：Li2012-00073，(肝穿)见恶性肿瘤细胞，倾向腺癌。CT我院(日期:2012.07.24) 编号: 10488723,胰腺癌复查，病灶同前相仿。累及肠系膜静脉。肝内多发转移，部分较前增大。胆囊结石同前，双肾囊肿。患者舌红，苔黄腻，脉细弦

Question: Please determine which syndrome type the patient belongs to:

A. Others

B. Spleen-Deficiency Syndrome

C. Damp-Heat with Spleen-Deficiency Syndrome

D. Damp-Heat Syndrome

**(2) Analysis of Primary and Secondary Symptoms**

- **Feature Extraction:** Identify and extract diagnostically significant symptoms and signs from the case information.

Primary Symptoms: 右上腹闷胀不适 (abdominal bloating); 纳差 (loss of appetite);皮肤巩膜轻度黄染 (yellowish skin); 皮肤巩膜轻度黄染 (yellow sclera)

Secondary Symptoms: 纳差 (small appetite); 体重较上次出院明显减轻 (emaciation)

- **Mapping Diagnostic Elements**: Map the extracted symptom features to the corresponding diagnostic elements of Damp-Heat Syndrome or Spleen-Deficiency Syndrome (primary symptoms/secondary symptoms).

**Damp-Heat Syndrome:**

Abdominal bloating (matches the primary symptoms of Damp-Heat Syndrome)

Loss of appetite (matches the primary symptoms of Damp-Heat Syndrome)

Yellowish skin (matches the primary symptoms of Damp-Heat Syndrome)

Yellow sclera (matches the primary symptoms of Damp-Heat Syndrome)

**Spleen-Deficiency Syndrome:**

Small appetite (matches the secondary symptoms of Spleen-Deficiency Syndrome)

Emaciation (matches the secondary symptoms of Spleen-Deficiency Syndrome)

**(3) Analysis of Tongue and Pulse Features**

- **Identification of Tongue and Pulse Features:** Identify and extract diagnostically significant tongue and pulse characteristics from the case information.

Tongue Features：舌红 (red tongue): 苔黄腻 (yellow greasy coating)

脉象：脉弦 (wiry, rapid pulse); 脉弦 (thin wiry pulse)

- **Mapping Diagnostic Elements:** Map the extracted tongue and pulse features to the corresponding diagnostic elements of Damp-Heat Syndrome or Spleen-Deficiency Syndrome (primary tongue indicators/additional tongue indicators; primary pulse indicators/additional pulse indicators).
- **Damp-Heat Syndrome:**

Wiry, rapid pulse (matches the primary pulse indicators of Damp-Heat Syndrome）

Red tongue (matches the additional tongue indicators of Damp-Heat Syndrome)

Yellow greasy coating (matches the primary tongue indicators of Damp-Heat Syndrome)

- **Spleen-Deficiency Syndrome:**

Thin wiry pulse (matches the additional pulse indicators of Spleen-Deficiency Syndrome)

**Integrated Analysis and Determination:**

The patient exhibits four primary symptoms of Damp-Heat Syndrome (abdominal bloating, loss of appetite, yellowish skin, yellow sclera), one primary tongue feature (yellow, greasy coating), one additional tongue feature (red tongue), and one primary pulse feature (wiry, rapid pulse), meeting the diagnostic criteria for Damp-Heat Syndrome.

The patient also exhibits two secondary symptoms of Spleen-Deficiency Syndrome (small appetite, emaciation) and one additional pulse feature (thin wiry pulse), which do not meet the diagnostic criteria for Spleen-Deficiency Syndrome.

**Conclusion:** The patient is classified as: D. Damp-Heat Syndrome.

### Example 7: Spleen-Deficiency Syndrome

**(1) Input of Medical Case Information:**

患者，女，60岁，因“胰腺癌二次化疗后25天”入院。入院时：乏力，时有恶心，无呕吐，时有反酸，无发热，食欲差，大小便如常，体重减轻20斤。查体：KPS90分，皮肤巩膜未见黄染，锁骨上淋巴结未及肿大。腹平软，无压痛及反跳痛。肝肋下未及，剑突下未及，脾肋下未及，亦未扪及明显肿块，移动性浊音（-），双下肢无肿。辅助检查：3月29日南桥中心医院CT检查提示：胰腺占位，考虑胰腺癌超声检查: 本院 (日期:2012.07.13) 胰腺尾部实质占位（MT可能）肝脏,脾脏,胆囊,腹腔,腹膜后,两侧肾脏,两侧肾上腺未见明显占位门静脉未见异常腹水（-）患者舌淡红，**苔白腻，脉弦细**。

Question: Please determine which syndrome type the patient belongs to:

A. Damp-Heat Syndrome

B. Damp-Heat with Spleen-Deficiency Syndrome

C. Spleen-Deficiency Syndrome

D. Others

**(2) Analysis of Primary and Secondary Symptoms**

- **Feature Extraction:** Identify and extract diagnostically significant symptoms and signs from the case information.

Primary Symptoms: 乏力 (fatigue); 食欲差 (loss of appetite)

Secondary Symptoms: 恶心 (nausea); 食欲差 (small appetite); 体重减轻20公斤 (emaciation)

- **Mapping Diagnostic Elements**: Map the extracted symptom features to the corresponding diagnostic elements of Damp-Heat Syndrome or Spleen-Deficiency Syndrome (primary symptoms/secondary symptoms).
- **Damp-Heat Syndrome:**

Loss of appetite (matches the primary symptoms of Damp-Heat Syndrome)

- **Spleen-Deficiency Syndrome:**

Fatigue (matches the primary symptoms of Spleen-Deficiency Syndrome)

Small appetite (matches the secondary symptoms of Spleen-Deficiency Syndrome)

Emaciation (matches the secondary symptoms of Spleen-Deficiency Syndrome)

**(3) Analysis of Tongue and Pulse Features**

- **Identification of Tongue and Pulse Features:** Identify and extract diagnostically significant tongue and pulse characteristics from the case information.

Tongue Features: 苔白腻 (white slippery coating)

Pulse Features: 脉弦细 (wiry, rapid pulse); 脉弦细 (thin wiry pulse)

- **Mapping Diagnostic Elements:** Map the extracted tongue and pulse features to the corresponding diagnostic elements of Damp-Heat Syndrome or Spleen-Deficiency Syndrome (primary tongue indicators/additional tongue indicators; primary pulse indicators/additional pulse indicators).
- **Damp-Heat Syndrome:**

Wiry, rapid pulse (matches the primary pulse indicators of Damp-Heat Syndrome)

- **Spleen-Deficiency Syndrome:**

White slippery coating (matches the additional tongue indicators of Damp-Heat Syndrome)

Thin wiry pulse (matches the additional pulse indicators of Spleen-Deficiency Syndrome)

**Integrated Analysis and Determination:**

患者表现出湿热蕴结证主症一项（食欲不振），主脉一项（脉弦数），不满足湿热蕴结证辨证标准。

患者表现出脾虚气滞证主症一项（乏力），次症两项（食少，消瘦），或见舌一项（苔白滑），或见脉一项（脉细弦），满足脾虚气滞证辨证标准。

The patient exhibits one primary symptoms of Damp-Heat Syndrome (loss of appetite), one primary pulse feature (wiry, rapid pulse), which do not meet the diagnostic criteria for Damp-Heat Syndrome.

The patient also exhibits one primary symptoms (fatigue) of Spleen-Deficiency Syndrome (small appetite, ascites), two secondary symptoms(small appetite, emaciation), one additional tongue feature (white slippery coating), meeting the diagnostic criteria for Spleen-Deficiency Syndrome.

**Conclusion:** The patient is classified as: C. Spleen-Deficiency Syndrome.

### Example 8: Others

**(1) Input of Medical Case Information:**

患者，男，53y。因“胰体尾癌术后3月，1次化疗后 ”入院。现病史 患者2010-06-04全麻下行胰体癌根治术，手术顺利，术后予以抗炎、保肝、抑酸、抑酶等治疗后，恢复可，术后病理为：高中分化导管腺癌。术后恢复可。2010.7.28局麻下行DSA介入化疗，方案“泽菲1.4+氟尿苷1.0+艾恒150mg”，术顺。现患者为行第二次化疗入院。现患者神清一般可，进食及两便无殊，体重无明显减轻。2.体检：见陈旧手术疤痕。腹平软，无压痛及反跳痛。肝肋下未及，剑突下未及，脾肋下未及。亦未扪及明显肿块。3.辅检：暂缺。病史小结: 1.患者，男，53y。2.因“胰体尾癌术后3月，1次化疗后 ”入院。3.重要体检结果：见陈旧手术疤痕。腹平软，无压痛及反跳痛。肝肋下未及，剑突下未及，脾肋下未及。亦未扪及明显肿块。4.重要辅助检查结果：暂缺。讨论: 根据病史及体检诊断基本明确诊疗计划: 1.完善相关检查 2.按期化疗临床诊断: 胰体尾癌术后

Question: Please determine which syndrome type the patient belongs to:

A. Damp-Heat with Spleen-Deficiency Syndrome

B. Spleen-Deficiency Syndrome

C. Damp-Heat Syndrome

D. Others

**(2) Analysis of Primary and Secondary Symptoms**

- **Feature Extraction:** Identify and extract diagnostically significant symptoms and signs from the case information.

Primary Symptoms: None

Secondary Symptoms: None

- **Mapping Diagnostic Elements**: Map the extracted symptom features to the corresponding diagnostic elements of Damp-Heat Syndrome or Spleen-Deficiency Syndrome (primary symptoms/secondary symptoms).

**Damp-Heat Syndrome:**

None

**Spleen-Deficiency Syndrome:**

None

**(3) Analysis of Tongue and Pulse Features**

- **Identification of Tongue and Pulse Features:** Identify and extract diagnostically significant tongue and pulse characteristics from the case information.

Tongue Features：None

Pulse Features: None

- **Mapping Diagnostic Elements:** Map the extracted tongue and pulse features to the corresponding diagnostic elements of Damp-Heat Syndrome or Spleen-Deficiency Syndrome (primary tongue indicators/additional tongue indicators; primary pulse indicators/additional pulse indicators).
- **Damp-Heat Syndrome:**

None

- **Spleen-Deficiency Syndrome:**

None

**Integrated Analysis and Determination:**

The patient does not meet the diagnostic criteria for either Damp-Heat Syndrome or Spleen-Deficiency Syndrome.

**Conclusion:** The patient is classified as: D. Others.

### Test Cases for LLMs with English Background

### Test 1: Damp-Heat with Spleen-Deficiency Syndrome

**Please integrate the concepts of “Role-Play Prompting,” “In-Context Learning and Chain-of-Thought,” discussed earlier, along with the content and format of the eight subsequent task examples, to analyze and diagnose the case presented below.**

**(1) Input of Medical Case Information:**

患者，女，71岁； 2. 因“诊断胰腺癌4月余，梗阻性黄疸半月余”；为求进一步治疗收治我院，入院时患者**左下腹疼痛不适**，**精神欠佳**，**纳少**，夜寐安，二便可，**近期体重出现明显减轻**。4.体格检查:KPS80，神清，**乏力**貌，**皮肤巩膜黄染**，锁骨上淋巴结未及明显肿大。腹部正中见长约10cm纵行手术疤痕，愈合可。两肺呼吸音粗，未闻及罗音。腹平软，腹部无压痛，未及反跳痛，肝肋下未及，剑突下未及，脾肋下未及，移动性浊音（-），双下肢无水肿。5. 辅助检查：2015.09.22上海市第六人民医院腹部CT：1. 胰腺头颈部密度减低，肝总动脉管壁不规则，胰腺尾部萎缩，周围小淋巴结，考虑胰腺癌可能，胆囊切除术后改变。2015.09.25上海市第六人民医院腹部MR：胰腺头颈部异常信号伴浸润临近腹腔干，考虑胰腺癌可能大。2016.02.25本院MRCP：低位胆道梗阻，梗阻位于胆总管上段可能，胰头后方占位，请结合腹部常规影像学检查，**腹腔少量积液**。患者**舌红**，**苔薄黄**，**脉细弦**

Question: Please determine which syndrome type the patient belongs to:

A. Damp-Heat with Spleen-Deficiency Syndrome

B. Others

C. Damp-Heat Syndrome

D. Spleen-Deficiency Syndrome

**(2) Analysis of Primary and Secondary Symptoms**

**(3) Analysis of Tongue and Pulse Features**

**Integrated Analysis and Determination:**

### Test 2: Damp-Heat Syndrome

**Please integrate the concepts of “Role-Play Prompting,” “In-Context Learning and Chain-of-Thought,” discussed earlier, along with the content and format of the eight subsequent task examples, to analyze and diagnose the case presented below.**

**(1) Input of Medical Case Information:**

男，52岁，因“胰腺癌肝转移术后2年余，综合治疗后2月”入院。为进一步治疗，门诊拟“胰腺癌肝转移术后”收入病房。患者入院时中上腹隐痛不适，无发热，皮肤巩膜黄染，纳差，二便调，夜寐安，体重较上次入院时未见减轻。体格检查：KPS90，神清，皮肤巩膜轻度黄染，锁骨上淋巴结未及肿大。腹平软，腹部见陈旧性手术疤痕，愈合可，中上腹压痛（+），无反跳痛，肝肋下未及，剑突下未及，脾肋下未及，肠鸣音3-4次/分，移动性浊音（-），双下肢无肿。辅助检查：组织学检查：本院（日期：2010.12.28）病理号：2010-24028，（胰体尾）中分化腺癌，部分神经内分泌分化，癌肿大小3.5*2.4*1cm，神经侵犯（+），脉管癌栓（-）。胰腺周围淋巴结转移（0/5）。（右肝部分）转移性中分化腺癌。转移灶大小5.5*4.8*4.4cm，紧贴切缘（<0.5cm）。脾脏组织未见癌累及。CT：本院（日期：2013.02.16）编号：10617702，胰尾癌术后肝转移治疗后，胰腺体尾部、脾脏术后缺如。肝多发转移，部分较前增大。右侧横膈前组淋巴结肿大。患者舌红，苔薄黄，脉细弦

Question: Please determine which syndrome type the patient belongs to:

A. Damp-Heat Syndrome

B. Others

C. Damp-Heat with Spleen-Deficiency Syndrome

D. Spleen-Deficiency Syndrome

**(2) Analysis of Primary and Secondary Symptoms**

**(3) Analysis of Tongue and Pulse Features**

**Integrated Analysis and Determination:**

### Test 3: Spleen-Deficiency Syndrome

**Please integrate the concepts of “Role-Play Prompting,” “In-Context Learning and Chain-of-Thought,” discussed earlier, along with the content and format of the eight subsequent task examples, to analyze and diagnose the case presented below.**

**(1) Input of Medical Case Information:**

2015.01.08 16:14 首 次 病 程 记 录 一、病例特点: 1.患者，男，44岁，因“右肝癌术后24月，复发3次介入后2月”入院。现为求进一步治疗再次入院，患者入院时未诉不适，纳差，二便调，夜寐安，近期体重减轻8kg。3.体格检查：KPS：90。皮肤粘膜未见黄染及出血点。无瘀斑、红肿及皮下结节。全身浅表淋巴结未扪及增大。心肺听诊未见异常。腹平软，无压痛及反跳痛。肝肋下未及，剑突下未及，脾肋下未及。亦未扪及肿块。移动性浊音（+），双下肢无凹陷性水肿。4.辅助检查：病理组织检查: 本院 (日期:2012.12.13) 编号: 2012-34543（右肝肿瘤）高分化肝细胞性肝癌（3个），肿瘤大小分别为2.5*2*2cm，2.6*1.8*1.5cm，1.8*2*1.8cm。（2014.12.29）腹部CT提示：肝脏术后改变，肝内少许碘油沉积较前缩小，请随访。肝右叶数枚低密度影较前明显，考虑复发或转移，建议结合临床和MRI检查。肝门区结节同前，随访。左侧腹腔多结节较前增大增多，考虑转移。患者舌淡红苔白腻，脉细弦

Question: Please determine which syndrome type the patient belongs to:

A. Damp-Heat with Spleen-Deficiency Syndrome

B. Damp-Heat Syndrome

C. Others

D. Spleen-Deficiency Syndrome

**(2) Analysis of Primary and Secondary Symptoms**

**(3) Analysis of Tongue and Pulse Features**

**Integrated Analysis and Determination:**

### Test 4: Others

**Please integrate the concepts of “Role-Play Prompting,” “In-Context Learning and Chain-of-Thought,” discussed earlier, along with the content and format of the eight subsequent task examples, to analyze and diagnose the case presented below.**

**(1) Input of Medical Case Information:**

自上次出院来患者一般可，饮食睡眠可，体重无明显减轻。4.ECOG1分,神清，精神可。全身浅表淋巴结未触及肿大，两肺未及明显干湿罗音，正中腹可见陈旧性手术疤痕，愈合良好，腹平软，无压痛及反跳痛，肝脾肋下未及，移动性浊音（-），双下肢无水肿。（二）拟诊讨论: (一)诊断:1.胰体尾癌术后复发，肺、淋巴结转移； 2.高血压病I级；3.II型糖尿病 (二)诊断依据:根据患者病史、体征、影像学及术后病理结果，该诊断可成立。 (三)鉴别诊断: 患者肺部转移病灶尚需鉴别：原发性肺癌：患者早期可无症状，后期可出现咳嗽、痰血、胸痛、胸闷等，影像学上常表现为肺部孤立性结节，边缘可有毛刺，或有胸膜凹陷征等。确诊依靠粗针穿刺或支气管下病理活检等。该患者肺部结节出现在胰腺癌发病后，且呈多发病灶，随胰腺复发病灶一起进展，考虑转移可能性较大。（三）诊疗计划: 完善相关检查，排除靶向治疗禁忌，明日予第5程赛伐珠单抗靶向治疗。

Question: Please determine which syndrome type the patient belongs to:

A. Spleen-Deficiency Syndrome

B. Damp-Heat Syndrome

C. Damp-Heat with Spleen-Deficiency Syndrome

D. Others

**(2) Analysis of Primary and Secondary Symptoms**

**(3) Analysis of Tongue and Pulse Features**

**Integrated Analysis and Determination:**

## The Prompt Framework for Large Language Models in Chinese (for Ernie Bot 4.0 Turbo, Kimi, HuaTuoGPT II, Zhipu Qingyan)

**接下来，我将提供一个“Role-Play Prompting”的设计示例，一个“情境学习和思维链”的示例，以及后续的四个任务实例。请您先阅读并了解这些内容，无需回复。在我分六次完成所有展示并提出具体问题后，再请您根据我的提问进行回答。**

### Role-Play Prompting

您是一位中西医结合肿瘤治疗领域的中医专家，正在为胰腺癌患者进行辨证分析。请根据患者提供的病案内容，结合以下“辨证要素和辨证标准”，判断患者的病情属于以下四种证型中的哪一种：湿热蕴结证、脾虚气滞证、湿热蕴结兼脾虚气滞证或其他。

以下是各证型的详细辨证要素，包括主症、舌象、脉象以及次症等内容：

- **证型：湿热蕴结证**
- **主症：**皮肤黄染；巩膜黄染；小便黄；大便如灰白陶土色；食欲不振；恶心；呕吐；腹胀；腹痛
- **次症：**发热缠绵；口渴而不喜饮；口苦；口臭；便溏味重；心中懊恼
- **主舌：**苔黄；苔黄腻
- **主脉：**脉弦数
- **或见舌：**舌红
- **或见脉：**脉数
- **证型：脾虚气滞证**
- **主症：**神疲；乏力；少气懒言；腰痛绵绵；胸腹胀满食后更甚；大便溏薄；纳呆
- **次症：**食少；消瘦；气短；自汗；畏寒肢冷；上腹部不适或疼痛，按之舒适；面部浮肿；面色白；恶风；口干不多饮；恶心；呕吐；下肢浮肿；腹水；脉濡缓
- **主舌：**舌淡胖
- **主脉：**脉虚
- **或见舌：**舌边齿痕；苔白滑；薄白苔；舌质淡；苔薄；苔薄腻；脉沉细
- **或见脉：**脉细弱；脉沉迟；脉细；脉细弦

**（1）单证型辨证标准：**

符合主症两个，并见主舌/主脉者，即可辨为本证。

符合“主症”两个，或“次症”一个，且符合任何本证的“舌/脉”者，即可辨为本证。

符合“主症”一个，或“次症”不少于两个，且符合任何本证的“舌/脉”者，即可辨为本证。

**（2）兼证型辨证标准：**

如果患者的临床表现同时符合“湿热蕴结证”和“脾虚气滞证”的特征，则判断为湿热蕴结兼脾虚气滞证。

**（3）其他：**

对于未能满足上述任何辨证标准，或病案信息不足以明确归类的患者，将统一归为“其他”。

### 情境学习和思维链（In-Context Learning and Chain-of-Thought）

接下来，我们将通过示例，演示如何根据患者的病案内容逐步分析并判断其证型。在这些示例中，将系统地分析患者的“主症”、“次症”、“主舌”、“或见舌”以及“或见脉”等信息，并将所有相关信息进行整合，结合各证型的辨证标准，最终明确患者的证型。

在以下示例中，每位患者的病案将按以下步骤进行分析：

**（1）输入病案内容**

**（2）主症与次症的分析**

- 提取特征：从病案内容中识别和提取具有辨证意义的症状和体征。
- 映射辨证要素：将提取的症状特征映射至湿热蕴结证或脾虚气滞证对应的辨证要素（主症/次症）。

**（3）舌象与脉象的分析**

- 识别舌象和脉象特征：从病案内容中识别和提取具有辨证意义的舌象和脉象特征。
- 映射辨证要素：将提取的舌象和脉象特征映射至湿热蕴结证或脾虚气滞证对应的辨证要素（主舌/或见舌；主脉/或见脉）。

**（4）整合分析与判断**

- 综合特征：综合主症、次症、舌象及脉象的信息，对比辨证标准，明确患者的全部辨证要素特征。
- 输出辨证结果

### 示例1. 湿热蕴结证

**（1）输入病案内容：**

男，58岁，因“下腹隐痛伴消瘦3月余，发现胰尾占位半月余”入院。今为进一步诊治，入住我科病房，入院时下腹部隐痛，无恶心呕吐，纳差，大便偏干2月余，使用开塞露能够解出，既往无便秘，小便黄，近3个月来体重无明显减轻。体格检查：KPS 90,神清，锁骨上淋巴结未扪及明显增大。腹平软，无压痛及反跳痛。肝肋下未及，剑突下未及，脾肋下未及。肠鸣音无亢，移动性浊音（-），双下肢无水肿。辅助检查：腹部CT（外院，2012.10.19）：1.胰尾、脾门区及肝门部密度异常、结构紊乱；2.肝右后叶下段低密度灶；3.脾脏稍大；4.右肾盂及右输尿管上段扩张积液；5.胆囊显示不清；6.腹部MRI（外院，2012.10.25）：1.胰腺尾部实质性占位，考虑：胰尾癌可能大，伴肝门部、腹膜后区淋巴结转移；2.肝内胆管、胆总管轻度扩张；3.右侧肾盂、输尿管扩张；4.肝右叶、左肾多发小囊肿；5.少量腹腔积液。肿瘤标志物(外院，2012.10.20)：AFP 4.91,CA125 131.2U/ml,CA199 147.80U/ml,CA211 21.26ng/ml,CA724 203.80U/ml。病理（外院，2012.10.26）：（直肠）粘膜慢性炎，局灶腺体萎缩。患者舌红，苔黄腻，脉弦。

请选择患者属于以下哪一种证型？

A. 脾虚气滞证

B. 湿热蕴结证

C. 其他

D. 湿热蕴结兼脾虚气滞证

**（2）主症与次症的分析**

- **提取特征**：从病案内容中识别和提取具有辨证意义的症状和体征。

主症：下腹隐痛（腹痛）；纳差（食欲不振）；小便黄（小便黄）。

次症：纳差（食少）；腹腔积液（腹水）

- **映射辨证要素**：将提取的症状特征映射至湿热蕴结证或脾虚气滞证对应的辨证要素（主症/次症）。

**湿热蕴结证：**

腹痛（符合湿热蕴结证主症）。

小便黄（符合湿热蕴结证主症）。

食欲不振（符合湿热蕴结证主症）。

**脾虚气滞证：**

食少（符合脾虚气滞证次症）

腹水（符合脾虚气滞证次症）

**（3）舌象与脉象的分析**

- **识别舌象和脉象特征**：从病案内容中识别和提取具有辨证意义的舌象和脉象特征。

舌象：舌红（舌红）；苔黄腻（苔黄腻）。

脉象：脉弦（脉弦数）；脉弦（脉细弦）

- **映射辨证要素**：将提取的舌象和脉象特征映射至湿热蕴结证或脾虚气滞证对应的辨证要素（主舌/或见舌；主脉/或见脉）。

**湿热蕴结证：**

苔黄腻（符合湿热蕴结证主舌）。

舌红（符合湿热蕴结证或见舌）

脉弦数（符合湿热蕴结证主脉）

**脾虚气滞证：**

脉细弦（符合脾虚气滞证或见脉）

**整合分析与判断：**

患者表现出湿热蕴结证主症三项（腹痛、食欲不振、小便黄），主舌一项（苔黄腻）、或见舌一项（舌红），主脉一项（脉弦数），满足湿热蕴结证辨证标准。

患者表现出脾虚气滞证次症两项（食少、腹水），或见脉一项（脉细弦）未达到脾虚气滞证的辨证标准。

综上该患者为：B. 湿热蕴结证

### 示例2. 湿热蕴结兼脾虚气滞证

**（1）输入病案内容：**

（1）患者男，58岁。（2）因“胰腺癌盆腔转移2月，介入化疗后1月余”入院。为进一步治疗再次入院。入院时：下腹部痛，纳差，小便色黄，量正常，大便不畅，无发热，无黄疸，出院至今消瘦3公斤。（4）体格检查：KPS 90,神清，锁骨上淋巴结未扪及明显增大。腹平软，下腹部压痛（+），反跳痛（-），肝肋下未及，剑突下未及，脾肋下未及。肠鸣音无亢，移动性浊音（+）。右下肢大腿正面可见一陈旧性手术疤痕，长约50cm,愈合良好，双下肢无凹陷性水肿。患者舌红，苔黄腻，脉弦。

请选择患者属于以下哪一种证型？

A. 湿热蕴结证

B. 脾虚气滞证

C. 湿热蕴结兼脾虚气滞证

D. 其他

**（2）主症与次症的分析**

- **提取特征**：从病案内容中识别和提取具有辨证意义的症状和体征。

主症：下腹部痛，下腹部压痛（+）（腹痛）；纳差（食欲不振）；小便色黄（小便黄）。

次症：纳差（食少）；腹腔积液，移动性浊音（+）（腹水）；出院至今消瘦3公斤（消瘦）；双下肢无凹陷性水肿（下肢浮肿）

- **映射辨证要素**：将提取的症状特征映射至湿热蕴结证或脾虚气滞证对应的辨证要素（主症/次症）。

**湿热蕴结证：**

腹痛（符合湿热蕴结证主症）。

食欲不振（符合湿热蕴结证主症）。

小便黄（符合湿热蕴结证主症）。

**脾虚气滞证：**

食少（符合脾虚气滞证次症）

腹水（符合脾虚气滞证次症）

消瘦（符合脾虚气滞证次症）

下肢浮肿（符合脾虚气滞证次症）

**（3）舌象与脉象的分析**

- **识别舌象和脉象特征**：从病案内容中识别和提取具有辨证意义的舌象和脉象特征。

舌象：舌红（舌红），苔黄腻（苔黄腻）。

脉象：脉弦（脉弦数），脉弦（脉细弦）

- **映射辨证要素**：将提取的舌象和脉象特征映射至湿热蕴结证或脾虚气滞证对应的辨证要素（主舌/或见舌；主脉/或见脉）。

**湿热蕴结证：**

苔黄腻（符合湿热蕴结证主舌）。

舌红（符合湿热蕴结证或见舌）

脉弦数（符合湿热蕴结证主脉）

**脾虚气滞证：**

脉细弦（符合脾虚气滞证或见脉）

**整合分析与判断：**

患者表现出湿热蕴结证主症三项（腹痛、小便黄、食欲不振），主舌一项（苔黄腻）、或见舌一项（舌红），主脉一项（脉弦数），满足湿热蕴结证辨证标准。

患者表现出脾虚气滞证次症四项（食少、腹水、消瘦、下肢浮肿），或见脉一项（脉细弦），满足脾虚气滞证辨证标准。

综上该患者为：C. 湿热蕴结兼脾虚气滞证

### 示例3. 其他

**（1）输入病案内容：**

一、病例特点: 患者1月前无明显诱因下自觉上腹胀痛，外院查体提示胰体尾占位。患者无明显自觉症状，无发热、腹痛、恶心呕吐，无皮肤巩膜黄染，无腹胀腹泻。为进一步治疗来我院。门诊以“胰腺肿瘤”收治入院。患者病来一般情况良好，神志清、精神可，大小便基本正常。二、拟诊讨论: (一)诊断:胰腺肿瘤 (二)诊断依据:1.患者，女，26岁2.因“上腹胀痛一月余”入院3.体检：ECOG：1，神志清，查体合作，全身浅表淋巴结未及肿大，皮肤巩膜无黄染，腹平软。右上腹深压痛阴性，无明显肌紧张、反跳痛。余腹部无明显压痛及反跳痛。肝肋下未及，剑突下未及，脾肋下未及。亦未扪及明显肿块。Murphy征阴性，移动性浊音阴性，肠鸣音3-5次/分4.辅助检查：CT:(中山医院，2015-10-21)诊断: 腹部CT增强扫描：胰尾囊性占位，考虑囊腺瘤可能，建议必要时结合MRI扫描。腹膜后未见明显肿大淋巴结。

请选择患者属于以下哪一种证型？

A. 湿热蕴结兼脾虚气滞证

B. 其他

C. 脾虚气滞证

D. 湿热蕴结证

**（2）主症与次症的分析**

- **提取特征**：从病案内容中识别和提取具有辨证意义的症状和体征。

主症：无

次症：无

- **映射辨证要素**：将提取的症状特征映射至湿热蕴结证或脾虚气滞证对应的辨证要素（主症/次症）。

**湿热蕴结证：**

无

**脾虚气滞证：**

无

**（3）舌象与脉象的分析**

- **识别舌象和脉象特征**：从病案内容中识别和提取具有辨证意义的舌象和脉象特征。

舌象：无

脉象：无

- **映射辨证要素**：将提取的舌象和脉象特征映射至湿热蕴结证或脾虚气滞证对应的辨证要素（主舌/或见舌；主脉/或见脉）。

**湿热蕴结证：**

无

**脾虚气滞证：**

无

**整合分析与判断：**

患者未达到湿热蕴结证或脾虚气滞证的辨证标准。

综上该患者为：B. 其他

### 示例4. 脾虚气滞证

**（1）输入病案内容：**

入院病史: 1.女，50岁；因“胰腺癌肝转移综合治疗3年余”入院；近3月多来患者未行影像学复查，一般情况好，为求进一步治疗收治入院。神清，精神好，食欲不振，睡眠可，二便正常，出院至今消瘦5kg。2.体检：P80次/分，R18次/分，BP98/75mmHg，神志清醒，精神安静，发育正常，自动体位，检查合作；皮肤粘膜未见黄染及出血点,无肝掌、蜘蛛痣，无瘀斑、红肿及皮下结节；全身浅表淋巴结未扪及明显增大；头颅外观无畸形，巩膜无黄染、瞳孔等大，对光反应存在，伸舌居中，咽喉无充血，扁桃体大小正常；颈软，颈静脉无怒张，气管居中，甲状腺无肿大；胸廓对称，呼吸均匀、清晰，心律齐，心率80次/分，未闻及病理杂音；腹平软，无压痛及反跳痛，肝肋下未及，剑突下未及，脾肋下未及，亦未扪及明显肿块；直肠指检未及肿块，外生殖器未查；脊柱四肢无畸形，关节运动自如；生理反射存在，病理反射未引出。舌淡红，苔白，脉细。

请选择患者属于以下哪一种证型？

A. 湿热蕴结证

B. 脾虚气滞证

C. 其他

D. 湿热蕴结兼脾虚气滞证

**（2）主症与次症的分析**

- **提取特征**：从病案内容中识别和提取具有辨证意义的症状和体征。

主症：食欲不振（食欲不振）

次症：食欲不振（食少）；出院至今消瘦5kg（消瘦）

- **映射辨证要素**：将提取的症状特征映射至湿热蕴结证或脾虚气滞证对应的辨证要素（主症/次症）。

**湿热蕴结证：**

食欲不振（符合湿热蕴结证主症）。

**脾虚气滞证：**

食少（符合脾虚气滞证次症）

消瘦（符合脾虚气滞证次症）

**（3）舌象与脉象的分析**

- **识别舌象和脉象特征**：从病案内容中识别和提取具有辨证意义的舌象和脉象特征。

舌象：舌淡红（舌质淡），苔白（薄白苔）。

脉象：脉细（脉细）

- **映射辨证要素**：将提取的舌象和脉象特征映射至湿热蕴结证或脾虚气滞证对应的辨证要素（主舌/或见舌；主脉/或见脉）。

**湿热蕴结证：**

无

**脾虚气滞证：**

舌质淡（符合脾虚气滞证或见舌）

苔白（符合脾虚气滞证或见舌）

脉细（符合脾虚气滞证或见脉）

**整合分析与判断：**

患者表现出湿热蕴结证主症一项（食欲不振），不满足湿热蕴结证辨证标准。

患者表现出脾虚气滞证次症两项（食少、消瘦），或见舌两项（舌质淡、苔白），或见脉一项（脉细），满足脾虚气滞证辨证标准。

综上该患者为：B. 脾虚气滞证

### Test Cases for LLMs with Chinese Background

### 测试病案1（湿热蕴结兼脾虚气滞证）

**请您结合前面输入的“Role-Play Prompting”、“情境学习和思维链”，以及后续的四个任务实例中的内容和格式，对下面输入的病案进行辨证。**

**（1）输入病案内容：**

患者，女，71岁； 2. 因“诊断胰腺癌4月余，梗阻性黄疸半月余”；为求进一步治疗收治我院，入院时患者**左下腹疼痛不适**，**精神欠佳**，**纳少**，夜寐安，二便可，**近期体重出现明显减轻**。4.体格检查:KPS80，神清，**乏力**貌，**皮肤巩膜黄染**，锁骨上淋巴结未及明显肿大。腹部正中见长约10cm纵行手术疤痕，愈合可。两肺呼吸音粗，未闻及罗音。腹平软，腹部无压痛，未及反跳痛，肝肋下未及，剑突下未及，脾肋下未及，移动性浊音（-），双下肢无水肿。5. 辅助检查：2015.09.22上海市第六人民医院腹部CT：1. 胰腺头颈部密度减低，肝总动脉管壁不规则，胰腺尾部萎缩，周围小淋巴结，考虑胰腺癌可能，胆囊切除术后改变。2015.09.25上海市第六人民医院腹部MR：胰腺头颈部异常信号伴浸润临近腹腔干，考虑胰腺癌可能大。2016.02.25本院MRCP：低位胆道梗阻，梗阻位于胆总管上段可能，胰头后方占位，请结合腹部常规影像学检查，**腹腔少量积液**。患者**舌红**，**苔薄黄**，**脉细弦**

请选择患者属于以下哪一种证型？

A. 湿热蕴结兼脾虚气滞证

B. 其他

C. 湿热蕴结证

D. 脾虚气滞证

**（2）主症与次症的分析**

**（3）舌象与脉象的分析**

**整合分析与判断：**

### 测试案例2（湿热蕴结证）

**请您结合前面输入的“Role-Play Prompting”、“情境学习和思维链”，以及后续的四个任务实例中的内容和格式，对下面输入的病案进行辨证。**

**（1）输入病案内容：**

男，52岁，因“胰腺癌肝转移术后2年余，综合治疗后2月”入院。为进一步治疗，门诊拟“胰腺癌肝转移术后”收入病房。患者入院时中上腹隐痛不适，无发热，皮肤巩膜黄染，纳差，二便调，夜寐安，体重较上次入院时未见减轻。体格检查：KPS90，神清，皮肤巩膜轻度黄染，锁骨上淋巴结未及肿大。腹平软，腹部见陈旧性手术疤痕，愈合可，中上腹压痛（+），无反跳痛，肝肋下未及，剑突下未及，脾肋下未及，肠鸣音3-4次/分，移动性浊音（-），双下肢无肿。辅助检查：组织学检查：本院（日期：2010.12.28）病理号：2010-24028，（胰体尾）中分化腺癌，部分神经内分泌分化，癌肿大小3.5*2.4*1cm，神经侵犯（+），脉管癌栓（-）。胰腺周围淋巴结转移（0/5）。（右肝部分）转移性中分化腺癌。转移灶大小5.5*4.8*4.4cm，紧贴切缘（<0.5cm）。脾脏组织未见癌累及。CT：本院（日期：2013.02.16）编号：10617702，胰尾癌术后肝转移治疗后，胰腺体尾部、脾脏术后缺如。肝多发转移，部分较前增大。右侧横膈前组淋巴结肿大。患者舌红，苔薄黄，脉细弦

请选择患者属于以下哪一种证型？

A. 湿热蕴结兼脾虚气滞证

B. 其他

C. 湿热蕴结证

D. 脾虚气滞证

**（2）主症与次症的分析**

**（3）舌象与脉象的分析**

**整合分析与判断：**

### 测试案例3（脾虚气滞证）

**请您结合前面输入的“Role-Play Prompting”、“情境学习和思维链”，以及后续的四个任务实例中的内容和格式，对下面输入的病案进行辨证。**

**（1）输入病案内容：**

2015.01.08 16:14 首 次 病 程 记 录 一、病例特点: 1.患者，男，44岁，因“右肝癌术后24月，复发3次介入后2月”入院。现为求进一步治疗再次入院，患者入院时未诉不适，纳差，二便调，夜寐安，近期体重减轻8kg。3.体格检查：KPS：90。皮肤粘膜未见黄染及出血点。无瘀斑、红肿及皮下结节。全身浅表淋巴结未扪及增大。心肺听诊未见异常。腹平软，无压痛及反跳痛。肝肋下未及，剑突下未及，脾肋下未及。亦未扪及肿块。移动性浊音（+），双下肢无凹陷性水肿。4.辅助检查：病理组织检查: 本院 (日期:2012.12.13) 编号: 2012-34543（右肝肿瘤）高分化肝细胞性肝癌（3个），肿瘤大小分别为2.5*2*2cm，2.6*1.8*1.5cm，1.8*2*1.8cm。（2014.12.29）腹部CT提示：肝脏术后改变，肝内少许碘油沉积较前缩小，请随访。肝右叶数枚低密度影较前明显，考虑复发或转移，建议结合临床和MRI检查。肝门区结节同前，随访。左侧腹腔多结节较前增大增多，考虑转移。患者舌淡红苔白腻，脉细弦

**（2）主症与次症的分析**

**（3）舌象与脉象的分析**

**整合分析与判断：**

### 测试案例4（其他）

**请您结合前面输入的“Role-Play Prompting”、“情境学习和思维链”，以及后续的四个任务实例中的内容和格式，对下面输入的病案进行辨证。**

**（1）输入病案内容：**

自上次出院来患者一般可，饮食睡眠可，体重无明显减轻。4.ECOG1分,神清，精神可。全身浅表淋巴结未触及肿大，两肺未及明显干湿罗音，正中腹可见陈旧性手术疤痕，愈合良好，腹平软，无压痛及反跳痛，肝脾肋下未及，移动性浊音（-），双下肢无水肿。（二）拟诊讨论: (一)诊断:1.胰体尾癌术后复发，肺、淋巴结转移； 2.高血压病I级；3.II型糖尿病 (二)诊断依据:根据患者病史、体征、影像学及术后病理结果，该诊断可成立。 (三)鉴别诊断: 患者肺部转移病灶尚需鉴别：原发性肺癌：患者早期可无症状，后期可出现咳嗽、痰血、胸痛、胸闷等，影像学上常表现为肺部孤立性结节，边缘可有毛刺，或有胸膜凹陷征等。确诊依靠粗针穿刺或支气管下病理活检等。该患者肺部结节出现在胰腺癌发病后，且呈多发病灶，随胰腺复发病灶一起进展，考虑转移可能性较大。（三）诊疗计划: 完善相关检查，排除靶向治疗禁忌，明日予第5程赛伐珠单抗靶向治疗。

**（2）主症与次症的分析**

**（3）舌象与脉象的分析**

**整合分析与判断：**
