# Supplementary material for: Identification of Syndrome Types in Patients With Pancreatic Cancer From Free Text in Electronic Medical Records: Model Development and Validation
Source: JMIR Form Res. 2025 Oct 3;9:e70602. doi: 10.2196/70602 (PMC12534766; doi:10.2196/70602)
Supplement: Multimedia Appendix 8 [file formative_v9i1e70602_app8.docx]

**Supplementary Table 4. Contingency table for inter-rater agreement between two TCM experts in the syndrome differentiation task.**

| Expert A \ Expert B | damp-heat syndrome (1) | spleen-deficiency syndrome (2) | damp-heat with spleen-deficiency syndrome (3) | Others (4) | Row total |
| --- | --- | --- | --- | --- | --- |
| damp-heat syndrome (1) | 1592 | 32 | 19 | 38 | 1681 |
| spleen-deficiency syndrome (2) | 20 | 1108 | 32 | 41 | 1201 |
| damp-heat with spleen-deficiency syndrome (3) | 28 | 21 | 1088 | 30 | 1167 |
| Others (4) | 49 | 65 | 48 | 2619 | 2781 |
| Column total | 1689 | 1226 | 1187 | 2728 | 6830 |

**Supplementary Table 5. Optimal training parameters for each model after tuning.**

| **Parameter** | **TCMPCSD-BERT^a^** | **LSTM^b^** | **Text-CNN^c^** |
| --- | --- | --- | --- |
| **Learning Rate** | 2e-5 | 1e-3 | 1e-3 |
| **Epochs (iteration)** | 80 | 100 | 200 |
| **Batch Size** | 8 | 16 | 16 |
| **Kernel Size** | NA^d^ | NA^d^ | 2, 3, 4, 5 |
| **Dropout** | 0.3 | 0.5 | NA^d^ |
| **Optimizer** | Adam | Adam | Adam |

^a^TCMPCSD-BERT: Traditional Chinese medicine Pancreatic Cancer Syndrome Differentiation Bidirectional Encoder Representations from Transformers.

^b^LSTM: Long Short-Term Memory.

^c^Text-CNN: Text Convolutional Neural Network.

^d^NA: not applicable.
