# Supplementary material for: Identification of Syndrome Types in Patients With Pancreatic Cancer From Free Text in Electronic Medical Records: Model Development and Validation
Source: JMIR Form Res. 2025 Oct 3;9:e70602. doi: 10.2196/70602 (PMC12534766; doi:10.2196/70602)
Supplement: Multimedia Appendix 9 [file formative_v9i1e70602_app9.docx]

**Supplementary Table 6. Correspondence between clinical case features and TCM guidelines with differentiation significance.**

|  |  | **Damp-heat Syndrome** | | **Spleen-deficiency Syndrome** | |
| --- | --- | --- | --- | --- | --- |
| **Original Case Record** | **Translated Clinical Case Records** | **Corresponding Guideline Content** | **Clinical Significance for Syndrome Differentiation** | **Corresponding Guideline Content** | **Clinical Significance for Syndrome Differentiation** |
| 右上腹隐痛 | dull pain in the upper right abdomen | abdominal pain | primary symptom | -^b^ | NA^c^ |
| 纳差 | poor appetite | loss of appetite | primary symptom | small appetite | secondary symptom |
| 小便色黄 | yellowish urine | yellow urine | primary symptom | - ^b^ | NA^c^ |
| 巩膜黄染 | The sclera showed jaundice | yellow sclera | primary symptom | - ^b^ | NA^c^ |
| 舌红 | red tongue | red tongue | secondary symptom^a^ | - ^b^ | NA^c^ |
| 苔黄腻 | yellow greasy  coating | yellow greasy coating | primary symptom^a^ | - ^b^ | NA^c^ |
| 脉弦滑 | pulse was wiry and slippery | wiry, rapid pulse | primary symptom^a^ | - ^b^ | NA^c^ |
| 患者出现腹泻，十余次每天，稀水样便 | she experienced diarrhea, with more than  ten times per day characterized by watery stools | - ^b^ | NA^c^ | loose stools | primary symptom |
| 纳食一般 | Appetite was slightly reduced | loss of appetite | primary symptom | small appetite | secondary symptom |
| 体重明显下降 | significant weight loss was noted | - ^b^ | NA^c^ | emaciation | secondary symptom |
| 乏力 | weakness | - ^b^ | NA^c^ | weakness | primary symptom |
| 腹腔少量积液 | A small amount of ascites was observed in the abdominal cavity | - ^b^ | NA^c^ | ascites | secondary symptom |
| 苔白腻 | with a white greasy coating | - ^b^ | NA^c^ | white slippery coating | secondary symptom^a^ |
| 脉细弦 | the pulse was thin and wiry | wiry, rapid pulse | primary symptom | thin wiry pulse | secondary symptom^a^ |
| 上腹部疼痛 | abdominal pain | abdominal pain | primary symptom | - ^b^ | NA^c^ |
| 纳差 | poor appetite | loss of appetite | primary symptom | small appetite | secondary symptom |
| 大便偏稀，2-3次/日 | loose stools occurring 2-3 times per day | loose stools | primary symptom | - ^b^ | NA^c^ |
| 消瘦 | emaciated | - ^b^ | NA^c^ | Emaciation | secondary symptom |
| 皮肤巩膜黄染 | The skin and sclera showed jaundice | yellowish skin | primary symptom | - ^b^ | NA^c^ |
| 皮肤巩膜黄染 | The skin and sclera showed jaundice | yellow sclera | primary symptom | - ^b^ | NA^c^ |
| 苔白厚 | thick white coating on the tongue | - ^b^ | NA^c^ | white slippery coating | secondary symptom^a^ |
| 脉细弦 | a thin wiry pulse | wiry, rapid pulse | primary symptom | thin wiry pulse | secondary symptom^a^ |

^a^Features categorized as “Primary Tongue Indicators” and “Primary Pulse Indicators” are uniformly classified as “Primary Symptoms,” while features labeled as “Additional Tongue Indicators” and “Additional Pulse Indicators” are classified as “Secondary Symptoms.”

^b^The symbol “-” indicates that the feature does not correspond to any guideline content for the specified syndrome.

^c^NA, not applicable.
