# Supplementary material for: Identification of Syndrome Types in Patients With Pancreatic Cancer From Free Text in Electronic Medical Records: Model Development and Validation
Source: JMIR Form Res. 2025 Oct 3;9:e70602. doi: 10.2196/70602 (PMC12534766; doi:10.2196/70602)
Supplement: Multimedia Appendix 10 [file formative_v9i1e70602_app10.docx]

**Supplementary Table 7. TCM Diagnostic Features, Attribution Scores, and Correspondence with Guidelines.**

| **Original Case Record** | **Translation** | **Corresponding Guideline Content** | **Clinical Significance for Syndrome Differentiation** | **Highest Attribution Score for This Symptom** | **Average Attribution Score for Syndrome Differentiation Irrelevant Content** |
| --- | --- | --- | --- | --- | --- |
| **Damp-heat Syndrome** | | | | | |
| 上腹不适 | epigastric discomfort | abdominal pain / abdominal bloating | primary symptom | 0.204 | 0.047 |
| 胃纳一般 | decreased appetite | loss of appetite | primary symptom | -0.161 |  |
| 小便发黄 | yellowish urine | yellow urine | primary symptom | 0.622 |  |
| 大便颜色淡 | pale-colored stool | gray-white stool | primary symptom | 0.933 |  |
| 皮肤巩膜黄染 | yellowish skin and sclera | yellowish skin | primary symptom | 0.214 |  |
| 皮肤巩膜黄染 | yellowish skin and sclera | yellow sclera | primary symptom | 0.214 |  |
| 舌红 | the tongue was red | red tongue | secondary symptom^a^ | 1.009 |  |
| 苔薄黄 | thin yellow coating | yellow coating | primary symptom^a^ | 0.965 |  |
| 脉弦 | wiry pulse | wiry, rapid pulse | primary symptom^a^ | 0.084 |  |
| **Spleen-deficiency Syndrome** | | | | | |
| 精神欠佳 | mental fatigue | fatigue | primary symptom | 0.15 | 0.031 |
| 体重1月内减轻5公斤 | the patient's weight decreased by 5 kilograms within one month | emaciation | secondary symptom | 0.048 |  |
| 移动性浊音（+） | shifting dullness (+) | ascites | secondary symptom | 0.106 |  |
| 消瘦 | emaciation was observed | emaciation | secondary symptom | 0.368 |  |
| 纳呆 | indigestion | indigestion | primary symptom | 1.204 |  |
| 舌淡 | pale tongue | pale and swollen tongue | primary symptom^a^ | 0.592 |  |
| 苔白 | white tongue  coating | thin white coating | secondary symptom^a^ | 0.485 |  |
| 脉弦细 | thin wiry pulse | thin wiry pulse | secondary symptom^a^ | 0.022 |  |
| **Damp-heat with Spleen-deficiency Syndrome** | | | | | |
| 干呕频频 | frequent dry retching on admission | vomiting | primary symptom | 0.016 | 0.049 |
| 中上腹疼痛 | upper-middle abdominal pain | abdominal pain | primary symptom | 0.008 |  |
| 纳差 | loss of appetite | loss of appetite | primary symptom | 0.203 |  |
| 纳差 | loss of appetite | small appetite | secondary symptom | 0.203 |  |
| 大便溏薄 | loose stools | loose stools | primary symptom | 0.268 |  |
| 小便黄赤 | yellow urine | yellow urine | primary symptom | 0.403 |  |
| 体重较发病前减轻约6kg | The patient lost approximately 6 kg since disease onset | emaciation | secondary symptom | 0.18 |  |
| 皮肤巩膜中度黄染 | Moderate jaundice of the skin and sclera | yellowish skin | primary symptom | 0.053 |  |
| 皮肤巩膜中度黄染 | Moderate jaundice of the skin and sclera | yellow sclera | primary symptom | 0.053 |  |
| 舌红 | the tongue was red | red tongue | secondary symptom^a^ | 0.299 |  |
| 苔黄腻 | yellow greasy coating | yellow greasy coating | primary symptom^a^ | 0.382 |  |
| 脉弦 | wiry pulse | thin wiry pulse | secondary symptom^a^ | -0.014 |  |
| 脉弦 | wiry pulse | wiry, rapid pulse | primary symptom^a^ | -0.014 |  |

^a^Features categorized as “Primary Tongue Indicators” and “Primary Pulse Indicators” are uniformly classified as “Primary Symptoms,” while features labeled as “Additional Tongue Indicators” and “Additional Pulse Indicators” are classified as “Secondary Symptoms.”
