# Supplementary material for: Identification of Syndrome Types in Patients With Pancreatic Cancer From Free Text in Electronic Medical Records: Model Development and Validation
Source: JMIR Form Res. 2025 Oct 3;9:e70602. doi: 10.2196/70602 (PMC12534766; doi:10.2196/70602)
Supplement: Multimedia Appendix 11 [file formative_v9i1e70602_app11.docx]

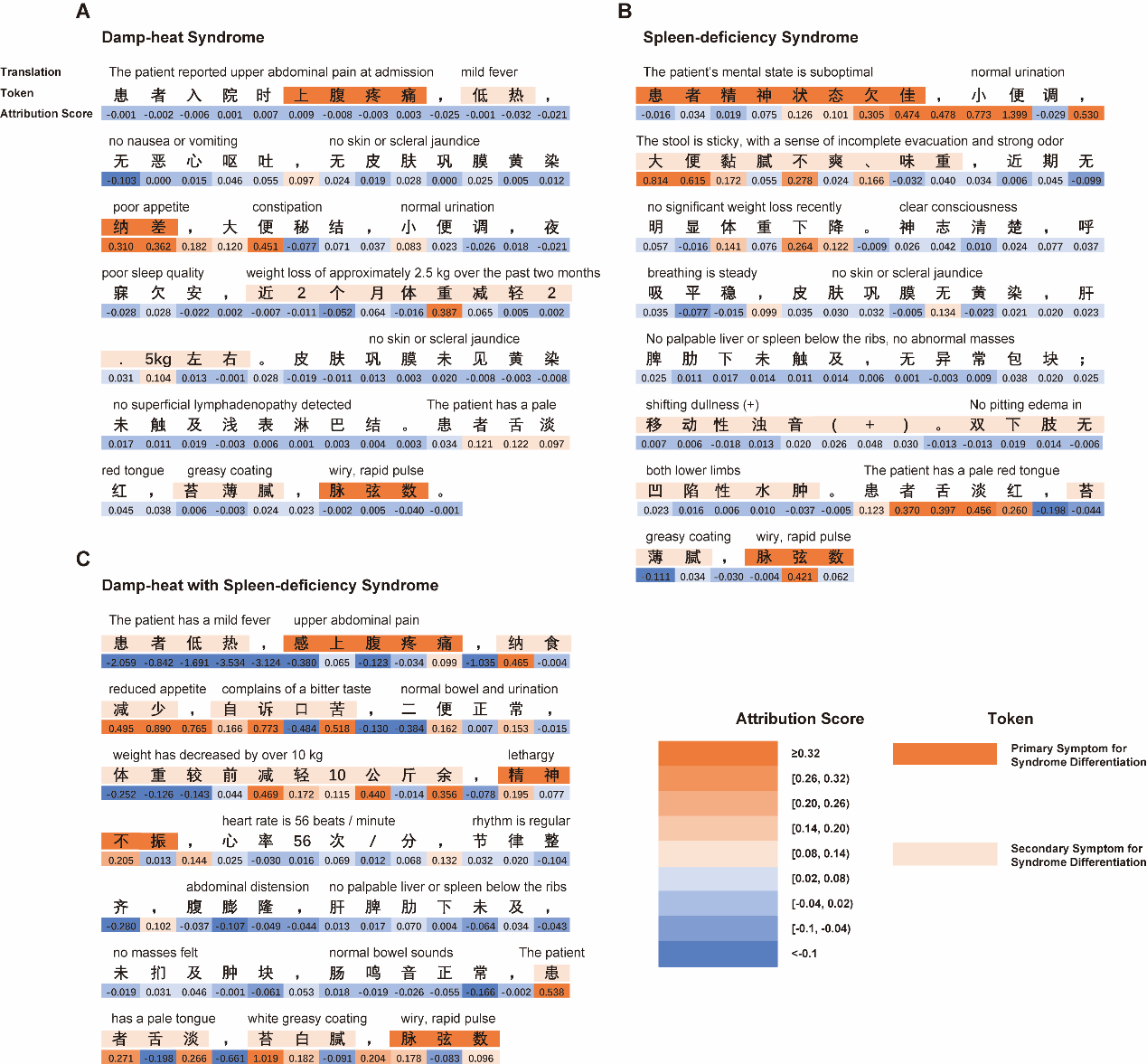


**Supplementary Figure1. Attribution Analysis of Misclassified Cases in TCMPCSD-BERT Model for Syndrome Differentiation.**

(A) A “damp-heat syndrome” case misclassified as “spleen-deficiency syndrome” by TCMPCSD-BERT. (B) A “spleen-deficiency syndrome” case misclassified as “damp-heat with spleen-deficiency syndrome” by TCMPCSD-BERT. (C) A “damp-heat with spleen-deficiency syndrome” case misclassified as “spleen-deficiency syndrome” by TCMPCSD-BERT.

**Supplementary Table 8. Summary of Key Diagnostic Features and Attribution Scores for Misclassified Cases in TCMPCSD-BERT Model.**

| **Original Case Record** | **Translation** | **Corresponding Guideline Content** | **Clinical Significance for Syndrome Differentiation** | **Highest Attribution Score for This Symptom** | **Average Attribution Score for Syndrome Differentiation Irrelevant Content** |
| --- | --- | --- | --- | --- | --- |
| **“Damp-heat Syndrome” misclassified as “Spleen-deficiency syndrome”** | | | | | |
| 上腹疼痛 | abdominal pain | abdominal pain | primary symptom | 0.009^b^ | 0.032 |
| 低热 | mild fever | persistent low-grade fever | secondary symptom | -0.001^b^ |  |
| 纳差 | poor appetite | loss of appetite | primary symptom | 0.362^c^ |  |
| 近2个月体重减轻2.5kg左右 | weight loss of approximately 2.5 kg over the past two months | emaciation | secondary symptom | 0.387^b^ |  |
| 苔薄腻 | greasy coating | thin greasy coating | secondary symptom^a^ | 0.024^c^ |  |
| 脉弦数 | wiry, rapid pulse | wiry, rapid pulse | primary symptom^a^ | 0.005^b^ |  |
| **“Spleen-deficiency Syndrome” misclassified as “Damp-heat with spleen-deficiency syndrome”** | | | | | |
| 精神状态欠佳 | mental state is suboptimal | fatigue | primary symptom | 0.474^c^ | 0.093 |
| 大便黏腻不爽、味重 | The stool is sticky, with a sense of incomplete evacuation and strong odor | foul-smelling loose stools | secondary symptom | 0.814^b^ |  |
| 移动性浊音（+） | shifting dullness (+) | ascites | secondary symptom | 0.048^b^ |  |
| 双下肢无凹陷性水肿 | no pitting edema in both lower limbs | edema in lower limbs | secondary symptom | 0.023^b^ |  |
| 苔薄腻 | greasy coating | thin greasy coating | secondary symptom^a^ | 0.034^b^ |  |
| 脉弦数 | wiry, rapid pulse | wiry, rapid pulse | primary symptom^a^ | 0.421^b^ |  |
| **“Damp-heat with Spleen-deficiency Syndrome” misclassified as “spleen-deficiency syndrome”** | | | | | |
| 低热 | mild fever | persistent low-grade fever | secondary symptom | -0.380^b^ | -0.065 |
| 上腹疼痛 | upper abdominal pain | abdominal pain | primary symptom | 0.099^b^ |  |
| 纳食减少 | reduced appetite | small appetite | secondary symptom | 0.890^c^ |  |
| 自诉口苦 | complains of a bitter taste | bitter taste | secondary symptom | 0.773^c^ |  |
| 体重较前减轻10公斤余 | weight has decreased by over 10 kg | emaciation | secondary symptom | 0.469^c^ |  |
| 精神不振 | lethargy | fatigue | primary symptom | 0.205^c^ |  |
| 舌淡 | pale tongue | pale tongue | secondary symptom | 0.266^c^ |  |
| 苔白腻 | white greasy coating | thin greasy coating | secondary symptom | 1.019^c^ |  |
| 脉弦数 | wiry, rapid pulse | wiry, rapid pulse | primary symptom^a^ | 0.178^c^ |  |

^a^Features categorized as “Primary Tongue Indicators” and “Primary Pulse Indicators” are uniformly classified as “Primary Symptoms,” while features labeled as “Additional Tongue Indicators” and “Additional Pulse Indicators” are classified as “Secondary Symptoms.”

^b^The attribution scores align with clinical judgment.

^c^The attribution scores do not align with clinical judgment.
