# Supplementary material for: Identification of Syndrome Types in Patients With Pancreatic Cancer From Free Text in Electronic Medical Records: Model Development and Validation
Source: JMIR Form Res. 2025 Oct 3;9:e70602. doi: 10.2196/70602 (PMC12534766; doi:10.2196/70602)
Supplement: Multimedia Appendix 12 [file formative_v9i1e70602_app12.docx]

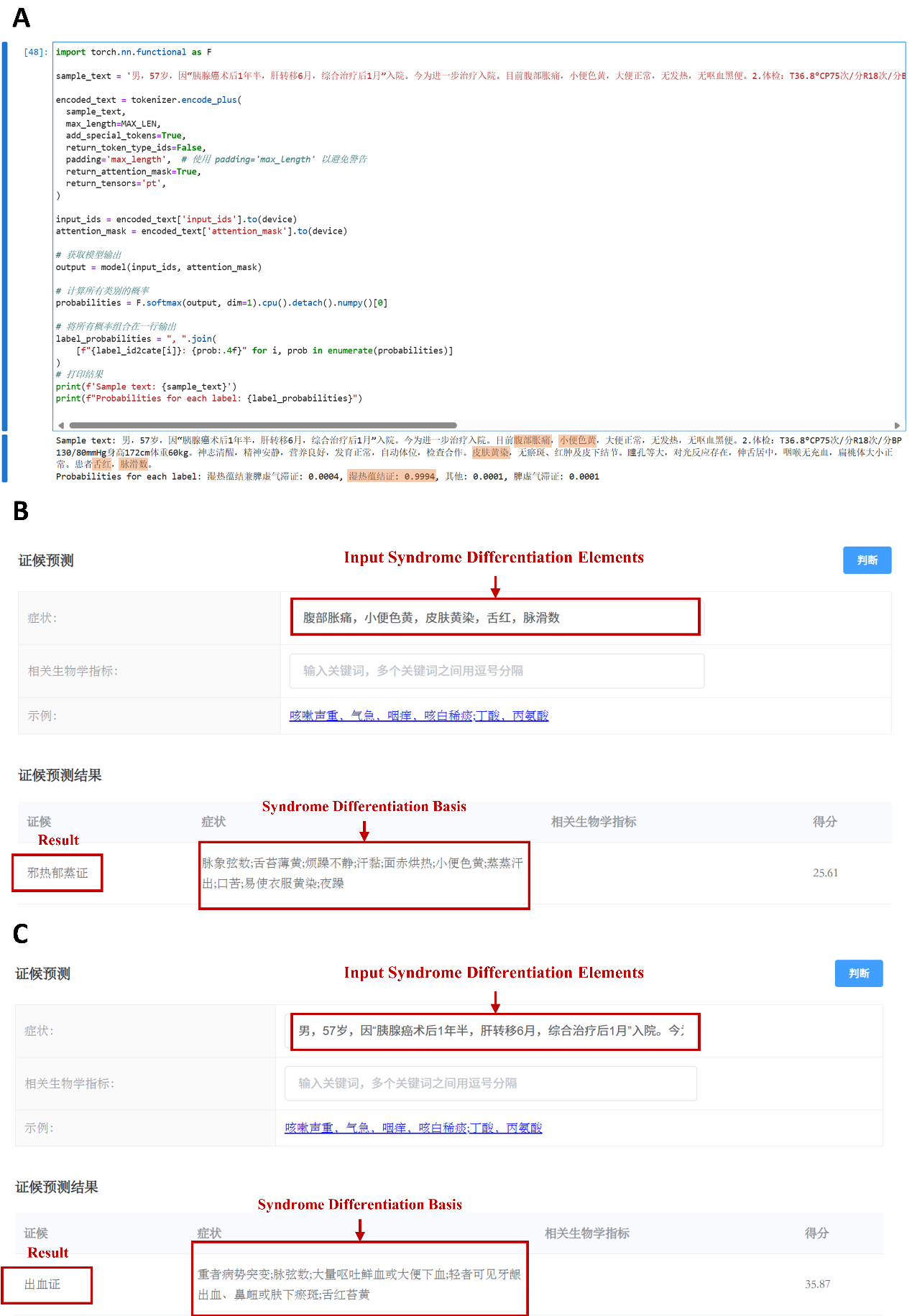


**Supplementary Figure 2. Comparison of Syndrome Differentiation Performance Between the Online Platform and TCMPCSD-BERT Model (damp-heat syndrome).**

(A) Syndrome differentiation result of the TCMPCSD-BERT model based on unstructured long text. (B) Syndrome differentiation result of the online platform based on structured content. (C) Syndrome differentiation result of the online platform based on unstructured long text.


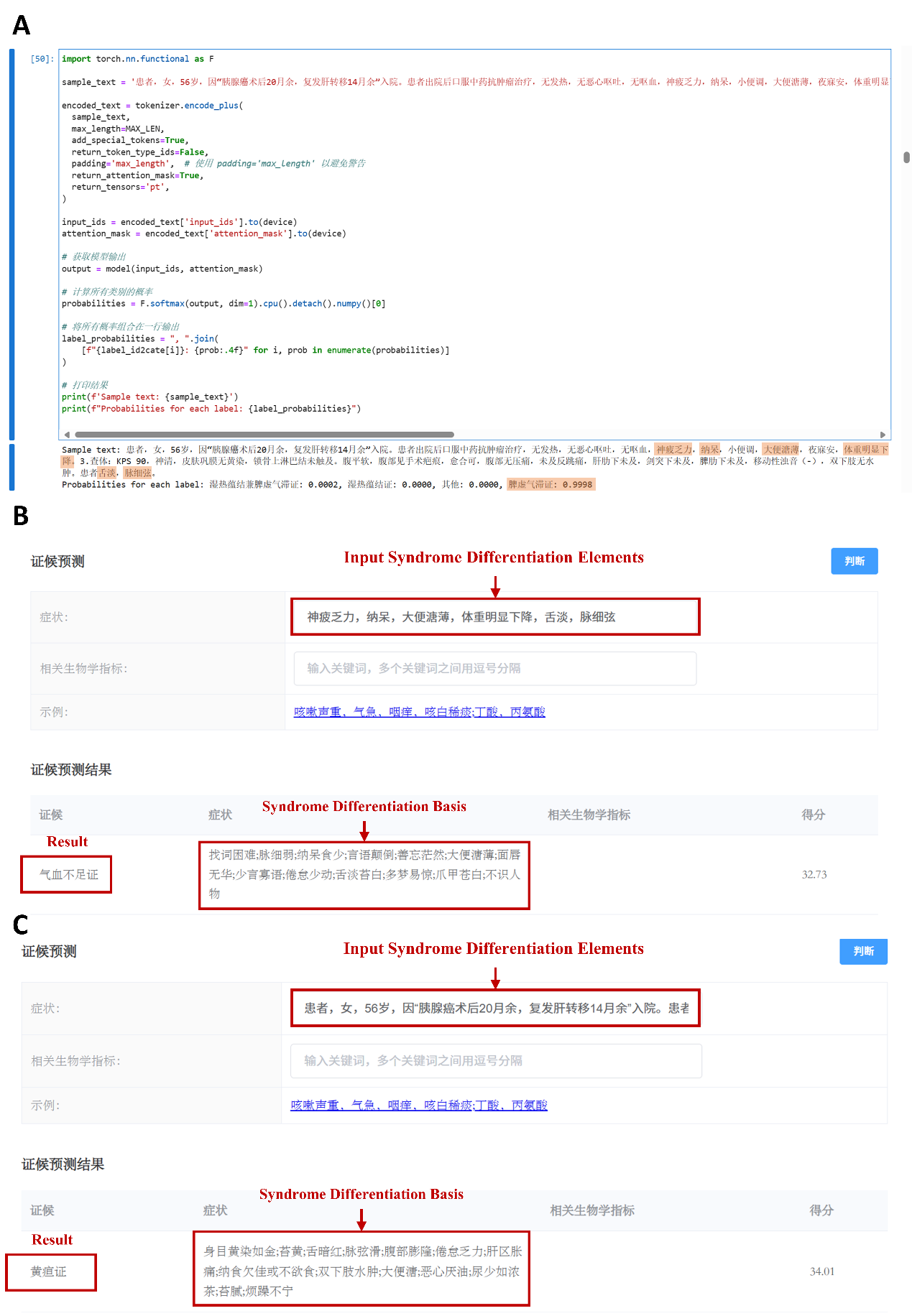


**Supplementary Figure 3. Comparison of Syndrome Differentiation Performance Between the Online Platform and TCMPCSD-BERT Model (spleen-deficiency syndrome).**

(A) Syndrome differentiation result of the TCMPCSD-BERT model based on unstructured long text. (B) Syndrome differentiation result of the online platform based on structured content. (C) Syndrome differentiation result of the online platform based on unstructured long text.


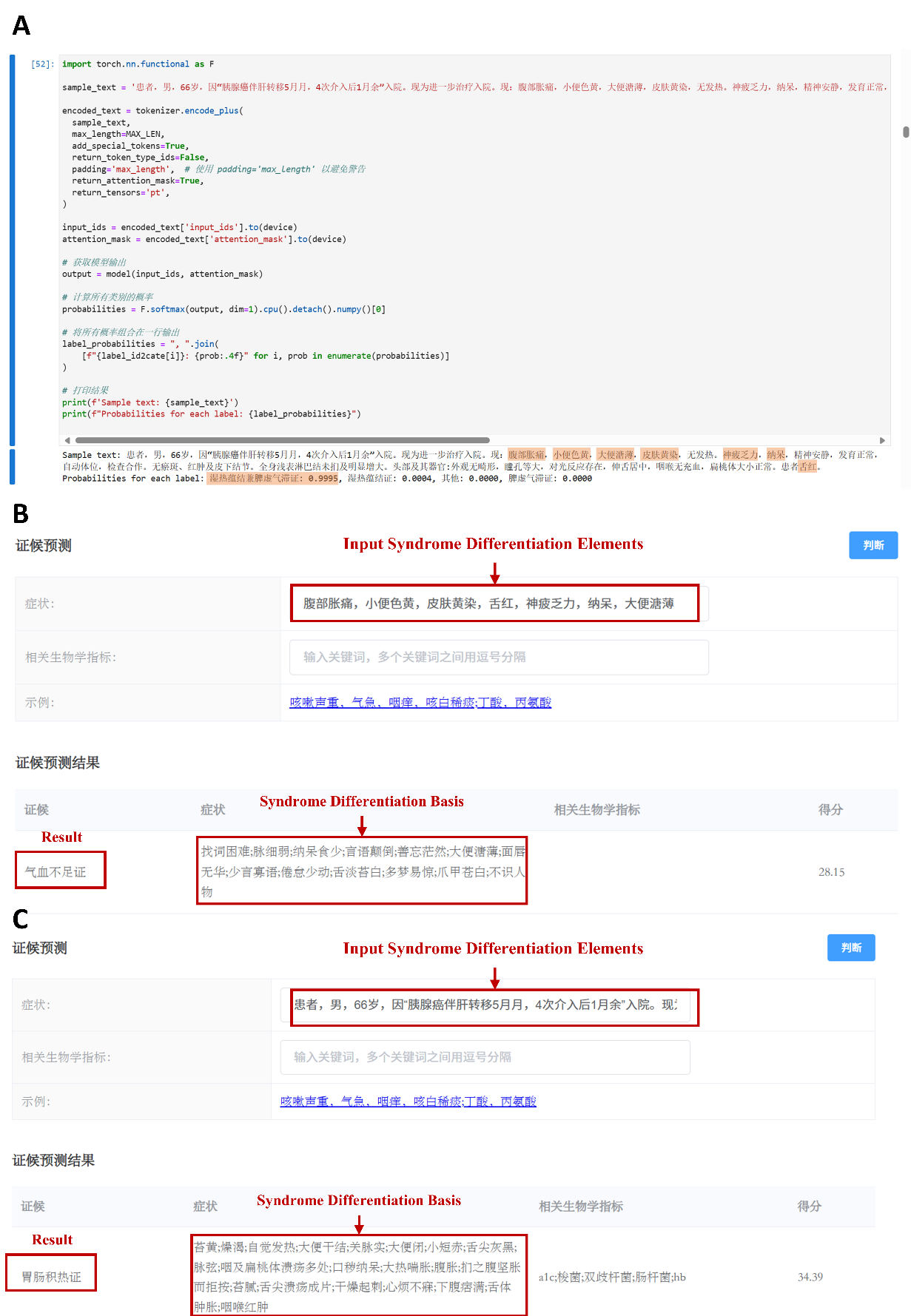


**Supplementary Figure 4. Comparison of Syndrome Differentiation Performance Between the Online Platform and TCMPCSD-BERT Model (damp-heat with spleen-deficiency syndrome).**

(A) Syndrome differentiation result of the TCMPCSD-BERT model based on unstructured long text. (B) Syndrome differentiation result of the online platform based on structured content. (C) Syndrome differentiation result of the online platform based on unstructured long text.


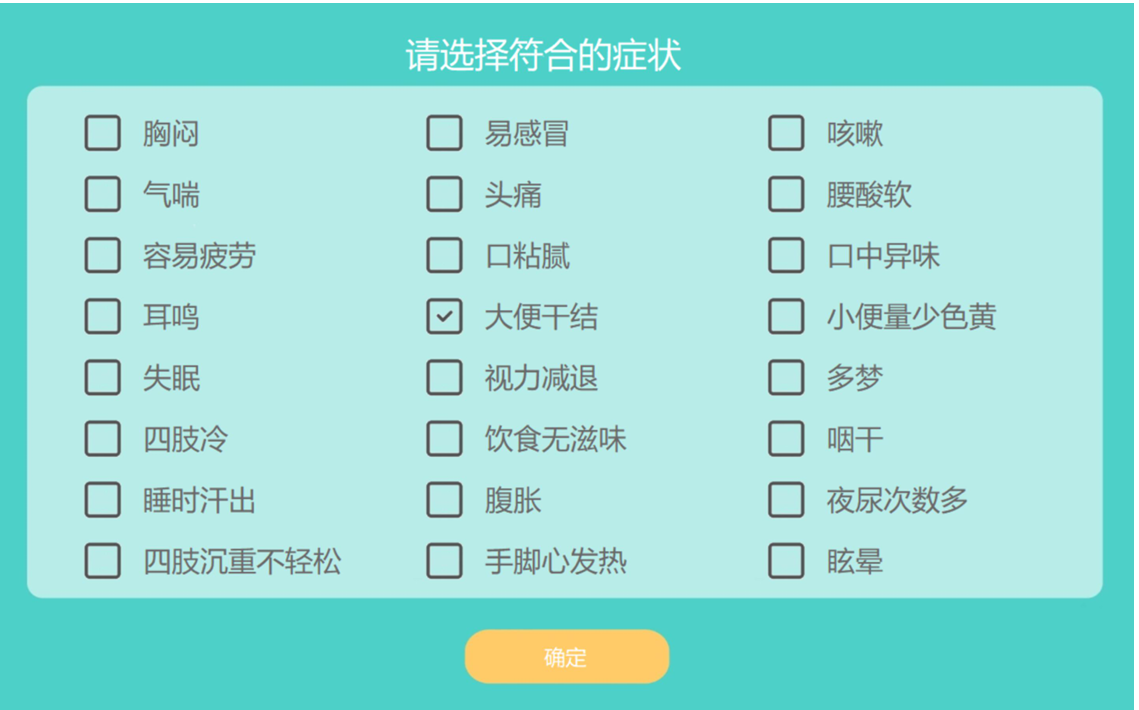


**Supplementary Figure 5. Syndrome Differentiation Operation Interface of the Four Diagnostic Instrument.**
