# Supplementary material for: Identification of Syndrome Types in Patients With Pancreatic Cancer From Free Text in Electronic Medical Records: Model Development and Validation
Source: JMIR Form Res. 2025 Oct 3;9:e70602. doi: 10.2196/70602 (PMC12534766; doi:10.2196/70602)
Supplement: Multimedia Appendix 13 [file formative_v9i1e70602_app13.docx]

**Feature Labeling in Expert Knowledge Learning for the TCMPCSD-BERT Model**

**Knowledge framework construction**

In this study, PCSD-BERT is designed to learn expert knowledge by identifying key features in the training data, focusing on elements such as knowledge framework construction, syndrome feature extraction, element weighting, and symptom severity assessment. The knowledge framework underlying PCSD-BERT is based on the latest expert consensus on syndrome differentiation for pancreatic cancer[1,2], establishing a foundational structure for the model (see information on feature engineering and data preprocessing can be found in Multimedia Appendix 2). This framework systematically organizes core elements and standards of the syndrome differentiation process, offering a structured guide for the model. However, in clinical practice, experts often tailor treatment for individual patients differ from clinical guidelines, particularly in TCM practices. For instance, an expert might integrate observations related to a patient’s specific characteristics—such as dietary habits, seasonal variations, environmental influences, and occupation—to enhance diagnostic accuracy. To enable the model to effectively learn the nuanced diagnostic expertise of specific practitioners, further customization of the framework is required to align it with individual experts’ diagnostic tendencies. This suggests that, during data collection, it is critical to prioritize cases recorded by experts themselves or documented under their supervision. Such records not only offer greater depth of information but also capture the precise considerations experts use in their diagnostic processes, thereby supporting the model in accurately capturing and replicating expert reasoning.

**Syndrome feature extraction**

A further challenge of PCSD-BERT model training is that TCM case records are often unstructured, with diverse symptom descriptions requiring contextual interpretation for accurate diagnostic reasoning. This inherent characteristic poses significant challenges for feature extraction, underscoring the importance of converting unstructured data into structured formats that align with expert consensus standards. In this study, clinical expertise was employed to interpret and standardize symptoms and signs in case records for syndrome label assignment (see Figure 1, 1.2–1.3). To ensure robust data quality and fidelity to clinical reasoning, it is recommended that data collection and model expansion prioritize cases documented or reviewed by experts, as these provide richer insight into diagnostic focal points from experienced practitioners’ perspectives. Additionally, while each case in the dataset was labeled with syndrome tags to serve as “reference answers” for model learning, the TCM field currently lacks unified diagnostic thresholds. Previous studies have proposed methods for quantifying the relationships between syndrome elements and syndrome types, such as the mutual information feature selection algorithm[3] for assessing syndrome element-syndrome associations, the Apriori algorithm[4] for uncovering these associations, or hypergeometric distribution enrichment testing[5] for patient classification. In the initial stages of this study, HEDT was also explored for feature extraction and syndrome classification. However, traditional methods like these are limited in their application to complex clinical data, as statistical measures such as *P*-values may fail to accurately reflect clinical realities. For instance, patients who meet diagnostic criteria in clinical practice may not achieve statistical significance in these models.

**Syndrome differentiation element weighting and symptom severity assessment**

Practically in TCM syndrome differentiation, the weight assigned to syndrome elements reflects not only their frequency or association strength but also their relevance to pathological characteristics, prognosis, and treatment outcomes. Currently, however, there is a lack of standardized system for weighting these elements, leaving this aspect largely dependent on clinical expertise. In this study, we apply a weighting system guided by expert consensus on pancreatic cancer syndrome differentiation (e.g., “Primary Symptom” and “Secondary Symptom” in Figure 5)[1,2], aligning the model with established clinical standards during training. This expert-informed approach enhances the model’s alignment with practitioner logic. However, relying solely on consensus may limit the model’s adaptability to the individualized needs of clinical practice, as the importance of specific elements can vary based on patient characteristics and disease progression. In the evaluation of complex clinical scenarios, such as evaluating combined syndromes or determining the significance of specific syndrome elements often hinges on symptom severity, often result in alteration of treatment plans. While this study’s model training primarily follows an expert consensus framework, symptom severity is particularly important for more complex syndrome classification and differentiation tasks. Therefore, the documentation of symptom severity, frequency, and trends is of importance to support the model in comprehensively learning and interpreting disease progression trajectories. To further optimize the model, incorporating diverse expert judgment criteria across various clinical scenarios is valuable allowing capture of the relative importance of syndrome elements in new contexts. Additional details of symptoms severity, and frequency, along with syndrome labels that reflect practical clinical nuances collected during data acquisition will provide future model with refined guidance in learning and applying syndrome elements, improving its effectiveness across a wide range of clinical cases.

**Syndrome differentiation thresholds setting**

In the setting of syndrome differentiation thresholds based on role of expert consensus, while statistical significance provides an objective basis for defining syndrome differentiation labels, it may overlook clinical complexities, thus not fully representing a patient’s condition. To address this, our study relies on expert consensus in pancreatic cancer syndrome differentiation[1,2] to establish differentiation thresholds. Future studies can refine and expand these standards by integrating additional clinical trials and data, ultimately enhancing model-based decision-making in practical applications. Together, the model’s capacity to learn from expert experience depends not only on a systematic knowledge framework and standardized feature extraction but also on the careful calibration of syndrome weightings and symptom severity thresholds. Therefore, these components form the foundation of the model’s ability to emulate expert judgment, guiding data collection and feature engineering to better capture and apply the nuanced insights of seasoned clinicians.

1. Hou W. *Integrated Chinese and Western Medicine in Oncology*. Beijing: People's Medical Publishing House; 2022.978-7-117-34044-1: 978-7-117-34044-1.

2. Fang Z. *Principles of Syndrome Differentiation and Treatment in TCM*. Shanghai: Shanghai University of Traditional Chinese Medicine Press; 2008.7-81121-035-5: 7-81121-035-5.

3. Li M, Wang L, Wu Q, Zhu J, Zhang M. Diagnosis knowledge constrained network based on first-order logic for syndrome differentiation. Artif Intell Med. Jan 2024;147:102739. doi:10.1016/j.artmed.2023.102739

4. Li Y, Zhang XY, He SL, Zhao YC, Liu R, Hua BJ. [Clinical symptoms and distribution characteristics traditional Chinese medicine syndromes of pulmonary nodules]. Zhongguo Zhong Yao Za Zhi. Sep 2023;48(17):4782-4788. doi:10.19540/j.cnki.cjcmm.20230606.501

5. Qi Y, Liu Y, Liu X, Li J, Qi S, Zhang Z. Identification of risk factors and clinical model construction of abdominal distension after radical cystectomy. Transl Androl Urol. Dec 2022;11(12):1629-1636. doi:10.21037/tau-22-455
